# Abstracts from the 12th Asia Pacific Burn Congress: Singapore. 14-17 August 2019

**DOI:** 10.1186/s41038-019-0164-1

**Published:** 2019-08-13

**Foreword 1**

Si Jack Chong

Organising Co-chairperson | 12th Asia Pacific Burn Congress

President | Association for Burn Injuries Singapore

President | Asia Pacific Burns Association

APBC is the official meeting of APBA

Members of the Asia Pacific Burn Association (APBA) have been managing the bulk of the World’s burnload over the years. Generations of burn care professional in the Asia Pacific region have devoted their life to develop and enhance burn care the burn patient in the region. They have pioneered many innovations and introduced many techniques to the world of burn managements. Members have shared their experiences and latest research at the past Asia Pacific Burn Congresses(APBC) since it was first held in Singapore in 1992.

The organising committee of the 12^th^ APBC is pleased to collaborate with the board and editorial team of *Burns & Trauma* to select and collectively publish 132 abstracts as a supplemental issue of *Burns & Trauma* in Aug 2019 and distributed in conjunction with the 12^th^ APBC held in Singapore. Among all submissions five excellent papers will win *Burns & Trauma* best article awards.

*Burns & Trauma* is the first peer-reviewed Open Access journal devoted to basic, clinical and translational research related to burns and traumatic injuries in the region. It is indexed by the PubMed and SCIE, with the Impact Factor of 2.493. The journal offers a fast publication schedule whilst maintaining rigorous peer review. All articles published by *Burns & Trauma* are made freely and permanently accessible online immediately upon publication.

I strongly believe that this association between APBC and *Burns & Trauma* is a win-win relationship. Having their work published in Burns and Trauma will undoubtedly spur and motivate researchers in their endeavours in the field of burn research. It is also beneficial for *Burns & Trauma* to engage and build lasting partnership with researchers in the Asia Pacfic region.

In all, I am pleased to be part of the team effort to launch the inaugural supplemental issue for *Burns & Trauma*. I sincerely hope that the Asia Pacific Burn Care community will embrace this publication.

**Foreword 2**

Gaoxing Luo

Director of Institute of Burn Research, Southwest Hospital, Army Medical University, China

Associate EIC, Burns & Trauma

Guangping Liang

Managing Editor, Burns & Trauma

Managing Editor, Chinese Journal of Burns

Burns & Trauma, the official journal of Chinese Burn Association, has just got its first impact factor of 2.493 in 20th June, 2019. As the sponsor of the journal, we deeply appreciate the Editor-in Chief, Prof. Jun Wu and his forward-looking to found the journal in 2013. We also appreciate the great support by the former Deputy Editor Prof. David Mackie, Naiem Moiemen, and current Deputy Editor Prof. Tina Palmieri, as well as all efforts made by the editorial board. Surely, the editorial office team should be remembered for their hard work and great contribution for the Journal. Through years of development, and active cooperation with various international organizations like APBA, Burns & Trauma has become a good academic platform for knowledge and idea exchanges in the fields of burns and trauma.

As the only Open Access journal in Asia focusing on burns, all the contents could be freely read and download in the journal homepage (www.burnstrauma.com). Here, we are happy for the first time to initiate an abstract supplement for the 12th APBC 2019, and set APBC - Burns & Trauma best paper awards, aiming to encourage young investigators to devote more into clinical and basic burn researches. We hope this salutary attempt will be a good start, which will not only promote the journal but also enlarge the influence of APBC.

With the development of new scientific technology, the emphasis of burn care has transferred gradually from traditional treatment of improving the mortality rate into prevention and outcome qualities. Researches not only on critical care, but also on scar management, tissue engineering and regeneration, and rehabilitation have been becoming hotter and hotter. Burns & Trauma has published several special issues on these hot topics, High quality research papers related to the following special series are warmly welcomed for submission: Facial & Hand burns, Inflammation & Wound healing, Nutrition & Metabolism, Scar, Stem cells & Tissue engineering, etc.

Moreover, we’d like to give our sincere thanks to the president of the 12th APBC, Prof. Chong Si Jack, who is also the editorial board member of Burns & Trauma, to provide the opportunity for deep bilateral cooperation between the journal and the congress. This win-win action will help the journal get wider audience, while also enlarge APBC influence in China and world-wide.

Let every one of us enjoy the grand congress, and have a fruitful gaining in Singapore.

**Foreword 3**

Jun Wu

Editor-in-Chief, Burns & Trauma

Since launched in June 2013, *Burns & Trauma* remains focused on publishing the latest developments in basic, clinical and translational research related to burns and traumatic injuries. As the official Journal of Chinese Burn Association, Chinese Society for Scar Medicine and Chinese Burns Care & Rehabilitation Association, *Burns & Trauma* is the first peer-reviewed OA journal in the related fields which devoted to report burn researches. After indexed by the PubMed in2015, the journal was included in the SCIE in 2018 and gained the first Impact Factor of 2.493 in 2019.

With cooperation with various international associations *Burns & Trauma* tries to provide a platform for burn researchers and doctors to present their scientific achievements and exchange their ideas. We have organized 11 special series covering the hot topics in the field of burns and trauma, such as scar management, cell therapy & skin regeneration, 3D bio-printing, pediatric burns, etc., inviting prestigious scholars to review the most advanced technologies and progress in the related fields, which aims to provide new perspectives and knowledge in the field of burns. Now we are delighted to join hands with the organizing committee of the 12^th^ Asia Pacific Burn Congress (APBC) to publish 132 abstracts as a supplement of *Burns &Trauma*, and select best original papers for the possibility to be published in the journal.

The consociation with Asia Pacific Burn Association (APBA) is a successful and beneficial attempt for both sides. Publishing the abstracts of the delegates will surely motivate their enthusiasm for further researches and make *Burns & Trauma* better known in Asia Pacific region.

Finally, I wish the 12^th^ APBC a complete success and hope the inaugural supplemental issue launched smoothly.

**12** ^**th**^ **Asia Pacific Burn Congress Scientific Committee**

Si Jack Chong (Chairperson)

Alvin Wen Choong Chua (Chairperson)

Kala Adaikan

Hong Ngee Chan

Kong Yik Chew

Jasmine Chiat Ling Ong

Maciej Piotr Chlebicki

Annabelle Soo Ching Ip

Cecilia Sze Nga Kwok

Gale Jue Shuang Lim

Pei Fen Seah

Anna Chew Na Tan

Emily U-Tong Tan

Chai Lian Teng

Sanda Thangarajoo

## Speaker presentation

### Abstract Topic: Burn Care in Pre hospital & Austere Environment

#### S1 Actualities and thinking on early management of mass burn casualty disaster in China

##### Guodong Song^1,2^, Naijun Xin^1,2^

###### ^1^Department of Burns and Plastic Surgery, Jinan Central Hospital Affiliated to Shandong University, Jinan, Shandong, PR China; ^2^First Aid Center for Burns in Jinan, Jinan, Shandong, PR China

####### **Correspondence:** Guodong Song

**Background**

Management of mass burn casualty disaster (MBCD) is both medical and social problems. Field first aid, triage transfer and early care is the critical stage of MBCD management. This paper presents an analysis of the recent experiences and lessons of MBCD management with the aim of proposing relevant countermeasures for the problems in several key links in early management of MBCD in China.

**Materials and Methods**

Literature review including systematic searches of PubMed/Medline and CBMdisc, representative monographs and landmark articles.

**Results**

Burns department should be incorporated into medical rescue contingency plans of local public emergencies, and burn specialty team should be organized. Triage and primary triage transfer is the first link of medical emergency at the disaster scene or at the emergency room of the first receiving hospital. The disaster site incident headquarters should call the nearest burns department and the hospital with the necessary care competence according to the situation of triage and evacuate of casualty. For the MBCD that can be not rapidly shunted nearby, especially the severely burned patients, the headquarters should coordinate and organize protraction of specialized technical force, and then organize to implement the normative early management in local hospital. After spending steadily the shock stage or making up blood volume, the burn patients should be secondarily triaged and transferred to the burn center in soonest possible. Normative fluid resuscitation should be persistently performed in each links of MBCD triage and transfer. The treatment strategies for extensive deep burns wound of early MBCD should aim at stabilizing and controlling patient’s condition firstly, carry out the concept of damage control operation, and perform tangential excision and alloskin or xenoskin grafting for temporary coverage and later on autoskin grafting.

**Conclusions**

Rescue contingency plans and early management on MBCD in China should be further improved.

### Abstract Topic: Burn Care in Pre hospital & Austere Environment

#### S2 Transport and early treatment of molten Steel Burn in batches

##### Shutang Zhang, Qi Liu

###### Department of Burn and Reconstruction surgery, The Affiliated Hospital of Zhengzhou University, China

####### **Correspondence:** Shutang Zhang

**Background**

Further discussion on the early treatment measures of a batch of severe patients burned by explosive molten steel.

**Materials and Methods**

Summarize the body condition, transshipment, treatment process and experience of 13 explosive molten steel burned patients in the sudden explosion in a steel mill, 17^th^ may 2018. Scientific and rational double-echelon management, multi-departments’ and multidisciplinary comprehensive diagnosis and treatment mode, establishment of a rapid and effective early transport and treatment system, rehydration scheme based on patient treatment response and early cutting and skin grafting were mainly adopted as the rescue and early treatment.

**Results**

The County Emergency Office and Center immediately organized first aid for patients after the explosion, and reported to the City Health Planning Commission at the same time. They immediately organized Nanshiyoutian Hospital to start ambulances and helicopters to go to the scene for rescue. About 5 hours after the injury, 13 severe burned patients were transferred to the hospital for treatment. The success rate of transport was 100%. Two wounded died within 24 hours of hospitalization due to traumatic brain injury and deep burn of 100% TBSA. There were 9 especially severely burned and 2 severely burned among the remaining 11 patients. Six of the 9 especially severely burned had burn wounds of 80-100% TBSA. The 1-week survival rate was 100%, the 2-week survival rate was 90.9%, and the 1-month survival rate was 72.7%. The hospital discharge rate and survival rate were 54.5% after 4 months

**Conclusions**The effective organization of relevant departments, the joint transportation of air and land, the seamless connection of transshipment and in-hospital consultation, the multi-departments' and multi-disciplinary collaborative treatment after the batches of burn accident can significantly improve the prognosis of patients and reduce the serious consequences caused by explosive damage.

### Abstract Topic: Burns Education

#### S3 A look at the effectiveness of burns management in Dhaka, a fast-developing capital.

##### Low Tzuemn Lin^1^, Chong Xin Ying^3^, Foong Shyn Yih Hayley^3^, Tanveer Ahmed Himon^2^, Chong Si Jack^3^

###### ^1^Ministry of Health Holdings, Singapore; ^2^Department of Plastic Surgery and Burn Unit, Dhaka Medical College and Hospital, Dhaka, Bangladesh; ^3^Asia Pacific Burn Association, Bali, Indonesia

####### **Correspondence:** Low Tzuemn Lin

**Background**

Burn injuries are prevalent and devastating, be it for the patient, their family, the community or the country. The effects are far-reaching. With proper measures, ranging from prevention to rehabilitation, burn outcomes can be dramatically improved. This study aims to look at the effectiveness of burns management in Dhaka, a fast-developing capital.

**Materials and Methods**

Interviews were conducted one-to-one with a translator present at all times, specifically targeting nurses. Nurses were randomly selected while they were working in the Burns Unit at Dhaka Medical College Hospital. Verbal consent was taken prior to the interview.

**Results**30 Nurses participated in the survey. 20% were males while 80% are females. 70% (21) of their language medium of choice was English. 10% (3) was other languages and 20% (6) was Bengali. The types of burn injuries witnessed by this group of nurses include thermal (28), electrical (19), chemical (16), friction (15), radiation (2), lightning (2) and cold injury/frost bite (1). Thermal injuries were frequently caused by fire from cooking stove and open fires. The most common cause was poor layout in terms of house kitchen as well as city planning. Stoves were placed hazardously in households, often in make-shift kitchens and sometimes on the floor. It did not help that women wore sarees. There was the practice of burning rubbish in the open, at will, with no designated area, safety rules or regulations from the government. Also, women were known to practise perineal warming after giving birth by sitting over or near fires. 2 nurses attributed poor burn outcomes to religious practices, stating that Muslim women had the tendency to present late for treatment. There was also inadequate understanding of first aid among the community. Majority (23) thought that inadequate general public awareness was the most important social cause for burns. The complication of burns which patients complained the most was infection. In order of importance, nurses were ranked the most important in spreading awareness, followed by patients, then doctors, then teachers/instructors, state governors and lastly country politicians. 17 (56.7%) thought that doctors, instead of nurses should lead burns resuscitation whereas 29 (96.7%) thought that nurses should lead burns dressings.

**Conclusions**

Without a doubt, burns burden is high in Dhaka. Burns will not be managed effectively if one neglect the importance of primary prevention. Cultural practices need to be respected when formulating solutions. General public education can be reinforced, either by introducing burn programs into the school curriculum or broadcasting awareness clips in the social media. Measures need to be taken to reduce infection rates in burns.

### Abstract Topic : Burns Education

#### S4 Inhalational Injury – A Pictorial Grading

##### Delong Zeng^1^, Suneel Ramesh Desai^2^, Chong Si Jack^3^

###### ^1^Department of Plastic, Reconstructive and Aesthetics Surgery, Singapore General Hospital, Singapore; ^2^Department of Anesthesiology and Surgical Intensive Care, Singapore General Hospital, Singapore; ^3^Asia Pacific Burn Association, Bali, Indonesia

####### **Correspondence:** Delong Zeng

**Background**

Inhalational injury as a cause of burns, or explosions can have serious consequences if not identified quickly and managed properly. Early surveillance of airway is essential in managing their complications. The Abbreviated Injury Score (AIS) for bronchoscopic evaluation is used for grading the severity of inhalational burns. This study aims to provide a visual representation of the different AIS grades to aid in airway evaluation, as well as, propose a workflow for anyone that comes to the Accident and Emergency (A&E) department with possible inhalational burns.

**Materials and Methods**

Patients with varying degrees of inhalational injury were selected to represent the AIS grading. No patient was identified as AIS 4, therefore, a standard picture was used. These patients all underwent early evaluation of their airway with nasolaryngoscopy, followed by more detailed assessment with bronchoscopy.

**Results**

A pathway is devised for the management of patients with varying degrees of inhalational burn. Pictures are incorporated into the workflow to aid in visual guidance.

**Conclusions**

Clinical signs of airway injury can be non-specific, therefore, an upper airway nasolaryngoscopy is essential to evaluate the need for intubation. Lower airway bronchoscopy can be used to predict the likelihood of successful early extubation, or complications such as Acute Respiratory Distress Syndrome or pneumonia. A pictorial grading of inhalational injury, together with our management pathway, will greatly help the speed and effectiveness of treating burn patients when they first arrive to the hospital.

### Abstract Topic: Burns Education

#### S5 A 5-year retrospective study of liquefied petroleum gas-related burn patients in China

##### Ronghua Jin, Jon Kee Ho, Chunmao Han

###### Department of Burns & Wound Care Center, Second Affiliated Hospital of Zhejiang University, College of Medicine, Hangzhou, China

####### **Correspondence:** Chunmao Han

**Background**

Liquefied petroleum gas (LPG) is a widely used environment-friendly fuel. According to the annual report of World Liquefied Petroleum Gas Association in 2015, China has become the world’s second largest LPG-consuming country. Previous studies have shown an increasing number of LPG-related burns [1]. Our study was designed to evaluate the epidemiologic pattern of these injuries and provide recommendations for burn prevention.

**Materials and Methods**

Patient data was collected from the clinical database of patients with LPG-related burns from burn units of eight different hospitals in Zhejiang Province from January 2011 to December 2015. Database variables included patient demographics, accident characteristics and injury characteristics.

**Results**

This retrospective study included all patients with LPG-related burns in eight burn centers between 2011 and 2015. A total of 1898 patients were included, 47.31% were male and 52.69% were female. The predominant age group was 31~70 years (74.50%), majority were poorly educated and the incidence peaked from June to September. The most common place of occurrence was home (74.08%) and gas leak was the most common cause. The four limbs (43.33%) were the most frequently affected areas, the mean burn area was 25.19±20.97% of the total body surface area (TBSA) and most patients (46.89%) suffered from moderate burns. The mean length of hospital stay was 17.66±16.55 days and majority of patients (89.36%) recovered with 0.84% mortality rate.

**Conclusions**

Our findings reflected that the increase in incidence rate was alarming. Therefore, this calls for simple but strict measures to prevent these burn injuries. Gas leak was the main cause of LPG-related burns, any part of LPG stove system that shows signs of weathering should be replaced regularly.

**References**

[1] Jin Ronghua, Wu Pan, Ho Jon Kee, et al. Five-year epidemiology of liquefied petroleum gas related burns. Burns. 2018; 44:210-217.

### Abstract Topic: Burns Education

#### S6 Evaluation of a multidisciplinary burns training program in Bangladesh

##### Anna Tan, Florence Chiang

###### Occupational Therapy Department, Singapore General Hospital, Singapore

####### **Correspondence:** Anna Tan

**Background**

Burns is one of the most common injury in Bangladesh. The huge number of patients coupled with limited number of designated hospitals and skilled manpower resulted in death or severe complication in burns survivors. As such, the government had planned for a new designated burns hospital to meet national needs, and a multidisciplinary burns team in Singapore has agreed to provide training to build the capacity of specialised healthcare workforce for this new hospital and beyond. Training consists of a 5-day on-site teaching course that was planned to run 5 times over 3 years. It aims to train 310 nursing, doctor and rehabilitation specialists who in turn will train another 900 healthcare workers. The 5-day on-site training consists of lectures; live surgery demonstration; splinting workshop (hands-on); wound dressing workshop (hands-on); case scenario discussion and group presentations. A handbook was also published as training material to aid the delivery of the information. Curriculum is planned with inputs from both the Dhaka burns team and Singapore burns team. The aim of this paper is to evaluate the effectiveness of the training course.

**Materials and Methods**

The course was evaluated based on the Kirkpatrick’s model of course evaluation. Data was collected on the participants’ reactions to the training with a post training questionnaire. A pre and post-course test was also conducted to determine the participants’ extent of knowledge gained.

**Results**

One hundred and forty-six participants attended the course. Seventy-four participants completed the post training questionnaire with 97 % of them rated the course as meeting or exceeding their expectation. Out of 79 participants who completed the pre and post-course test, 86% of them showed an improvement with 29% of them achieving an improvement of 30% in their test scores.

**Conclusions**

The training has led to an improvement of knowledge among participants.

### Abstract Topic: Burns Education

#### S7 Correlation Of Extended-Spectrum Beta-Lactamase Infection With Mortality In Burn Patients In Dr. Soetomo Hospital Burn Centre From 2015-2018

##### Iswinarno Doso Saputro^1^, Lobredia Zarasade^2^, Caesarani Kristel^3^

###### ^1^Senior Staff of Department of Plastic Reconstructive and Aesthetic Surgery, Airlangga University, Dr. Soetomo Hospital, Surabaya; ^2^Senior Staff of Department of Plastic Reconstructive and Aesthetic Surgery, Airlangga University, Dr. Soetomo Hospital, Surabaya; ^3^Resident of Department of Plastic Reconstructive and Aesthetic Surgery, Airlangga University, Dr. Soetomo Hospital

####### **Correspondence:** Iswinarno Doso Saputro

**Background**

Burns results in suffering throughout the life of a person with high mortality and morbidity. Some studies suggest that infection of extended-spectrum beta-lactamase (ESBL) producing strains increase the mortality rate in burn patients. Several studies have been carried out, but no complete data has been obtained at Dr. Soetomo Hospital. This study was conducted to look at the correlation of ESBL infection with mortality in burn patients in Dr. Soetomo Hospital, Surabaya

**Materials and Methods**

A cross sectional study of patients with burns admitted to the Burn Centre of Dr. Soetomo Hospital between January 2015 and December 2018 were evaluated.

**Results**

313 subjects were involved in this study. 104 subjects are female (33,2%) 209 subjects are male (66,8%). There were 76 subjects with 1-19 years of age, 9 of them died. 29 subjects with extreme age (<1 year old or > 60 years old), 13 of whom died. 62% of the burn injuries are caused by fire (195 subjects), 16% caused by electricity (50 subjects), 13% caused by scald (41 subjects). Inhalation injury was found in 118 subjects (37,7%) and 46 of them died, acute kidney injury in 37 subjects (11,8%) and 25 of them died, sepsis in 50 subjects (16%) and 23 of them died. Out of 313 subjects, 75 subjects (24%) died, 62 subjects (82,7%) of it caused by fire, and 5 of them were infected with ESBL. Out of 5 subjects died with ESBL infection, 2 of them with acute kidney injury, 4 of them with sepsis, and 4 of them with inhalation trauma.

**Conclusions**

In 4 years period, 313 subjects were obtained, 19 subjects were infected with ESBL, 5 (26,3%) of them died. Mortality rates increases if ESBL infection is accompanied by other comorbidities such as acute kidney injury, sepsis, and inhalation trauma.

### Abstract Topic: Critical Care/Resuscitation and Pharmacology

#### S8 Does Parkland Formula Remain As The Best Chosen Formula For Initial Treatment In Patients With Burn Injury?: A Systematic Review

##### Nadhira Anindita Ralena^1^, R. Aditya Wardhana^2^

###### ^1^Faculty of Medicine, University of Indonesia, Jakarta, Indonesia; ^2^Plastic and Reconstructive Surgery Department, Cipto Mangunkusumo Hospital, Jakarta, Indonesia

####### **Correspondence:** Nadhira Anindita Ralena

**Background**

Burns can lead to abundant fatalities. It may cause temporary and permanent disability. Initial fluid resuscitation is required to support burn patients. It is significantly beneficial for the patients, especially on the first 24 hours. In majority of cases, Parkland Formula is used. However, fluid administration based only on Parkland Formula could lead to less output of urine at certain conditions.

**Materials and Methods**

A comprehensive literature searching was done by the authors starting from October 25th 2018 using electronic databases PubMed, Medline, Scopus, Embase, and Cochrane with key words including adults, Parkland Formula and urine output. Authors finally gathered 8 relevant articles. Cohort studies were critically appraised with Newcastle-Ottawa Quality Assessment and randomized controlled trials used PEDro Scale.

**Results**

Literatures related to Parkland's Formula with its effects on urine output varying greatly. The types of studies consisted from cohort to randomized controlled trials. The subjects used also differ from human to adult animals, with several comparison objects, such as administration of intravenous hypertonic fluid, rehydrated oral fluids and bolus directly to the colon through the anus. Addition or reduction in the speed of entering fluid was often done and adjusted for the amount of urine output. There is no research that only relied to the 2-4 ml*weight*TBSA% formula. Increasing the infusion rate after the first 8 hours of 24-hour fluid resuscitation could significantly maintain the amount of urine output by 0.5–1.0 ml/kg/hour. One study showed that hyperdynamic fluid resuscitation could significantly increase urine expenditure in the first 8 hours of subjects with 60% TBSA burns. Another trial concluded that the provision of WHO-ORS with Sørensen formula was effective in patients with 15% TBSA burns and could produce urine output as targeted.

**Conclusions**

From all the results shown, it can be directed that the Parkland Formula alone cannot be used as a standard.

### Abstract Topic: Critical Care/Resuscitation and Pharmacology

#### S9 Role Of Acute Kidney Injury In Severely Burned Patients

##### Zhiqiang Yuan, Bo You, Gaoxing Luo, Yizhi Peng

###### Institute Of Burn Research, Southwest Hospital,Army Military Medical University,Chongqing,China

####### **Correspondence:** Zhiqiang Yuan

**Background**

The aim of this study was to describe the epidemiological, clinical and pathological characteristics of acute kidney injury (AKI) in severe burns, and identify the risk factors for early AKI and late AKI, respectively.

**Materials and Methods**

A retrospective, descriptive study was performed to 637 adult patients with total body surface area (TBSA) burns of more than 30%, They were admitted to the Institute of Burn Research, Southwest Hospital of Army Medical University of China during last six-year period. The basic characteristics and prognosis were compared, the risk factors of AKI were filtered by multifactor logistic regression analysis.

**Results**

A total of 637 patients were included in analysis, 235 patients were diagnosed AKI, with an incidence of 36.89%,. The mortality of patients with AKI was 34.23%. Multiple logistic regression analysis revealed that age, male sex, TBSA,full-thickness TBSA, pre-existing hypertension or/and diabetes, tracheotomy, hypovolemic shock of early burn were independently associated with both early and late AKI, furthermore, sepsis was only with late AKI. Hypovolemic shock of early burn and sepsis increased the risks of late AKI 8.83 times, 13.76 times, respectively.

**Conclusions**

AKI remains prevalent and is associated with high mortality in severe burn patients. late AKI had a lower occurrence rate, but more severity and worse prognosis.

### Abstract Topic: Nursing Care in Burn Centre

#### S10 Progress in the diagnosis and treatment of ventilator-associated lung injury after burn injury

##### Guanghua Guo, Jinxiu Zhou, Mingzhuo Liu

###### Department of Burns, The first affiliated hospital of Nanchang University, Nanchang, Jiangxi, China

####### **Correspondence:** Guanghua Guo

**Background**

As one of the important means for severely burned patients, mechanical ventilation could not only improve the function of important organs such as heart, lung, and kidney, but also stabilize the homeostasis of body, thus promoting the recovery of patients. Improper use of mechanical ventilation, however, could lead to many complications, among which one of the most common and serious complications is the ventilator-induced lung injury (VILI), accompanying with a high risk of mortality. The target of preventing VILI is to minimize the risk of lung injury caused by mechanical ventilation.

**Materials and Methods**

In this study, 29 articles on ventilator-related lung injury were screened from the databases of CNKI, Pubmed, Wanfang data, and the advanced viewpoints and experimental results were summarized.

**Results**

This present article reviewed the pathogenesis, diagnosis, and early prevention of VILI in burned patients.

**Conclusions**

The formation of VILI is not caused by a single factor, but involves many factors such as physics, chemistry and biology. With the development of the current research on pathogenesis, some new treatment methods, such as biotherapy and gene therapy, have made progress in animal experiments. At present, the treatment and prevention of VILI need to be further studied. In our clinical diagnosis and treatment, it is necessary to monitor the changes of cardio-pulmonary function and condition of the patients at any time, evaluate the general conditions of the patients at any time, and adjust the parameters of mechanical ventilation to minimize the risk of VILI in patients.

### Abstract Topic: Critical Care/Resuscitation and Pharmacology

#### S11 Increased phenotype of bone marrow dendritic cells and inhibition of xenogenesis lymphocyte proliferation in mice with smoke inhalation injury

##### Guanghua Guo, Chunxia Gan, Mingzhuo Liu, Xincheng Liao

###### ^1^Department of Burn, The First Affiliated Hospital of Nanchang University, Nanchang, Jiangxi, China

####### **Correspondence:** Guanghua Guo

**Background**

Inhalation injury increases the mortality of burn patients. The main cause of death is the strong immune response in lung tissue after smoke inhalation. Dendritic cells are the strongest antigen presenting cells so far. There is no research between inhalation injury and dendritic cells. From this point on, we will explore the changes of phenotype and function of antigen presenting cells after inhalation injury

**Materials and Methods**

Material:BALB/c mice, PrimeScript™ RT reagent Kit, TB Green™ Premix Ex Taq™, GM-SCF, IL-4, flow cytometric antibody:CD4, CD8, MHC II, etc. Methods: 1, built the smoke inhalation injury model; 2, extracted lung tissues; 3, cultured the cell; 4, stain the flow cytometric antibodies; 5, analysis data.

**Results**

After injury, the alveolar wall became thicker, a large number of inflammatory cells infiltrated (lymphocyte, macrophage), alveolar fusion and expansion occurred and fibrous tissue proliferation occurred.The level of TNF-α , IFN-γ and IL-4 were increased. The expression of FoxP3 was inhibited after injury. The costimulatory molecule expression of CD80 and MHC-II on the DC is increased. The ability of stimulating allogeneic lymphocyte proliferation was significantly weakened

**Conclusions**

Bone marrow dendritic cells were increased and xenogenesis lymphocyte proliferation were inhibited in mice after smoke inhalation injury

### Abstract Topic: Critical Care/Resuscitation and Pharmacology

#### S12 Early hemodynamic changes in extensive burn patients with effective fluid resuscitation

##### Jian Liu^1^, Yiqing Liu^1^, Shengjun Liu^2^, Jiexin Zheng^1^, Yiwen Niu^1^, Qin Zhang^1^

###### ^1^Burn & Plastic Surgery, Ruijin Hosptial, Shanghai Jiaotong University, School of Medicine, Shanghai, China; ^2^Clinical Medicine, Shanghai Jiaotong University, School of Medicine, Shanghai, China

####### **Correspondence:** Jian Liu

**Background**

To verify early hemodynamic changes following the effective fluid resuscitation in extensive burn patients

**Materials and Methods**

Extensive burn patients with more than 30%TBSA (Total Burn Surface Area), with no comorbities, in-hospitalized within 8 hours post-burn were enrolled into the study. Fluid resuscitation were immediately administered following the protocol of 1.5ml/kg.%TBSA and revised by patients’ clinical manifestation with no prescription of sedative medications and angio-active agents. Hemodynamic criteria, like central venous pressure (CVP), cardiac index(CI), general end diastolic volume index (GEDI), left ventricular contract index(dPmax), systemic vascular resistance index(SVRI) and extravascular lung water index(EVLWI) , were measured by PiCCO measurement in hypovolemic phase to evaluate patients’ hemodynamic changes and pulmonary interstitial edema.

**Results**

27 patients with a mean age of 46.43±10.85 years and a mean TBSA burn of 60.43±8.21%, were studied. Under the resuscitation protocol, it was successful to maintain urine output of 30-60ml/hr indicating effective fluid resuscitation. There was significant cardiac contractility impairment, indicated by left ventricular contract index(dPmax) at the value of 980±234.9 , only 18.3% that of the normal value. And CI were decreased to 2.07±0.28(L/min/m2), 68% of the normal value. It persisted until 48 hours postburn while EVLWI reached its peak at 14.0±0.83 ml/kg. Furthermore, the extravascular lung water was impend to reach a high level of 6.0±0.42 ml/kg soon after the burn even before fluid therapy and it started to slope down after 72 hours postburn spontaneously.

**Conclusions**

There existed the early myocardial contractility impairment soon after the extensive burns with effective fluid resuscitation, which could be a clue for us to pursue preservation of myocardial contractility to restore pump function, minimize pulmonary edema so as to preserve effective tissue oxygenation.

### Abstract Topic: Early Excision and Surgical Techniques

#### S13 The Effect of Tangential Excision and Split Thickness Skin Graft in Reducing Length of Stay in Burns Patients in Jakarta Islamic Hospital Cempaka Putih, Indonesia

##### Gammaditya A Winarno^1^, Aditya Wardhana^2^, Sanjaya F Tanjunga^1^, Amani S Augiani^1^, An’umillah A Zidna^1^

###### ^1^Internship General Practitioner, Jakarta Islamic Hospital Cempaka Putih, Jakarta, Indonesia; ^2^Plastic Surgeon, Jakarta Islamic Hospital Cempaka Putih, Jakarta, Indonesia

####### **Correspondence:** Gammaditya A Winarno

**Background**

Early tangential excision (TE) and split thickness skin graft (STSG) have increased outcome in burn patients treated at specialized burn centers. This study was conducted to compare the length of stay (LOS) in burn patients undergoing early TE & STSG and delayed TE & STSG.

**Materials and Methods**

This is a retrospective cross-sectional study including 42 patients with varied burn degrees and TBSA admitted to Jakarta Islamic Hospital Cempaka Putih (JIHCP) Burn Unit. They were assigned to two study groups, the early TE & STSG group including 32 patients and the delayed TE & STSG group including 10 patients. The data of all patients were collected from medical records and compared between two study groups.

**Results**

Most of the samples were adult male, with average of 13.57±9.57% TBSA grade II burns, caused by flame. The mean of LOS in the group with early TE & STSG was shorter (9.81±6.41 days) than LOS in the delayed TE & STSG group (15.80±5.67 days). The data of LOS between these groups were compared using Independent T-test. The LOS in the early TE & STSG group was significantly shorter than the delayed TE & STSG group (p=0.012).

**Conclusions**

In patients with burn injuries, early TE & STSG is associated with shorter length of stay compared to the delayed TE & STSG. Our study indicates that early excision within 5 days after burn injury is optimal to reduce length of stay in burn patients.

### Abstract Topic: Tissue Engineering

#### S14 Chronic wound animal model

##### Tuan Ngoc Nguyen, Dzung Tien Nguyen

###### Vietnam National Burns Hospital, Military Medical University, Hanoi, Vietnam

####### **Correspondence:** Dzung Tien Nguyen

**Background**

An animal chronic wound is necessary for in vivo research. To today, most animal models of chronic wounds include ischemia, diabetes, pressure and reperfusion. In this study, we creased ischemia reperfusion wound (pressure ulcer) on rat and estimated histology characteristics of these wound.

**Materials and Methods**

We creased 30 ischemia reperfusion wounds on 30 rats. This study was performed at Wound Healing Center, Vietnam Burns Hospital from May to August, 2018. Animal wound model: Rats were anesthetized with Ketamine (80mg/kg IP). Surgical incision was conducted on dorsal skin. A steel plate (φ: 1.5cm) was inserted beneath the dorsal skin through incision. A magnet (φ: 1.5cm) was put on skin and the steel plate. We maintained this situation for 24h. Then we evaluated wound bed and biopsied damage skin for histology test.

**Results**

Average wound size of 30 wounds was 1.766 ± 0.14 cm^2^. The obteined wounds were grey skin necrosis. Their surfaces were more concave than normal skin. The wound histology had some featured images as the cutaneous injury expressed in various degrees. The dermal connective tissue was injuried. In the subcutaneous, looking at the inflammative, fibrosis or/and senescent tissues.

**Conclusions**

The obtained wounds by this model has manifestations of the ischemia reperfusion wound (pressure ulcer). These wounds can apply for in vivo researchs.

### Abstract Topic: Early Excision and Surgical Techniques

#### S15 Early excision and skin grafting of extensive deep burns

##### Guodong Song^1^, Donghui Bian^2^

###### ^1^Department of Burns and Plastic Surgery, First Aid Center for Burns in Jinan, Jinan Central Hospital Affiliated to Shandong University, Jinan, Shandong, PR China; ^2^Department of Burns and Plastic Surgery, The 960th hospital of the PLA joint logistics support force, Jinan, Shandong, PR China

####### **Correspondence:** Guodong Song

**Background**

Early excision and skin grafting is a key technology of improving the survival and consequences of patients with extensive deep burns (EDB). However, the improvement of overall survival rate of EDB in the world has been not remarkable over the past 20 years. This article aims to discuss the several key issues in early excision and skin grafting of EDB.

**Materials and Methods**

Based on the representative monograph at home and abroad, reviewing relevant literature and combining with our practices.

**Results**

The accurate assessment about burn wound depth and the profound understanding on wound development process are the pathophysiological basis of the wound management. The appropriate timing and area of the wound excision should be chosen with the concept of damage control operation. Tangential excision is applicable to not only deep dermal but also full-thickness burn wounds. The depth of tangential excision should effectively remove necrotic tissues and reach down to the level of the underlying viable dermis or subcutaneous tissue. Tourniquet can be not used during limb tangential excision surgery, instead, the wounded limb has been kept elevated as much as possible. If fresh wound after tangential excision is still obviously dropsical, multipoint puncture reaching down to fascia should be performed for drainage. The wound after tangential excision within 14 days especially 7 days post burn should be grafted with viable alloskin or xenoskin for temporary coverage and later on autoskin. Grafting of autologous microskin overlain with sheet alloskin should be applied onto the wound after tangential excision, of which the blood supply tend to be improved after 7 days post burn. Perioperative especially intraoperative management should be strengthened.

**Conclusions**

Although the treatment strategy on early excision and skin grafting of EDB has obtained the good curative effect, it needs still to be improved and verified.

### Abstract Topic: Early Excision and Surgical Techniques

#### S16 Clinical evaluation and early management of hand deep burns

##### Guodong Song^1^, Donghui Bian^2^

###### ^1^Department of Burns and Plastic Surgery, First Aid Center for Burns in Jinan, Jinan Central Hospital Affiliated to Shandong University, Jinan, Shandong, PR China; ^2^Department of Burns and Plastic Surgery, The 960th hospital of the PLA joint logistics support force, Jinan, Shandong, PR China

####### **Correspondence:** Guodong Song

**Background**

Hand deep burns (HDB) is still one of the main reasons for resulting in physical disabilities of patients with burns, especially serious burns. Early accurate treatment of HDB is the foundation for the best recovery of hand function. This article aims to summarize experience of clinical evaluation and early management of HDB.

**Materials and Methods**

Based on evidence-based medicine principles, reviewing relevant literature and combining with our practices.

**Results**

Dorsal HDB is easy to damage deep tissue, especially central slip of extensor expansion. Palmar hand burns is generally not too deep. And necrosis of finger ends is clinically common. In patients with typical constrictive eschar on upper limb or signs of poor perfusion, an escharotomy should be performed as soon as possible. For patients whose perfusion is not still restored, those with fourth-degree burns, or signs of osteofascial compartment syndrome, a fasciotomy should be performed in time. For dosal hand deep dermal burns or full-thickness burns, tangential excision and medium thickness sheet auto-skin grafting should be performed as early as possible, which should focus on repair of opisthenar and dorsal finger and design reasonable surgical range. For palmar burns, it is necessary to wait 2–3 weeks for healing. If not, full-thickness or thick sheet auto-skin grafting should be performed. Before and after surgery, HDB should be treated by dressing change with local anti-infection, proper compression and elevation. And desiccation of finger ends should be avoided. During upper limb tangential excision surgery, tourniquet cannot be applied to limb. Hand joints should be kept in the position of confrontation contracture. Once the wound is closed, systemic rehabilitation plan should be implemented.

**Conclusions**

Accurate assessment of hand blood supply and timely decompression treatment can effectively prevent secondary injury of HDB. Early excision and skin (flap) grafting and systemic rehabilitation can maximally restore hand function.

### Abstract Topic: Early Excision and Surgical Techniques

#### S17 Development and clinical application of a new foam dressing for negative pressure wound therapy

##### Jiake Chai, Huinan Yin

###### Burn Institute, Fourth Medical Center of PLA General Hospital, Beijing, China

####### **Correspondence:** Jiake Chai

**Background**

At present, the widely use of negative pressure wound therapy (NPWT) has achieved remarkable therapeutic effects. However, some defects were found during its clinical use. Because the foam dressing is in the form of a block and inflexible, when it is used for large wounds such as entire limbs or special parts of the body such as hands, it is time-consuming and laborious, et al which affects its use and treatment effects. In this study, a new foam dressing for entire limbs and hands was introduced.

**Materials and Methods**

The new foam dressing with was thinner and much longer than the previous foam dressing and had a bandage-like appearance. It can be wrapped around the entire limbs and hands like a bandage. The drape for closing the limb wounds was much larger than the previous one. One or two pieces can seal the entire limbs (usually one piece for upper limb and two pieces for lower limb). A non-perforated drainage tube was used.

**Results**

When the new dressing was applied to the entire lower limbs, one staff could complete the operation within 10 minutes, while the previous foam dressing required 2-3 staffs to complete at least half an hour. Because of the reduction of the number of drainage tubes and the reduction of film splicing, the incidence of air leakage was reduced, and the wound sealing effect was better. The new dressing had lower pressure on limb than the conventional dressing and would not cause ischemia of the extremities. Clinical application showed that both the new dressing and the previous foam dressing had good treatment effects on the entire limb wounds and wounds of hands.

**Conclusions**

The new foam dressing for NPWT is safe, convenient to operate, time-saving, labor-saving, and has good sealing effect for entire limbs and hands.

### Abstract Topic: Early Excision and Surgical Techniques

#### S18 Use of Glycerol Preserved Allografts (GPA) in acute burn management

##### Sunil Keswani

###### National Burns Centre, Navi Mumbai, India

**Background**

Major burns which are deep second/third degree are not likely to heal spontaneously and invariably require resurfacing. If the wounds are not contaminated and patient has adequate donor area, autografts are used. If the wounds are contaminated after excision, initially allografts are applied which help prepare the wound for definitive resurfacing with autografts. If the patient does not have adequate donor sites, then autografts are harvested from the limited area and meshed widely and then covered with allografts (meshed 1:1 or 2:1), as a sandwich technique. The allografts help by providing temporary cover facilitating autografts to take easily.

**Materials and Methods**

Patients with deep burns were taken up for early excision and resurfaced with autografts alone or autografts (widely meshed) covered by allografts (sparingly meshed). The results were analyzed in terms quality of healing and presence or absence of contractures. In the event, the allografts were rejected, they were removed and fresh allografts were applied. In the wounds which were referred to the hospital in an infected condition excision was done and only allografts were applied to be replaced by autografts once wound condition improved and the wound was ready for grafting.

**Results**

It was observed that the use of GPA was associated with good healing of the wounds with lesser incidence of graft loss and better quality of healing, compared with patients where allografts had not been used.

**Conclusions** Allografts have now come to stay and are a useful adjunct in the burn surgeon’s armamentarium in the resurfacing of major burn wounds.

### Abstract Topic: Infection Control and Diseases

#### S19 Are we doing enough for burns? Doctors in Dhaka have a say

##### Low Tzuemn Ling^1^, Tanveer Ahmed Himon^2^, Chong Xin Ying^3^, Foong Shyn Yih Hayley^3^, Chong Si Jack^3^

###### ^1^Ministry of Health Holdings, Singapore; ^2^Department of Plastic Surgery and Burn Unit, Dhaka Medical College and Hospital, Dhaka, Bangladesh; ^3^Asia Pacific Burn Association, Bali, Indonesia

####### **Correspondence:** Low Tzuemn Ling

**Background**

Burns are a global public health problem, accounting for an estimated 180 000 deaths every year, according to WHO. The vast majority occur in low and middle-income countries. Non-fatal burns often require lengthy and gruesome hospitalisations, where multiple treatments are administered, early rehabilitation and life-long follow up. The impact of burns on burns victims and the society can never be overstated. Many factors, beginning from the cause of burns, affects the final burns outcome. This study aims to evaluate the epidemiology of burns in Dhaka.

**Materials and Methods**

A survey form was employed to standardise the interview process. Survey form was prepared in English. Doctors were approached randomly at the local Burns unit at Dhaka, while they were at work. All interviews were conducted, one-to-one, with the assistance of a translator and with the doctors’ verbal consent.

**Results**

15 doctors participated in the study. 60% of them were males while the rest were females. 80% (12) chose English to be the language medium of choice whereas 13.3% (2) was other languages and 6.7% (1) was Bengali. The two most common burn injuries were thermal (15) and electrical burns (15). 100% agreed that thermal burns were more prevalent than electrical burns. Other types of burns reported were chemical (9), friction (7), radiation (2), lightning (1) and abuse (1). 100% of them agreed that there were not enough safety precautions in place to prevent electrical burns. 80% of them thought that education was lacking in the community. Other popular causes reported were inadequate general public awareness (73%, 11), overcrowding in poor housing conditions (67%, 10), poor education levels (53%, 8) and poor city/home layout/hazard placements (53%, 8). 11 (73.3%) thought that doctors, instead of nurses should lead Burns Resuscitation and 9 (60%) thought that doctors, instead of nurses should lead Burns Dressings. According to this group of local doctors, the complication of Burn Injuries which patients complain the most of was disfigurement.

**Conclusions**

The high prevalence of burns, undeniably is attributed to anthropogenic reasons. Thus, burns are preventable, excluding that caused by natural disasters for example in this study, lightning. The consensus among these Dhaka doctors is that much effort is still needed to help the burns community. While the Dhaka authorities formulate their policies and plan their city, the authors recognise that a new tertiary level hospital is on the way in Dhaka.

### Abstract Topic: Infection Control and Diseases

#### S20 Lytic Bacteriophage Screening Strategies against Multidrug Resistant Bloodstream Infections from an Intensive Care Unit of Southwest China Burn Treatment Center

##### Zichen Yang, Liuyang Deng, Supeng Yin, Bei Jiang, Yulong Zhang, Bo You, Cheng Zhang, Xiaoqiang Luo, Yizhi Peng, Yali Gong

###### State Key Laboratory of Trauma, Burns and Combined Injury, Chongqing Key Laboratory for Proteomics Disease, Institute of Burn Research, Southwest Hospital, the Third Military Medical University (Army Medical University), Chongqing 400038, China

####### **Correspondence:** Yali Gong

**Background**

Bloodstream infection (BSI) is the dominant cause of death in severely burned patients. With increasing antibiotic resistance, more attentions should be paid. In this study, we collected and analyzed the pathogen distribution and drug resistance in 486 Burn Intensive Care Unit patients with bloodstream infections (BSI) that were admitted into Burn Intensive Care Unit (BICU) of our hospital for seven years.

**Materials and Methods**

This retrospective observation study was conducted at the BICU of Southwest Hospital from January 2011 to December 2017. The study subjects were all the severely burned patients diagnosed with BSI, whose blood samples was taken and cultured at least once. The antibiotic susceptibility tests were performed.

**Results**

The patients with extreme more severe burns had significantly more kinds of bloodstream infection. Gram-negative bacilli were the main causative agents of BSI in severely burned patients (64.6%), followed by Gram-negative cocci (27.7%) and fungi (7.7%). Of all the pathogens, the top three species in terms of the detection rate were A. baumannii (26.0%), S. aureus (16.8%) and P. aeruginosa (14.2%) with multidrug resistance (MDR) respectively.

**Conclusions**

In conclusion, we carried out an etiological characterization of the 486 severely burned patients with bloodstream infections admitted into BICU. Gram-negative (MDR) A. baumannii and P. aeruginosa and Gram-positive drug-resistant S. aureus were the most important agents of BSI in severely burned patients, when the infection and drug resistance of K. pneumoniae and E. cloacae became newly emerged threats. The etiologic agents of BSIs in our BICU differed from those previously reported studies suggesting a need in routine bacteria monitoring.

### Abstract Topic: Infection Control and Diseases

#### S21 CRISPR-dCas9 mediated control of Pseudomonas aeruginosa infections

##### Guangtao Huang, Zairong Wei, Dali Wang

###### Department of Burn and Plastic, Zunyi Medical University, Zunyi,Guizhou, China

####### **Correspondence:** Guangtao Huang

**Background**

P. aeruginosa is a widely distributed non-fermentative Gram-negative bacteria. It is also an important leading opportunistic pathogen, causing nosocomial infection. Gene interference is a necessary strategy to investigate the essential genes in P.aeruginosa.

**Materials and Methods**

To set up a gene interference platform in P. aeruginosa, CRISPR system was used with a deactive Cas9 protein. CRISPR-dCas9 system was cloned into an inducible shuttle vector pHERD20T.

**Results**

When an essential P. aeruginosa gene, prtR, was targeted by CRISPR-dCas9 system, a clear growth defect of the P. aeruginosa was observed.

**Conclusions**

CRISPR-dCas9 based gene knockdown system has successfully been built in P. aeruginosa, which can be used as a powerful tool in the investigation of essential or difficult-to-knockout genes of P. aeruginosa

### Abstract Topic: Infection Control and Diseases

#### S22 Characteristics of fungal infections in severe burn patients

##### Minh Nguyen Thai Ngoc^1^, Lam Nguyen Nhu^1^, Anh Tran^2^

###### ^1^Viet Nam National Burns Hospital, Military Medical University, Ha Noi, Viet Nam; ^2^Viet Nam Military Medical University, Ha Noi, Viet Nam

####### **Correspondence:** Minh Nguyen Thai Ngoc

**Background**

Invasive fungal infection is a challenge, a burden for the health sector due to high costs and high mortality rates. Severe burn patients treated in ICU departments are in the group at high risk of invasive fungal infection.

**Materials and Methods**

A prospective study was carried in Intensive care unit of Viet Nam National burns hospital from 1/2017 to 6/2108. Patients had at least one blood culture test and / or biopsy tissue positive with fungal, which is the same type of fungal isolates in patient samples at other non-sterile sites on the body. We diagnosed invasive fungal infections in severe burn patients based on evidence of microorganisms and monitor assessments of clinical and subclinical symptoms of patients.

**Results**

30 patients with invasive fungal infection were recorded in our study. Total burn surface area was 52.8 ± 19.46%, deep burn was 33.4 ± 16.17%. The Apache II score at admission was 14 ± 3.81 and the average time to diagnose invasive fungal infection was 21.6 ± 8.62 days. In the study, we found 2 species of fungal pathogens: *Candida* and *Aspergillus*. The proportion of *Candida non-albicans* species in the study found 15 patients infected with *Candida tropicalis* (56%).

**Conclusions**

Severe burn patients are at risk of invasive fungal infection from the second week after admission. Clinical symptoms are not specific but suggestive of diagnosis and correspond to subclinical microbiological results.

### Abstract Topic: Infection Control and Diseases

#### S23 Identification of Candidate Biomarkers correlated with diabetic foot ulcer with Bioinformatics analysis

##### Hongyan Zhang, Peng Wang

###### Department of burn ,The First Affiliated Hospital of Nanchang University, China

####### **Correspondence:** Hongyan Zhang

**Background**

Diabetic foot ulcers (DFUs) are one of the major complications in diabetes patients and can result in a high rate of amputation and mortality Its molecular pathology remains barely understood, impeding the development of effective treatments.

**Materials and Methods**

We searched the Gene Expression Omnibus database (GEO, http://www.ncbi.nlm.nih.gov/geo/) for studies on DFUs, using the keywords “Diabetic foot ulcers.” microarray data was submitted to the Limma package of R software to the differential expression genes between diabetic foot ulcer and non-diabetic foot skin. Utilizing David Bioinformatics software, the selected differential genes were utilized to annotate the GO terms and pathways. Biological Chart visualization tool Cytoscape software is utilized to construct the protein Interaction Network (PPI), miRNA-DEGs, lnRNA-DEGs regulatory network.

**Results**

Three mRNA expression profiles (GSE80178, GSE84971, GSE68185) were issued by the GEO database. A total of 29 overlapped unregulated genes and 37 down regulated genes were identified. The majority of the DEGs were focused on apoptosis and the process of positive regulation of apoptosis. In the course of biology, the highest enrichment is in the process of cell cycle change. The results of the comprehensive PPI, miRNA-DEGs and lnRNA-DEGs control network, UBC, HSPA8 in the PPI network in the core node position, hsa-miR-185-5p, hsa-miR-4516s in an important position in the miRNA-DEGs regulatory Network diagram.NR-029894, NR-029410 are in the strategic position of LnRNA-DEGs control Network diagram.

**Conclusions**

These data demonstrated UBC and HSPA8 are important markers of the evolution of DFUs. This can gain a better understanding of the molecular mechanisms and provide potentially valuable genes for prevention, diagnosis and treatment therapy.

### Abstract Topic: Infection Control and Diseases

#### S24 A novel functionalized graphene nanocomposite with dual-targeting ability for chemo-photothermal ablation of multidrug-resistant Gram-negative bacterial infection of skin wound

##### He Wang, Wei Qian, Gaoxing Luo

###### Institute of Burn Research, Southwest Hospital, Army Medical University, Chongqing, China

####### **Correspondence:** Wei Qian

**Background**

Multidrug-resistant (MDR) Gram-negative bacterial infections of skin wounds have been a major challenge in the treatment of burns and trauma. As one of the alternative strategies, PTT has recently become a research hotspot. However, the greatest defect for PTT could be the difficulty of targeting for the pathogenic bacteria to avoid the undesirable damage of ambient tissue. Furthermore, monotherapy may also be inefficient. To address these challenges, a novel graphene based nanocomposite with efficient dual-targeting ability for chemo-photothermal synergistic therapy was synthesized.

**Materials and Methods**

Aminobenzeneboronic acid (B) was grafted onto carboxyl graphene (CG) by the condensation reaction of amine groups on B and carboxyl groups on CG. The resultant B-CG composite was then quaternized to form B-CG-QAS nanocomposite. The characterization of B-CG-QAS was performed by FTIR, TEM, AFM, etc. The *in vitro* and *in vivo* toxicity was evaluated on the 3T3 fibroblast and mice, respectively. The MDR A. baumannii, K. pneumoniae and P. aeruginosa were chosen as the testing bacteria. The targeting abilities for bacteria and biofilm were explored by the methods of SEM observation, fluorescent staining and thermal imaging. The *in vitro* antibacterial and anti-biofilm efficiencies were determined using the standard plate count method. The *in vivo* antibacterial and anti-biofilm efficiencies were investigated in the murine skin wound infection model and subcutaneous abscess model, respectively.

**Results**

Due to stable covalent bonding and electrostatic attraction between B-CG-QAS and bacterial cell or biofilm matrix, B-CG-QAS exhibits a very strong targeting ability for the bacteria and biofilms. *In vitro*, B-G-QAS mediated chemo-photothermal therapy can effectively kill the MDR Gram-negative bacteria and biofilms; *in vivo,* it can also ablate the bacterial and biofilm infection, significantly accelerating wound healing. B-G-QAS shows excellent biocompatibility *in vitro* and *in vivo*.

**Conclusions**

The B-CG-QAS described here, provides a novel treatment strategy for MDR Gram-negative bacterial and biofilm infection of skin wound.

### Abstract Topic: Infection Control and Diseases

#### S25 Clinical Effect of Hydrodynamic Debridement on Severe Infectious Wounds

##### Zongyu Li, Zhuolyu

###### Department of Burns, the Fifth Hospital of Harbin, Harbin, China

####### **Correspondence:** Zongyu Li

**Background**

Invasive refractory infection is one of the common complications of wounds by various pathogens. This kind wound easily induces systemic infection, even sepsis. The most important treatment for the invasive refractory infectious wound is to remove the local pathogens effectively and immediately. It is difficult to delete the pathogens and devitalized tissues precisely by the conventional methods. Hydrodynamic debridement was reported suitable to treat different wounds for its accuracy and convenience. In this study we intend to observe the clinical effects of hydrodynamic debridement on refractory infectious wounds.

**Materials and Methods**

From 2014 to 2018, there were 32 cases with refractory infectious wounds involved in this study, in which 28 cases were induced by burn injury. All the wounds were treated with a hydrodynamic debridement system versajet-II. The clinical effects were observed and recorded after the treatment.

**Results**

After the hydrodynamic debridement, 32 patients were immediately underwent skin grafting or suture directly. More than 90% wound healed well. Moreover, the induced systemic infections were effectively controlled. There was no patient died in this study.

**Conclusions**

The hydrodynamic debridement system is one of the effective clinical methods to treat refractory infectious wounds.

### Abstract Topic: Infection Control and Diseases

#### S26 Effects of pulmonary protective ventilation strategy combined with lung recruitment on hemodynamics in patients with severe burn complicated with ARDS in different sedation modes

##### Tian Xinli,Geng Kang,Zhou Weiwei,Yan Hong

###### Department of Plastic and Burn surgery, the Affiliated Hospital of Southwest Medical University, Luzhou, Sichuan, 646000, P.R.China

####### **Correspondence:** Tian Xinli

**Background**

The effect of pulmonary protective ventilation strategy combined with lung recruitment on hemodynamics in patients with severe burns complicated with ARDS with propofol, midazolam and dexmedetomidine sedation.

**Materials and Methods**

Thirty patients with severe burns complicated with ARDS were randomly divided into three groups. They were sedated with propofol, midazolam and dexmedetomidine. Mechanical ventilation was also performed in the pulmonary protective ventilation strategy. The time taken to reach the target sedation depth, dynamic monitoring of hemodynamic parameters heart rate, mean arterial pressure (MAP), central venous pressure (CVP) and cardiac output (CO), extravascular lung water index (EVLWI) . The blood gas analyzer was used to determine the blood gas analysis index PH value, PaO2, PaCO2, and the OI was calculated. The treatment status and results of the patients were counted. Repeated measures analysis of variance and LSD test for data row single data

**Results**

(1) The MAP and HR variability of dexmedepine was lower than that of propofol and midazolam. The difference was statistically significant (P<0.01). There was no significant change in heart rate, MAP, CVP and CO. (P>0.5). 2) Before the combination of lung recruitment, lung recruitment and lung re-expansion, the changes of PaO2, EVLWI and OI in this group were significant (P<0.01), PH value and PaCO2, no significant change (P>0.05). (3) All patients were cured without other complications

**Conclusions**

1) For patients with burn complicated with ARDS, sedative treatment with dexmedetomidine hydrochloride has less effect on hemodynamics than midazolam and propofol; 2) pulmonary protective ventilation strategy combined with pulmonary complexion Zhang can significantly improve the oxygenation of patients with severe burns complicated with ARDS, and may improve the prognosis of patients

### Abstract Topic: Infection Control and Diseases

#### S27 Treatment of carbapenem-resistant Klebsieila pneumoniae infection in burns

##### Jingning Huan

###### Department of Burn & Plastic Surgery, Ruijin Hospital, Shanghai Jiaotong University School of Medicine, Shanghai 20025,PR.China

**Background**

The emergence of carbapenem resistant Klebsieila pneumoniae (CRKP )is an important threat to burned patients.

**Materials and Methods**

This retrospective study was conducted to collect information from burned patients in Shanghai Ruijin Hospital between 2010 and 2017.

**Results**

A significant increase in number of CRKP isolated from burned patients was observed between 2010 and 2017.CRKP were resistant to almost all available antimicrobials and are susceptible only to polymyxins, tigecycline. A minority of CRKP are also susceptible to the few remaining aminoglycosides. Antibiotics which can been currently used to treat CRKP infections include polymyxins, tigecycline and some type of aminoglycosides.

**Conclusions**

The efficacy of combination antibiotics therapy is better than single-agent therapy for the treatment of CRKP bloodstream infections. In order to control the spread of CRKP, strategy for preventing CRKP dissemination in hospitals were suggested.

### Abstract Topic: Infection Control and Diseases

#### S28 Development of an isothermal recombinase polymerase amplification assay for rapid and accurate detection of *Acinetobacter baumannii* and carbapenem resistance gene

##### Shuang Liu^1^,Guangtao Huang^2^,Yali Gong^2^,Yudan Meng^1^,Yizhi Peng^2^,Junning Zhao^3^,Li yang^4^,Xiaolu Li^1,3^

###### ^1^The Affiliated Hospital of Southwest Medical University, the department of Plastic & Burns Surgery, Tai Ping Street, Luzhou 646000, People's Republic of China; ^2^Institute of Burn Research, Southwest Hospital, The Third Military Medical University, Gao Tan Yan Zheng Street ,Chongqing400038, People's Republic of China; ^3^Sichuan Academy of Chinese Medical Sciences, Sichuan Translational Medicine Center of Chinese Medicine, Ren Min Nan Lu Road, Chengdu 610041, People's Republic of China; ^4^State Key Laboratory of Biotherapy and Cancer Center, West China Hospital, Sichuan University and Collaborative Innovation Center, Ren Min Nan Lu Road ,Chengdu 610041, People's Republic of China.

####### **Correspondence:** Shuang Liu; Xiaolu Li

**Background**

*Acinetobacter baumannii(A.baumannii)* is a leading pathogen causing hospital-acquired infections, and the drug-resistance rate of *A. baumannii* has been increased continuously in recent years. In this study, we developed a new 15ul reaction system of recombinase polymerase amplification (RPA) for rapid detection of *A. baumannii* and carbapenem resistance gene.

**Materials and Methods**

We collected 30 *A. baumannii* clinical isolates from Burn Institute of Army Medical University though 6 months and tested the antibiotic susceptibility by Kirby-Bauer method. *A. baumannii* was detected using *bla*_OXA-51_ gene by PCR, qPCR and 15ul-RPA respectively. We evaluated the sensitivity, specificity and the detection rate of clinical isolates. In addition, we detected of *bla*_OXA-23_ resistance gene by 15ul-RPA.

**Results**

Up to 90% of these strains were resistant to meropenem and imipenem. The 15ul-RPA detected successfully *A. baumannii* within18min at 42°C, the detection limit of *bla*_OXA-51_ gene was 2.86CFU/ml, which had same sensitivity as PCR, and qPCR. The specificity of two identification genes was also extremely high, no positive amplification signal in non-*Acinetobacter* was observed. The detection rate of *A. baumannii* clinical isolates using *bla*_OXA-51_ gene was 100%. Furthermore, we tested the *bla*_OXA-23_ resistance gene of *A. baumannii* by 15ul-RPA, the result showed majority (90%) of clinical isolates examined carrying the *bla*_OXA-23_ gene. Compared with the results of the antibiotic susceptibility test, we found that all carbapenem-resistant *A.baumannii* isolates carrying the *bla*_OXA-23_ gene, while no the *bla*_OXA-23_ gene was detected in carbapenem-sensitive *A.baumannii*.

**Conclusions**

15ul-RPA analysis for detection *A. baumannii* and *bla*_OXA-23_ gene was faster and simpler than qPCR, PCR, and cheaper than conventional 50ul-RPA. We consider that 15ul-RPA has potential as promising alternative molecular diagnostic method to rapid and effective detection of *A. baumannii* and drug resistance gene in the Point-of-Care Testing.

### Abstract Topic: Infection Control and Diseases

#### S29 Quantitative analysis of bacterial load in burn patients treated with topical application of silver sulphadiazine cream and nanocrystalline silver hydrogel

##### Sanjeev Kumar Uppal^1^, Avinash Gupta^1^, Dipan Uppal^2^, Deepinder Kaur Chhina^1^, Rajinder K Mittal^1^

###### ^1^Dayanand Medical College, Ludhiana , Punjab , India; ^2^Maulana Azad Medical College, Delhi , India

####### **Correspondence:** Sanjeev Kumar Uppal

**Background**

The quantitative cultures are more valuable in prediction of metastatic invasion of organisms. It gives the critical bacterial load (10 ^5^ CFU/gram of tissue) beyond which metastatic invasion is possible. The effect of conventional silver sulphadiazine dressing with colloidal nanosilver hydrogel dressing in burn wounds needs to be compared based on quatitative cultures

**Materials and Methods**

60 patients of burns upto 60 % were included and divided into silver sulphadiazine and nanosilver hydrogel groups. Wound cultures for qualitative and quantitative analyses were taken at the end of first week, second , third and fourth week.

**Results**

In Nanocrystalline Silver group average bacterial load of Klebsiella progressively decreased Average bacterial load of E.coli also progressively decreased . There was progressive increase in average bacterial load of Pseudomonas during second and third weeks . The increase continued in the fourth week

**Conclusions**

Nanosilver hydrogel significantly reduces the bacterial load in burn wounds as compared to silver sulphadiazine. There is complete clearance of E coli in nanosilver hydrogel group during our study period. The effect of nano silver hydrogel was not significant in Pseudomonas.

### Abstract Topic: Nursing Care in Burn Centre

#### S30 Devastating Burns: Perspectives of Burns patients at Dhaka Medical College Hospital

##### Low Tzuemn Ling^1^, Tanveer Ahmed Himon^2^, Chong Si Jack^3^, Chong Xin Ying^3^, Foong Shyn Yih Hayley^3^

###### ^1^Ministry of Health Holdings, Singapore; ^2^Department of Plastic Surgery and Burn Unit, Dhaka Medical College and Hospital, Dhaka, Bangladesh; ^3^Asia Pacific Burn Association, Bali Singapore

####### **Correspondence:** Low Tzuemn Ling

**Background**

We acknowledge that it is impossible for anyone to understand the pain of these burn victims, let alone begin to imagine how devastating it must have been and still is for them. We endeavoured to explore the experiences of burn patients. In doing so, we hope to gain more insight, strive to alleviate their suffering and provide comfort.

**Materials and Methods**

Interviews were conducted in both male and female burns wards at Dhaka Medical College Hospital. Verbal consent was taken with the assistance of a translator. Sleeping, paediatric and critical care patients were excluded. Patients, who were unable to speak for various reasons, were also excluded. This study was carried out with permission from the Dhaka Medical College Hospital.

**Results**

A total of 36 patients were interviewed. 20 (55.6%) were male and 16 (44.4%) were female. All 16 females were housewives. Most of their burn injuries were caused by poor city layout, home layout and hazardous placements (17, 47.2%) and lack of safety installations in the form of wire insulations, faulty electrical circuits (15, 41.7%). 3 (8.3%) of the patients thought that the community was too overcrowded. 63.9% were thermal injuries, 33.3% were electrical injuries and the remaining were chemical injuries (2.8%). All of them spoke Bengali, only 1 was able to speak both Bengali and English. He used to work in Singapore as a construction worker. 6 (16.7%) of them were uneducated. Majority of them 19 (52.8%) were of primary school qualifications.

**Conclusions**

According to WHO, the vast majority of burns worldwide occur in low- and middle-income countries. Within all countries, burn risk correlates with socioeconomic status. Nearly 173000 Bangladeshi children are moderately or severely burnt every year. The effects of burns are far-reaching and reasonably more damaging for children than adults. However, this study is limited to adults and future study can be considered to include children for a more wholesome picture.

### Abstract Topic: Nursing Care in Burn Centre

#### S31 The Experience of Compassion Fatigue after Caring of Major Burned Patient among Nurses in the Burn Center

##### Feng-Mei Tseng^1^, Ching-Huey Chen^2^

###### ^1^Burn center, National Cheng Kung University Hospital, Tainan City, Taiwan; ^2^PhD, RN, Professor, Department of Nursing, Chang Jung Christian University, Tainan City, Taiwan

####### **Correspondence:** Feng-Mei Tseng

**Background**

The purpose of this study was to understand the experience of compassion fatigue after caring of major burned patient among nurses in the burn center.

**Materials and Methods**

Using qualitative research, nine burn center nurses were selected at a medical center in southern Taiwan to collect data with interviews, observations, and reflective records.

**Results**

The period of research is from January 1, 2018 to June 30, 2018. The findings are as follows: (1) " Empathy" is a key element in the continuation of nursing and a factor that causes compassion fatigue. (2) Symptoms of compassion fatigues (such as physical and mental fatigue, anxiety, etc.) will affect the nurses, showing a lack of sense of accomplishment, want to escape and other characteristics; if not properly resolved, will lead to personal trapped in negative emotions.(3) The caregiver is afraid of family concerns and is solely responsible for the fatigue that is not seen. (4) The mutual support of the burn team members has become a major factor in reducing compassion fatigues.

**Conclusions**

Under the pressure of high compassion fatigues, it will affect the motivation of continuous service in the burn center. Therefore, the supervisor should pay attention to and care for the nurses, continue to provide psychological support, and infuse positive energy. The nurses also need to identify themselves. Support each other then will coexist.

### Abstract Topic: Nursing Care in Burn Centre

#### S32 Project to Improving the Nurses Working Overtime----A Medical Center Burn ICU in Taiwan

##### Chen-Chun Chen, Meng-Jung Chuang, Ling-Yin Chiu, Feng-Mei Tseng

###### Nursing Department, National Cheng Kung University Hospital , Tainan, Taiwan

####### **Correspondence:** Chen-Chun Chen

**Background**

This is a burn intensive care unit, which mainly cares for severely burn patients and the other critical patients. When the registered professional nurses seriously work overtime, they will directly affect their physical and mental health, which will lower the care quality and influence the safety of the patients. This project is investigating overtime to offer improve strategies to reduce working overtime.

**Materials and Methods**

This project is from November, 2017 to April, 2018, through Plato and fishbone diagram analysis mainly reason of work overtime and get on countermeasures, including: (a) revising the content of the shift specification (b) adding the auxiliary shift card (c) adding the auxiliary shift card holders(d) using the electronic shift system to streamline the shifts(e) adjusting the medical supplies count to simplify the procedure(f) rearranging the auxiliary responsibilities of manpower and work content.

**Results**

The average working hour overtime was reduced from75 to 46.25 points, to reach the project's goals.

**Conclusions**

We hope the project's results can be to promote mental and physical balance, enhance patient safety and quality of care.

### Abstract Topic: Nursing Care in Burn Centre

#### S33 Exploring the Therapeutic Intervention Scoring System-28 (TISS-28) Established by Clinical Information System- A Medical Center Burn ICU in Taiwan

##### Ling-Yin Chiu, Meng-Jung Chuang, Li-Chuan Hung

###### Nursing Department, National Cheng Kung University Hospital, Tainan, Taiwan

####### **Correspondence:** Ling-Yin Chiu

**Background**

There are daily frequent nursing activities and huge physiological signals in the burn intensive care unit. If these signals could be converted into useful information, it will be helpful for clinical care. Therefore, the patient severity classification (such as TISS-28, APACHE II) is expected by automatically collecting data, and the error of manually collecting data is reduced, that more accurate data as a reference for assessing of nursing manpower.

**Materials and Methods**

This Study was from June, 2015 to May, 2016, planning the TISS-28 based form established by clinical information system, through linking existing medical information system in the first phase. After confirming the correctness of the data, the relevant reports were created in the second phase, and the data was analyzed with SPSS 17.0, to explore the correlation between TISS-28 score and nursing workload.

**Results**

The results showed that the TISS-28 based form of automatic capture and calculation was established. The TISS-28 score was positive correlation with nursing workload.

**Conclusions**

The TISS-28 established by clinical information system provides the information of nursing workload, as evidence-based resources for manpower policy.

### Abstract Topic: Nursing Care in Burn Centre

#### S34 Nurse-controlled analgesia with fentanyl for burn dressing changes

##### Thach Ngoc Nguyen^1^, Quynh Van Nguyen^1^, Thang Quoc Tran^2^

###### ^1^Department of Anesthesia, Vietnam National Burns Hospital, Military Medical University, Hanoi, Vietnam; ^2^Intensive Care Unit, Hospital 354, Hanoi, Vietnam

####### **Correspondence:** Thach Ngoc Nguyen

**Background**

Analgesia during burn dressing change is indispensable. Fentanyl has great analgesic potency and short onset of action. Fentanyl has been commonly used for analgesia during burn dressing changes in the United States. Today, the most common analgesic method is patient-controlled analgesia, however, nurse-controlled analgesia method is also used and differences between the two methods in their analgesic efficacy and side effects were statistically insignificant. Up to now, however, in Viet Nam, there hasn’t been any report of nurse-controlled analgesia with fentanyl for burn dressing changes. Therefore, we conducted this study with the aim of evaluating the effectiveness and safety of nurse-controlled analgesia with fentanyl for burn dressing changes.

**Materials and Methods**

Thirty two adult burn cases were given nurse-controlled analgesia with the initial intravenous injection dose of fentanyl 1mcg/kg and the additional intravenous injection doses of fentanyl 30mcg/time whenever their Wong-Baker pain score was ≥ 3 during dressing changes at Intensive Care Unit, National Institute of Burns in Vietnam from July 2017 to February 2018.

**Results**

The number of additional fentanyl intravenous injection times was 2.1 ± 1.16 and the total amount of fentanyl used for analgesia during burn dressing changes was 124.7 ± 34.92mcg. The smallest and the biggest of Wong-Baker average pain scores after fentanyl intravenous injection were 0.3 ± 0.46 and 2.9 ± 0.35, respectively. Satisfied nurse rate was 96.875%. The rate of dizziness, nausea and vomiting, pruritus, sedation with OAA/S4 was 9.3%, 3.1%, 3.1%, 3.1%, respectively.

**Conclusions**

Nurse-controlled analgesia with fentanyl was effective and safe for burn dressing changes

### Abstract Topic: Nursing Care in Burn Centre

#### S35 Efficacy of ultrasound-guided implantation of PICC on children with severe burns

##### Yan Ma, Nan zhang, Duo Cai, Weiwei Wu

###### Bruns Department, the First Hospital of Jilin University, XiminZhu Street No 1409, Changchun, China

####### **Correspondence:** Weiwei Wu

**Background**

To investigate the clinical efficacy of ultrasound-guided implantation of PICC in children with severe burns.

**Materials and Methods**

24 children with burned area more than 16%TBSA or deep dermal burned area greater than 10%TBSA, age ranging from 2 to 10 years old were included with randomly grouping according to single or double numbers. PICC catheter was implanted under non-anesthetic ultrasound guidance in the control group, while under ultrasound guidance with general anesthesia for control group. Success rate of the first implantation, implantation time and the incidence of subcutaneous hemorrhage were calculated.

**Results**

The success rate of the first implantation in the observation group was higher than the control group (x2 = 14.4, P = 0.00015 < 0.05); implantation time in the observation group was significantly shorter than the control group (t = 5.665, P < 0.05); the incidence of subcutaneous hemorrhage in the observation group was lower than the control group (x2 = 9.882, P = 0.00167 < 0.05).

**Conclusions**

PICC provides an ideal venous access for children with severe burns and solves difficulties on peripheral venous catheterization in children. However, the poor coordination in children reduces the success rate of catheter implantation, and increases the incidence of mechanical phlebitis and venous thrombosis after catheterization. The ultrasound-guided implantation of PICC is a safe and practical catheterized method, which ensures the treatment of children with a increasing success rate. The upper elbow catheter implantation can avoid the traction and friction of the catheter effectively when children moving, reduce the occurrence of complications after catheterization, and open up a new intravenous way for the treatment of children with severe burns.

### Abstract Topic: Nursing Care in Burn Centre

#### S36 The Effectiveness of Supervised Exercise Training for Venous Leg Ulcer: A Quasi-Experimental Study

##### Shu-Fen, Kao

###### Head Nurse, Burn Center, Department of Nursing, Taipei Veterans General Hospital ,Taiwan

**Background**

Venous leg ulcers were important medical problem. Standard evidence-based care includes exercise training, which have been shown to accelerate healing, improve quality of life, and likely reduce cost.

**Materials and Methods**

This study was quasi-experimental research design. The purpose of the study was to develop and evaluate the effect of a exercise training programs (aerobic exercise with resistance training) in venous leg ulcer patients. Then to compare the effectiveness about lower extremity muscle strength, wound healing、medical cost.

**Results**

After paired samples t test found that aerobic exercise and resistance combined aerobic exercise in the two groups after the implementation of four weeks before the implementation of significant differences (P <.05), four groups after the implementation of the four weeks before the implementation of significant Differences (P <.05).

**Conclusions**

Exercise training for lower extremity venous ulcer patients with lower limb muscle strength has significant progress in the effectiveness of exercise training on the peripheral circulation benefits, some studies have shown that after exercise therapy ankle - arm arterial blood pressure index was significantly improved.

### Abstract Topic: Nursing Care in Burn Centre

#### S37 Safety of placing peripherally inserted central catheter via femoral versus non-femoral vein in burn children: a retrospective analysis

##### Yanxu Lu^1^, Rongchan Wu^2^, Ying Wu^2^, Xiaoyuan Huang^2^, Li Xiao^2^, Li Xie^2^

###### ^1^Changsha Medical University, Changsha 410219, Hunan, China; ^2^Department of Burns and Plastic Surgery, Xiangya Hospital, Central South University, Changsha, Hunan, 410008, P. R. China.

####### **Correspondence:** Ying Wu

**Background**

Placing peripherally inserted central catheter (PICC) is currently popular among burn patients. However, most subjects of previous studies have been adults and insertion sites are predominantly non-femoral veins. Such an approach is quite limited for PICC studies of burn children.

**Materials and Methods**

From February 2014 to August 2018, retrospective analysis was performed for 76 burn children with implanted PICC at our department. The relevant data included general demographics, catheter characteristics and complications.

**Results**

The age range was 3 to 120 months. A total of 79 single-lumen 3/4F catheters were implanted. The insertion sites were non-femoral vein (25 catheters, n=22) and femoral vein (54 catheters, n=54). The mean indwelling duration of non-femoral and femoral veins was 27.28(9-47) and 22.33(2-130) days respectively. Inter-group statistical differences existed in insertion mode and insertion length (P<0.05). In non-femoral venous catheterization group, insertion was blind (n=14, 56.0%) and ultrasound-guided (n=11, 44.0%); in femoral venous catheterization group, placement was blind (n=54, 100%). There were statistically significant inter-group differences in puncturing mode and indwelling duration (P<0.05). The overall incidence of PICC complication was 17.7%. Neither group had any occurrence of catheter-related infections. Based upon generalized linear model, no inter-group statistical differences existed in adjusted age, burn area, hospitalization length, number of operation, puncturing mode or complication (P=0.498>0.05). All children were cured and discharged.

**Conclusions**

For burn children, placing PICC via femoral and non-femoral veins are both safe and effective. For those with difficult accessing at other sites, PICC via femoral vein is also a safe option.

### Abstract Topic: Nursing Care in Burn Centre

#### S38 Construction of Standardized Nursing System for Patients with Severe Burn Treated with Citrate as An in vitro Anticoagulant for Blood Purification Therapy

##### LiNing, ChenHualing

###### The First Affiliated Hospital of Army Medical University, Chongqing, China

####### **Correspondence:** LiNing

**Background**

The purpose of this study is to construct a scientific, systematic and applicable standardized nursing system for patients with severe burn treated with citrate as an in vitro anticoagulant for blood purification therapy, in order to provide the basis for standardized nursing for blood purification therapy with citrate as an in vitro anticoagulant.

**Materials and Methods**

A multidisciplinary team was built. Relevant domestic and foreign documents were referred to and 26 experts were consulted for two rounds by Delphi method and a preliminary standardized nursing system for patients with severe burn treated with citrate as an in vitro anticoagulant for blood purification therapy was constructed.

**Results**

The response rate of two rounds of expert consultation was 91.10% and 100.00%, respectively; the authority coefficient (Cr) was 0.943. The expert coordination coefficient was 0.501 and 0.713, respectively. Hence, the expert coordination was high (P < 0.05). Four first-grade indexes, including evaluation system, standard operation system, early warning and nursing system for complications and quality control system, and 19 second-grade indexes and 43 third-grade indexes were established.

**Conclusions**

The standardized nursing system for patients with severe burn treated with citrate as an in vitro anticoagulant for blood purification therapy can help improve continuity of care, reduce unplanned extubation rate and operation opportunity disruption rate, lower rate of complications and shorten average stay in ICU and cut expenses and also improve quality of care.

### Abstract Topic: Nursing Care in Burn Centre

#### S39 Application of standardized communication (SBAR) model handover sheet in transfer and handover of emergency patients with extensive burns

##### LiNing, LiuTingmin

###### The First Affiliated Hospital of Army Medical University, Chongqing, China

####### **Correspondence:** LiNing

**Background**

The purpose of this study is to discuss the effect of application of standardized communication (SBAR) model in the handover of emergency patients with extensive burns for the large amounts of information and specialized contents.

**Materials and Methods**

Prospective comparison study was conducted. From January to December 2017, 45 emergency patients with extensive burns in our hospital formed a control group and were transferred and handed over by conventional model. From January to December 2018, 54 emergency patients with extensive burns in our hospital formed an experimental group. On the basis of conventional handover model, the emergency patient handover sheet of the experimental group was prepared by following the SBAR standards, covering the patient name, diagnosis and other basic information; brief medical history, current drug and oxygen treatment, activity and skin; state of consciousness, vital signs and life-threatening factors; therapeutic measures adopted and cautions. A statistical analysis was conducted on the two groups’ observation indicators such as adverse event rate, handover duration and satisfaction of medical staff.

**Results**

The adverse event rate of the observation group was reduced by 8.1%, average handover duration was 13±4.5 minutes shorter, the doctors’ satisfaction with nurses’ handover was increased by 3.8% and the nurses’ satisfaction with handover process was increased by 5.6% (P<0.05).

**Conclusions**

The SBAR standardized communicate model is a safe, effective, evidence-based and result-oriented communication model, which can improve the critical thinking ability of nurses, improve satisfaction of medical staff and patients, reduce communicate-related adverse event rate and shorten the handover duration to better guarantee the handover safety of emergency burn patients.

### **Abstract Topic:** Nursing Care in Burn Centre

#### S40 Pathogen Characteristics of CVC-related Bloodstream Infection in Extensively Burned Patients and Analysis on Effects of Bundles of Care

##### LiNing, GongYali

###### The First Affiliated Hospital of Army Medical University, Chongqing, China

####### **Correspondence:** LiNing^1^

**Background**

The purpose of this study is to investigate the pathogen characteristics of CVC-related bloodstream infection in extensively burned patients, drug tolerance and effects of bundles of care.

**Materials and Methods**

Convenience sampling was used to select 224 cases of extensive burn occurred from January 2013 to December 2015 as the control group from the ICU of Department of Burns of a 3A hospital in Chongqing, and 234 cases of extensive burn occurred from January 2016 to December 2018 as the experiment group. And the pathogen characteristics and drug sensitivity of CVC-related bloodstream infection of the two groups were taken as observational indexes for statistical analysis.

**Results**

There was a significant difference between the control group and the experimental group in the detection of G+, G-and fungi. Acinetobacter baumannii, Pseudomonas aeruginosa G+ Staphylococcus aureus and fungi in the control group were significantly lower than those in the control group. The G-Klebsiella pneumoniae in the experimental group was significantly higher than that in the control group (P < 0.05). In the control group, the detection rate of multi-drug-resistant bacteria increased during 2013 to 2015 and then decreased year by year after reaching the peak in 2015 (P < 0.05).Most of the gram-negative bacteria were only susceptible to β-lactam,carbapenems and aminoglycosides with relatively low resistance rates, But the resistance rates showed an increasing trend (P < 0 05).Especially, the drug resistance of Klebsiella pneumoniae was serious (P < 0.05).

**Conclusions**

The detection rate of pathogen causing burn unit-acquired infection was high and most of the pathogens were highly drug-fast. The application of bundles of care to strengthen infection control management in hospital wards and reasonable use of antibacterial agents have good effects on reducing drug-fast bacteria, improving anti-infectious treatment and controlling nosocomial infection rate.

### Abstract Topic: Nursing Care in Burn Centre

#### S41 Airway management experience in batched burn patients combined with inhalation injury

##### Liping Zhang^1^, Huaqing Chen^1^, Haiping Hua^1^, Xingang Wang^1^, Hao Xu^1^, Jiyong Jing^1^, Junfeng Xu^1^, Xiong Zhao^2^, Xiaojie Yue^2^, Chunmao Han^1^

###### ^1^Department of burn, second Affiliated Hospital of Zhejiang University, College of Medicine, Hangzhou, China; ^2^Department of Burn and Plastic Surgery, Children's Hospital of Zhejiang University, College of Medicine, Hangzhou, China

####### **Correspondence:** Haiping Hua

**Background**

On July 5, 2014, a total of 32 passengers were hospitalized for burns in Hangzhou bus fires. 32 patients had different degrees of inhalation injury. The medical team involved in the treatment has taken the principle of “optimized treatment and refined care” and actively treated respiratory tract burns with multi-disciplinary technical advantages, and achieved good results.

**Materials and Methods**

A multidisciplinary and specialized airway management team was established to implement airway management strategies in a comprehensive, whole-process and refined manner. Team members including: respiratory therapist, nursing management specialist, responsible nurses, pediatrician, burn doctors, nutrition therapist, ENT doctors, ICU doctors, psychiatrist, hospital-acquired infection control department. This study with a focus on emergency respiratory assessment and treatment, airway management, anti-shock and drug used, psychological intervention and environment management, etc

**Results**

There were 32 burn patients with different degrees of inhalation injury in this bus fire accident. By taking active airway management measures, 7 of 10 ventilator-assisted patients were successfully weaned on day 7 after injury by taking effective airway management measures. 14 days after injury, except for one severely ill patient, all other patients spontaneously breathed without serious pulmonary infection. The fiber bronchoscope revealed that the airway mucosa healed significantly without complications such as airway stenosis. Compared with previous patients with burns and respiratory tract injuries, we found that managing airways through multidisciplinary treatment can shorten ventilator offline time, reduce the incidence of lung infection. However, the healing time of the respiratory tract and the narrowing of the airway cannot be compared.

**Conclusions**

The multidisciplinary joint strategy fully highlights the professionalism of the various disciplines involved in treatment and play an important role in improving the efficiency and refinement of airway management in batched burn patients. The relevant experience is worth promoting

### Abstract TopicL: Nursing Care in Burn Centre

#### S42 A Systematic Review: Topical Sucralfate for Burn Wound

##### Loelita M Lumintang, Made S Adnyana, Nyoman P Riasa

###### Division of Plastic Reconstructive and Aesthetic, Surgery Department, Faculty of Medicine Udayana University/Sanglah General Hospital, Denpasar, Bali, Indonesia

####### **Correspondence:** Loelita M Lumintang

**Background**

Topical Sucralfate has been used for burn and non-burn skin and mucosal lesion with remarkable results. The healing rate of mucosa is faster than skin lesion. This systematic review was conducted to determine the benefits of topical sucralfate for burn wound, to show on which degree of burn wound it works, how it works and the outcomes compared to SSD as a gold standard of burn wound therapy

**Materials and Methods**

Databases were searched for relevant studies: google scholar, PUBMED, and ProQuest. Data sources were searched using MeSH terms: ‘topical sucralfate’ and ‘burn wound’ for all publications up to December 2018. All English papers were included. Both in vivo and in vitro comparative studies, performed in humans or animals, were included. A total of 2437 publications were found, of which 4 studies met the inclusion criteria and were relevant to be used in this systematic review. The primary outcome was burn wound healing.

Data from retrieved studies were reviewed and tabulated according to year of publication, study design, human or animal studies, characteristics of the population, and outcomes.

**Results**

A total of 2 randomized controlled trials, 1 case control trial and 1 observational comparative study were found. All trials are on second degree burn wound patients. Jadad score was used to assess the methodological quality of the article. Three publications demonstrated favorable outcomes with the use of topical sucralfate and one publication with no significant differences.

**Conclusions**

This systematic review shows a noticeably beneficial effect of topical sucralfate for burn wound. It is better than SSD in wound healing rate, decreased infection rate and enhancement of epithelialization with no local or systemic adverse reactions. However, multicenter RCT with larger sample size are needed to make recommendation for burn wound treatment.

### Abstract Topic: Nursing Care in Burn Centre

#### S43 The effects of h***umidity*** combined with oxygen therapy on face with superficial partial-thickness burn

##### Weiwei Wu

###### The first hospital of Jilin university, Changchun, Jilin, China

**Background**

To access the healing time on face with superficial partial-thickness burn when treated humidity combined with oxygen therapy.

**Materials and Methods**

This clinical trial was conducted with 68 hospitalized patients with superficial partial-thickness burn on face from October 2016 to February 2018 .They were randomly divided into two groups. In the control group,35 patients were treated with recombinant human granulocyte macrophage stimulating factor gel 3 times once a day and treated with exposure therapy. In the intervention group (33 patients), in addition to the control group’s treatment, active humidification combined oxygen therapy device to humidify the wound, which was performed 3 times a day,40 min once time. The humidification temperature was 37.0°C, the humidification humidity was 60°C,and the *oxygen concentration* is 50%.Compared the average healing time and comfort score about the wound between groups.

**Results**

The average of the healing time was (9.3 ± 1.5) days in the intervition group compared with( 11.7 ± 1.2 )days in the control group .There was significant difference between the two groups ( P < 0.05 ) .What’s more,The comfort degree of the wound was (95±8.2) points in the intervention group and (88±9.7) points in the control group .A statistically significant reduction was reported for the intervention group compared with the control group(P<0.05).

**Conclusions**

Humidity combined with oxygen therapy not only shorten the wound healing time but also improve wound comfort.

### Abstract Topic: Others

#### S44 A systematic review and meta-analysis of enteral glutamine supplementation in critically burned patient

##### Guanghua Guo, Lian Zhang, Mingzhuo Liu, Xincheng Liao

###### Department of Burn, The First Affiliated Hospital of Nanchang University, Nanchang, Jiangxi, China

####### **Correspondence:** Guanghua Guo

**Background**

After severe burns, the body is continuously in a state of high metabolism and decomposition. Severe burns after many amino acids in a state of output, such as skeletal muscle and organ of glutamine, alanine, arginine, they transport it, at the same time provide energy for liver function, and the satisfaction of energy on the formation of a new skin plays a very important role, including glutamine also has to maintain the intestinal immune function. The purpose of this meta-analysis is to collect, summarize and analyze the existing clinical trial data to determine whether intestinal glutamine supplementation can positively affect cicadas in critically burned patients

**Materials and Methods**

The following databases were searched by computer: PubMed, baidu Scholar, Cochrane clinical trial.The retrieval time is from the database building to February 2019.A randomized controlled trial of enteral GLN supplementation in critically burned patients was included.The quality of the included literature was evaluated using the cochrane manual, the data were combined, and statistical analysis was performed using the RevMan5.3 software

**Results**

A total of 13 articles, a total of 490 cases (control group, 242 cases of vs group, 248 cases), the evaluation standards of hospitalization days (the control group, 210 cases of vs the experimental group, 219 cases), wound healing rate of 118 cases (control group vs the experimental group 122 cases), infection rate of 134 cases (control group vs the experimental group 133 cases), 11, 6, 7) research and analysis, respectively

**Conclusions**

Enteric glutamine supplementation can reduce the incidence of infection in burn patients, promote wound healing, and reduce the number of days in hospital. This is the analysis result of a small trial, and a large randomized double-blind controlled trial is still in progress

### Abstract Topic: Nutrition and Metabolism

#### S45 Propranolol use in adult burn patients and dosing practices at Singapore General Hospital Burns Centre: Implication for safety

##### Hong Ngee Chan^1^, Jasmine CL Ong^1^, Jie Lin Soong^1^, Shin Yi Ng^2^, Yee Siang Ong^3^

###### ^1^Department of Pharmacy, Singapore General Hospital, Singapore; ^2^Department of Surgical Intensive Care, Singapore General Hospital, Singapore; ^3^Department of Plastic, Reconstructive & Aesthetic Surgery, Singapore General Hospital, Singapore

####### **Correspondence:** Hong Ngee Chan

**Background**

Propranolol has been shown to be efficacious modulator of post burn cardiac response and increase muscle protein synthesis in severely burned children. However, safety, efficacy and optimum dose in adults are less well established. The primary objective of our study is to investigate the safety of propranolol in adults with ≥20% TBSA burns. The secondary objective is to determine the propranolol dose levels that are tolerated by our local population.

**Materials and Methods**

We retrospectively reviewed medical records of burn patients between 21 - 65 years old with ≥ 20% TBSA burns that were on propranolol from January 2010 to October 2017. Propranolol dose and patient clinical data were obtained from hospital electronic medical records. Hypotension is defined as SBP < 100 or MAP < 60 mm Hg, and bradycardia as HR < 60 bpm. Descriptive statistics were used to report results.

**Results**

Forty-one patients met the inclusion criteria with median age of 31 years, median burn size of 50% TBSA and 39% had inhalational injury. Mortality was 19.5%. Median time to propranolol initiation was 3 days from hospital admission, Median episode of dose held was 4 per treatment course. Low blood pressure accounted for 39.8% propranolol dose withheld. Less than 5% dose withheld was associated with bradycardia, 25.9% due to nil by mouth or in operation, 28.9% with no reason stated. Some 43.9% of patient required vasopressor at some point. The median dose administered during hospital stay was 0.3 mg/kg/day (IQR 0.2 to 0.5 mg/kg/day).

**Conclusions**

This study indicates that our patients did not tolerate similar propranolol dose reported in other adult studies. Vasopressor use suggests presence of shock which may explain lower propranolol doses used and lower threshold to withhold doses. Future prospective, dose finding studies are needed to determine the optimum dose of propranolol in adult burns patients.

### Abstract Topic: Paediatrics Wound Management

#### S46 Application of music video finger function rehabilitation exercise in continuous nursing care of burned children

##### Wu Weiwei, Cai Duo, Xu Xiaochuan, Cheng Dan, He Tingting, Zhou Yan

###### Department Of Burn Surgery, The First Hospital of Jilin University, Changchun city, Jilin, China

####### **Correspondence:** Wu Weiwei

**Background**

To observe the application effect of music video finger function rehabilitation exercise in continuous nursing care of burned children.

**Materials and Methods**

From January 2017 to January 2018, 69 children were divided into control group and experimental group according to random number table, 34 cases in control group and 35 cases in experimental group. After discharged from hospital, the experimental group adopted the music video finger function rehabilitation exercises in the guidance method, guiding the children to do finger function exercises for 40 minutes once a day ;the control group was according to the routine guidance method after discharged, it is by using the words and pictures to guide the children and their families to do hand function exercises, by observing the total activity range (TAM) of the two groups 3 months after discharge to assess the joint range of motion and compare the satisfaction scores of family members of the two groups to perform the T test and χ2 test of the data.

**Results**

The TAM assessed good ratio in the experimental group was 82.85% (29/35),and the control group was 58.82% (20/34),which has statistical significant of the difference between the two groups(χ2=4.839, P=0.038); the family satisfaction score of the experimental group was (9.5 + 2.8) and the other was (8.2 + 2.1), which has statistical significant of the difference between the two groups(t=18.105, P=0.000)

**Conclusions**

Music video finger function rehabilitation exercise is conducive to the recovery of hand function of burned children, and the improvement of the satisfaction of continuous nursing care.

### Abstract Topic: Others

#### S47 Sollerman Test: A simple, effective & reproducible test for functional evaluation in reconstructed post burn hand deformity

##### Seema Rekha Devi, Paresh Baruah,Vaibhav Bhisikar, Sonali Uchil, Biraj Saikia

###### Plastic surgery Department, Gauhati Medical College & Hospital, Guwahati, Assam, India

####### **Correspondence:** Seema Rekha Devi

**Background**

Post Burn hand deformity is a common occurrence despite increasing sophistication of management of acute thermal injuries. Quality of life of burn survivors depend upon the functionality of the hand.: Functional outcome of post burn hand deformity is still vague and controversial. Main principle in severe hand burn deformity is restoring function rather than individual movement of all joints.

**Materials and Methods**

This is a three year study carried out at the Dept. of Plastic Surgery at GMCH, in 65 patients in 75 hands with unilateral and bilateral involvement with varying severity from January 2015 to March 2018, which includes patients with post burn hand deformity. Age group varied from 2 years to 45 years, mean age is 18.5 years. ‘Sollerman Hand Function Test is used (pre and post-surgery) which is a standardized method based on eight most common hand grips and 20 subtests of activities of daily living. For Final evaluation of the validity and reliability of the tests, scoring as described has been used.

**Results**

The results in the studied population showed reliability of the pre-operative prognostic analysis with the subjective estimation of hand function which was assessed with post-operative scoring of hand function.

**Conclusions**

The test is simple and reliable & gives a true picture of Grip Function in activities of daily living besides giving a moral boost to the patient in continuing the physical therapy for continued improvement

### Abstract Topic: Others

#### S48 Post Burn Breast Reconstruction, a challenge in plastic surgery

##### Seema Rekha Devi, Jyotirmay Baishya

###### Plastic surgery Department, Gauhati Medical College & Hospital, Guwahati, Assam, India

####### **Correspondence:** Seema Rekha Devi

**Background**

Burn injury to the anterior wall of the female breast can result in various deformities due to extensive scar contractures. The deformities may vary according to the age, type and nature, the depth and extent of injury. The deformities in pre pubertal breast can result in hidden breast or under developed breast, and/or destruction of NAC, whereas in a post pubertal breast, it may result in distortion of contour, with displacement, difficulty in lactation (NAC disfigured or destroyed). There is usually absence of the infra mammary fold, inadequate breast proportion, under development of breast and alteration of the nipple areola complex position.

**Materials and Methods**

In post burn breast reconstruction both functional and cosmetic aspects are taken into account including breast feeding and nipple sensation. Although some nipple sensation recovers, breast feeding cannot be achieved always when there is destruction of nipple areolar complex .Total 50 numbers of cases of post burn breast reconstructions done in last 10 years will be presented. Cases were classified according to severity into Type 1, Type 2, Type 3. Out of 50 cases Type I are 12 Type II 30 & Type III 8 cases & 17 cases pre pubertal and 33 post pubertal. All cases were managed with surgery followed by post-operative care including pressure garments, splints, massage and exercise.

**Results**

The patients were assessed on the basis of patient satisfaction, size and shape of the axilla and contour of the breast and position of the NAC from defined landmarks & complications if any. It was found to be aesthetically fair in 25, good in 19, and excellent in 6 cases. Complications included small graft loss (6), scar hypertrophy 8, re-contracture 4.

**Conclusions**

Knowledge of anatomy & good pre-operative planning and post-operative care gives good long term results. He video on a post burn Breast reconstruction shows steps of breast reconstruction and post-operative result.

### Abstract Topic: Others

#### S49 Difference in TBSA Estimation Between Emergency Room and Burn Unit in Second Tier Hospital in Central Jakarta

##### Sanjaya F Tanjunga^1^, Aditya Wardhana^2^, Gammaditya A Winarno^1^, Amani S Augiani^1^, An’umillah A Zidna^1^

###### ^1^Internship General Practitioner, Jakarta Islamic Hospital Cempaka Putih, Jakarta, Indonesia; ^2^Plastic Surgeon, Jakarta Islamic Hospital Cempaka Putih, Jakarta, Indonesia

####### **Correspondence:** Sanjaya F Tanjunga

**Background**

Burn TBSA estimation is essential to administer fluid resuscitation. There are some well-known methods including Rule of 9 and Lund-Browder Chart. This study aims to identify the difference in TBSA estimation in Emergency Room & Burn Unit.

**Materials and Methods**

This is a retrospective cross-sectional study. Samples are patients admitted to Jakarta Islamic Hospital Cempaka Putih (JIHCP) Burn Unit between April 2015-September 2018. Inclusion criteria are patients admitted to JIHCP burn unit with complete TBSA estimation between the emergency room and burn unit. Exclusion criteria are missing variables in records. The estimation of TBSA in emergency room is done by General Practitioner, while in burn unit is done by Plastic Surgeon.

**Results**

Admission of patients reached 160, and 142 patients were eligible in the inclusion criteria, most of it were adult male with average of 28.3 years old, suffering a grade II burn injury, caused by scald. There is a higher mean of TBSA estimation in the Emergency room with 15.83 (SD 12.21) compared to Burn Unit with 12.92 (SD 12.00). The maximum TBSA overestimation in ER reached 24% TBSA compared to BU, while the minimum being 0.5%. Maximum TBSA underestimation reached 20%, and minimum underestimation was also 0.5%. In average, ER overestimated about 6.7% TBSA, and underestimated about 2.8% TBSA compared to BU.

**Conclusions**

The emergency Room has the tendency of overestimating the TBSA, with almost 3% difference in mean (p<0.05). Further investigation is needed to analyze which TBSA estimation method is used in each room.

### Abstract Topic: Others

#### S50 Nanotechnology for burns: Epicite ^hydro^

##### Joel Casas-Beltran, Pablo Rodriguez-Ferreyra

###### Burn Unit, Mexican Institute of Social Security, Hermosillo, Sonora, México

####### **Correspondence:** Joel Casas-Beltran

**Background**

Biotechnology provides access to modern new natural materials for medicine. Epicite ^hydro^ is an innovative material made of biotechnologically generated pure cellulose and water. Due to its highly structured three dimensional fiber network and water content of at least 95%, it provides skin with water and supports it in the process of wound regeneration.

Objectives: To demonstrate the safety and clinical outcomes of using epicite ^hydro^ in burns, as an alloplastic temporal skin substitute for the treatment of burns

**Materials and Methods**

Records of all patients attended for burns treated with epicite ^hydro^, at least 6 months prior to the present study, analyzing sex, cause, depth, and percentage affected, as well as pain scale, time of healing process and complications. Vancouver scar scale recording data at 6 months, after treatment

**Results**

From October 2017 to March 2018, 37 files of patients with burns treated with epicite ^hydro^ were collected. Patient demographics was 63% male (23), 37%,female (13), mean age 36 years (range 2 to 58 years), mean percentage 15% (range 1% to 25%), 73% 2nd degree burn (27 cases), 27% for 3rd degree burn (10 cases). Cause of burns 44% fire, 40% scald, 5% contact whit hot material and 1% chemical. The mean pain scale 2 (range 1-4), mean epithelization 7 days (range 5-14 days), 3rd degree burns generated granulation tissue (mean day 17). No complications, infections or allergic reactions. Vancouver scale at 6 months averaging 2.9 (2nd degree)

**Conclusions**

Epicite ^hydro^ can be safety used on burned skin surfaces where the epithelium is missing. The structured fiber network as a cell free, alloplastic temporal skin substitute reduce pain and prevent infections. The biomaterial allows an optimized fluid homeostasis, creating an environment that stimulates the wound healing process, helps reducing time of reepithelization and with low scaring, making a promising novel treatment for burns

### Abstract Topic: Others

#### S51 Epidemiology of burns patients presenting to emergency department in a secondary care hospital in East Nusa Tenggara, Indonesia from 2016 to 2018

##### Agustini Song

###### Emergency Department, S.K. Lerik Regional Public Hospital, Kupang City, East Nusa Tenggara, Indonesia

**Background**

Although burn remains as a significant, preventable cause of morbidity and mortality in Indonesia, there is still no national guideline of burn in this country. Despite some extensive studies about the epidemiology of admitted burn patients in some burn centers in Indonesia, the study involving the whole population, including the minor burn outpatient admitted in emergency department (ED), is still scant. The objective of this study is to provide the characteristic of all burn patient as a guideline for burn prevention planning program in Indonesia.

**Materials and Methods**

The medical records of patients with burns admitted to the ED of S. K. Lerik Regional Public Hospital between January 2016 and December 2018 were evaluated retrospectively and analyzed.

**Results**

From 2016 to 2018, the average number of admission was 4.3 ± 2.26 patients per month and 52 patients per year. Of the 155 included burn patients, 60.7% were male and 56.8% of all patients were in the working age group (15-64 years old). The majority of patient (31.6%) suffered from second degree (superficial partial-thickness) burn. There were 45.2% patients with no health insurance, accounted for the majority of the subjects. A total of 102 patient (65.8%) were treated as outpatient, with 12 patients (7.7%) discharged against medical advice due to cost issue.

**Conclusions**

Working age group, which is 15-64 years old, with male predominance is at the highest risk of suffering burn injury. Most burn patients in this study still had no health insurance, therefore contributed to the high number of discharge against medical advice which hindered them from getting the best possible treatment, hence the increasing morbidity and mortality. Occupational burn prevention and the socialization of public health insurance should be the priorities in planning the program for burn prevention in this country.

### Abstract Topic: Others

#### S52 The importance of absolute rest in the management of lower leg burns

##### Yosuke Ojima, Munenori Sato, Hajime Matsumura

###### Department of Plastic and Reconstructive Surgery, Tokyo Medical University, Shinjuku, Tokyo, Japan

####### **Correspondence:** Yosuke Ojima

**Background**

In the management of lower leg burns, absolute rest of affected limbs is quite important for the success of treatment. Sometimes, we experience the cases that the patients get better soon after they admitted to the hospital and just rested their affected limbs on the bed, while they have had refractory wounds in the outpatient clinic. The purpose of this study was to evaluate the importance of absolute rest in the lower leg burns.

**Materials and Methods**

We retrospectively studied 125 lower legs of 108 patients who came to our hospital with Second-degree or Third‐degree lower leg burns between October 2014 and April 2018. Age, sex, mechanism of injury, percentage of the total body surface area (%TBSA), the treatment situation (admission or outpatient clinic treatment), the method of treatment (conservative or surgical treatment), wound healing period and complications were investigated.

**Results**

We included 57 male lower extremities and 68 female lower extremities. Mean age was 41 years old, and the most frequent mechanism of injury was scald burn. 90 lower extremities were treated in the outpatient clinic, and 35 lower extremities were treated in hospital. Mean %TBSA were 0.85% and 1.51%, respectively. 13 cases were converted from outpatient clinic to admission treatment because of refractory burn wounds. Mean %TBSA of them was larger than that of the cases treated in the outpatient clinic to the end (1.45% vs 0.75%; p<0.05).

**Conclusions**

Under the outpatient treatment, the patients cannot have absolute rest because of their daily lives, and sometimes it lead to a worsening of their burn wounds. Considering from our study, the lower legs burns more than 1.5% TBSA should be hospitalized unless keeping bed rest or leg elevation.

### Abstract Topic: Others

#### S53 An Retrospective analysis on Marjolijn’s Ulcer

##### Huibin Li^1^, Xing Yang^1,2^, Chenghao Hu^1,2^, Dongyu Li^3^, Feng Zhao^1^, Yuhui Dongye^1,2^

###### ^1^Department of Burns and Plastic Surgery, People's Hospital of Linyi, Linyi, Shandong 276000, China; ^2^School of Clinical Medicine, Weifang Medical University, Weifang, Shandong 261000, China; ^3^College of Agricultural and Life Science, University of Wisconsin-Madison, Madison, WI, 53706, USA

####### **Correspondence:** Huibin Li

**Background**

To investigate the clinical feature, diagnosis, and treatment for Marjolijn’s Ulcer.

**Materials and Methods**

Analyze the Clinical resource of 32 cases of patients who was diagnosed with Marjolijn’s ulcer pathologically since 2005-2018 in Linyi People’s Hospital.

**Results**

All 32 cases are chronic. 29 cases (90.62%) are ulcerous and 3 cases (9.37%) are hyperplastic. In addition, 22 patients (68.75%) in this study are above 50’s. The primary injury of all cases is due to flame or thermal liquid. Sorted by anatomical locations, 23 cases (71.87%) originated in joint, followed by 6 cases (18.57) in torso. Only 3 cases of Marjolijn’s ulcer stems from head, accounting for 9.37%. The latent period ranges from 2-25 years, 8.43 in average. Further analysis proved no relationship between the age and latent period. According to Immunohistochemistry(IHC) staining, 27 cases (84.37%) are squamous carcinoma. Among these cases, 26 cases are well-differentiated whereas only one case is moderate-differentiated. After surgical excision, reoccurrence is observed only in moderate-differentiated case.

**Conclusions**

Marjolijn’s ulcer is more frequently observed among patients above 50s. IHC is the golden rules for diagnosis. Early intervention such as surgery will yield good prognosis for well-differentiated squamous cancer (SC) whereas prognosis for moderate SC is not optimal. Therefore, early diagnosis, intervention, and management remains an important factor for preventing or treating Marjolijn’s ulcer.

### Abstract Topic: Others

#### S54 Sera from Diabetic ulcer patients induced inflammatory and senescence of vascular endothelial via activating JMJD3

##### Lian Wang^1,2,^ Juanjuan Xing^1^, Jinhua Luo^1^, Xincheng Liao^1^, Youlai Zhang^1^, Tianning Wu^3^, Fei Guo^1^, Guanghua Guo^1^

###### ^1^Burn center of the First Affiliated Hospital of Nanchang University, Nanchang, Jiangxi, China; ^2^Graduate school of Nanchang University, Nanchang, Jiangxi, China; ^3^Affiliated High School of Nanchang University, Nanchang, Jiangxi, China

####### **Correspondence:** Fei Guo

**Background**

To analyze the effect of sera from diabetic ulcer patients on the proliferation, migration, senescence, activation, and injury of vascular endothelial cells, and the underlying epigenetic mechanisms.

**Materials and Methods**

The serum pools of diabetic ulcer group and healthy control group was established accordingly. Human umbilical vein endothelial cell (HUVECs) were cultured with the conditioned medium containing 5% human serum, with or without the histone H3K27me3/2 demethylase specific inhibitor, GSK-J4. Cell viability detected by CCK-8; Cell senescence measured by β-galactosidase in situ staining assay; Cell cycle analysis with flow cytometry; Cell migration and repairing ability scratch test; The expression of JMJD3 and methylation status of histone H3K27 in HUVECs monitored by laser scanning confocal microscopy; Gene expression of related factors determined by RT-PCR.

**Results**

The human diabetic ulcer sera promote HUVECs cell senescence and induce the G2-M phase arrest within 48h-incubation. We found that within the scratch repairing area, the cell density of the diabetic ulcer group decreased significantly comparing with control group. Meanwhile, The human diabetic ulcer sera induced up-regulation of JMJD3, a histone demethylase, which down-regulated H3K27me3/2 levels in the nuclei of HUVECs. Importantly, treatment with GSK-J4, can reverse the diabetic ulcer sera- induced senescence and G2-M phase arrest via restoring the H3K27me2/3 levels. We measured the expression of factors related to senescence, cell cycle arrest and cell migration. We found that eNOS, RAGE122, IL-1β, IL-6, IL-12 and TNF-α gene expressions were significantly up-regulated by diabetic sera and GSK-J4 treatment reversed effects. Interestingly, while the diabetic sera treatment down-regulated the expressions of ICAM-1, VEGF192 and TGF-β, GSK-J4 treatment rescued the aforementioned genes expression.

**Conclusions**

Our findings indicate that the human diabetic ulcer sera induce human endothelium senescence, cell cycle and functional disorder by activating JMJD3 signaling which down-regulating the global methylation level of H3K27 and promoting the expressions of pro-inflammatory and RAGE genes.

### Abstract Topic: Infection Control and Diseases

#### S55 In-vitro Influence of IL-10 modified hAMSCs on fibroblast and macrophage phenotypic transformation

##### Guangtao Huang, Zairong Wei, Dali Wang

###### Department of Burn and Plastic, Zunyi Medical University, Zunyi,Guizhou, China

####### **Correspondence:** Guangtao Huang

**Background**

In chronic wounds, the wound remains in the inflammatory phase, the proliferative and remodeling stages do not readily occur. There is a need to find better strategies to promote chronic wound healing, especially of more effective regulation inflammation and promote cell proliferation in chronic wounds remain a challenge. Both macrophages and fibroblasts play key roles in inflammation regulation and tissue repair of wound healing. Mesenchymal stem cells (MSCs) and IL-10 have been shown to accelerate open wound healing by regulating the inflammatory response and promoting fibroblasts proliferation.

**Materials and Methods**

The study explore the influence of IL-10 modified hAMSCs on macrophage phenotypic transformation and fibroblast in vitro, in order to improve MSCs biological effects of promote chronic wounds healing

**Results**

Our results showed IL-10 modified hAMSCs promoted the transformation of inflammatory M1 macrophages to tissue-repaired M2 macrophages,and showed the better capability of promoting fibroblast proliferation and repair gene expression than hAMSCs alone.

**Conclusions**

It may have great potential in promoting the chronic wounds healing.

### Abstract Topic: Others

#### S56 The epidemiology and clinical characteristics of epilepsy burn patients in the southwest of China

##### Song Wang, Xiaorong Zhang, Gaoxing Luo

###### Institute of Burn Research, Southwest Hospital, Third Military(Army)Medical University, Chongqing, China

####### **Correspondence:** Gaoxing Luo

**Background**

The epidemiology of epilepsy burn patients has been reported in several countries and areas. However, no such study has been conducted in China. The aim of the study is to investigate the epidemiology of the epilepsy burn patients and the effect of an epileptic seizure on the clinical characteristics of burn patients.

**Materials and Methods**

The collected burn registry data from patients admitted to the Southwest Hospital burn centre from January 2011 to December 2017 was used to do a retrospective cohort study. A propensity score method was applied to reconstruct a 1:1 matched-pair cohort between burn patients secondary to an epileptic seizure to those not.

**Results**

Of the 7,683 patients, 66 (0.86%) burn patients were secondary to an epileptic seizure. Among 66 epilepsy burn patients, 48.3% were female, the median age was 28.5 years(IQR:20.8-45.2), a flame injury was the most common etiology, the incidence of burns peaked on January, and the incidence was highest in upper limbs and lowest in the perineum. Compared to the non-epilepsy group patients, the epileptic seizure resulted in the 3rd-degreee TBSA, cost, LOS, cost/TBSA and LOS/TBSA significantly increased. However, a significant decrease was found in the superficial 2nd-degree TBSA and no difference was shown in deep 2nd-degreee TBSA and TBSA. The epileptic seizure showed a strong association with full-thickness burns (OR 8.52, 95% CI 4.21–17.23).

**Conclusions**

Our results suggested that an epileptic seizure may have a significant effect on the epidemiology and clinical characteristics of burn patients.

### Abstract Topic: Others

#### S57 A Prospective Comparative Study on the Effectiveness of Two Different Non-Adherent Polyurethane Dressings on Split-Thickness Skin Graft Donor Sites

##### Darshini Devi Rajasegeran^1^, Fazila Binte Abu Bakar Aloweni^1^, Chong Si Jack^2^, Lim Xinyi^1^, Saranya Chandra Sekaran^1^, Zhang Lei^1^, Lim Li Pin Brenda^1^, Kok Yee Onn^1^

###### ^1^Singapore General Hospital, Singapore; ^2^Association of Burn Injuries, Singapore

####### **Correspondence:** Darshini Devi Rajasegeran

**Background**

The current wound management SSG donor site in SGH Burns Unit is to apply ALLEVYN Non Adhesive. Numerous new polyurethane dressings with enhanced properties, such as BETAplast™N, are now available. However, there is limited research regarding their efficacy.

This is a prospective comparative clinical study analyzing differences in exudate absorbency, ease and pain on dressing removal and epithelization between Allevyn and Betaplast.

**Materials and Methods**

Patients in the general ward undergoing SSG were recruited. Allevyn and Betaplast were applied on the same donor site after SSG harvesting. Dressings were secured using OPSITE film and bandage. Absorptive capacity was assessed daily using an absorbency grading chart. Dressing change was done on POD 5. Ease of dressing removal was assessed with a visual chart, and pain score using the Wong-Baker Pain Scale. The percentage of full re-epithelization in terms of surface area for each dressing was assessed.

**Results**

The exudate absorption of Allevyn and Betaplast comparing between POD 1 and POD 5 was significant (P<0.05), indicating that both dressings continued to absorb exudate from POD 1 to POD 5. When comparing absorption from one POD to the subsequent POD, absorption for Allevyn was significant (P<0.05) only from POD 1 till POD 4, while absorption for Betaplast remained significant (P<0.05) from POD 1 until POD 5. There was no significant difference (p=0.05) between Betaplast and Allevyn for the percentage of re-epithelization. Descriptive statistics indicated higher mean percentage for Betaplast (mean= 62.73%) compared to Allevyn (mean= 49.09%), with 7 out of the 11 patients having better re-epithelization with Betaplast. No differences in ease of removal and pain score were found.

**Conclusions**

Compared to Allevyn, Betaplast has better capacity and is more effective in exudate absorption. Betaplast may have a faster rate of re-epithelisation compared to Allevyn, allowing faster donor wound healing and improving overall patient recovery.

### Abstract Topic: Others

#### S58 A Clinical Study on Keloid Formation After Hypertrophic Syndactyly Release

##### Zhu Wei^1^, Tian Xiaofei^2^

###### ^1^Institute of Burn Research, Southwest Hospital, State Key Laboratory of Trauma, Burns and Combined Injury, the Army Medical University, Chongqing 400038,China; ^2^Department of Burns and Plastic Surgery, Children’s Hospital of Chongqing Medical University, Ministry of Education Key Laboratory of Child Development and Disorders, China International Science and Technology Cooperation base Child development and Critical Disorders, Chongqing Key Laboratory of Pediatrics, Chongqing 400014,China

####### **Correspondence:** Tian Xiaofei

**Background**

We have shown that keloid formation after syndactyly release is associated with pachydactyly and skin-grafting in the previous retrospective study, To further explore the clinic cause of keloid formation after syndactyly release

**Materials and Methods**

The syndactyly appearance was preoperative evaluated for all patients from January 2015 to January 2017. Child with pachydactyly, carried out needle-scar response experiment firstly, according to the preliminary experimental results and the patients’ opinions decide whether perform syndactyly release furtherly. Postoperative skin grafting and scar hyperplasia were followed up

**Results**

There are 10 cases with the preoperative pachydactyly in two years. Among them, needle-scar response prediction experiments were carried in 5 cases,9 toes with pachydactyly occurred keloid, the other normal toe did not develop keloids after surgery; There were 6 cases performed syndactyly reconstruction, 9 toes with pachydactyly occurred keloid, 4 normal toes did not. 6 toes skin- grafting and 7 toes no

**Conclusions**

Keloid formation after syndactyly release is related to the local damage of the toe with pachydactyly, but not significantly correlated with the skin-grafting

### Abstract Topic: Others

#### S59 Selective mitophagy protects against early burn-wound progression via eliminating effects of damaged mitochondria

##### Songxue Guo^1^, Chunmao Han^2^, Quan Fang^1^, Liping Zhang^2^

###### ^1^Department of Plastic Surgery, the Second Affiliated Hospital Zhejiang University School of Medicine, Hangzhou, China; ^2^Department of Burns, the Second Affiliated Hospital Zhejiang University School of Medicine, Hangzhou, China

####### **Correspondence:** Songxue Guo

**Background**

Deep burn-wound undergoes a dynamic progression in the initial or peri-burn area after insults. The stasis zone has a higher risk of deterioration and is considered as a salvageable target for burn-wound progression. Although multiple efforts have been made to understand mechanism and treatment, few work explores the role of mitochondrial damage in this process and potential self-protection within human body.

**Materials and Methods**

A classic“comb”burn rat model was established in this study for further assessments. Hemodynamic and pathological evaluations were performed with helps of Laser speckle imaging and Hematoxylin and eosin staining. The changes of oxidative stress and apoptosis in the zone of stasis were detected by commercial kits. Mitochondrial damage and mitophagy were studied based on the results of transmission electron microscope and western blots. Fluorescence-labelled adenovirus for HIF-1α silence was applied to clarify the role of HIF-1α on regulating mitophagy in the zone of stasis.

**Results**

We found burn insults bring about typical ischemia and histological deterioration in the zone of stasis, in parallel with increases of oxidative stress and apoptosis. It was also found some clues about the involvement of mitochondrial damage in aforementioned changes. Furthermore, we detected typical mitophagy in burn-wound in the early stage after injury, presenting an opposite fluctuation to the conversion of burn-wound. Finally, we suggested HIF-1α is a key regulator on mitophagy in the zone of stasis with downstream involvement of BNIP3 and PARKIN.

**Conclusions**

Hence, we demonstrate burn-induced mitochondrial impairment contributes to the mobilization of injurious mechanism in the zone of stasis and mitophagy provides a way to protect against burn-wound progression via eliminating damaged mitochondria. Furthermore, our findings offer insights in mitochondrial quality control in burn-wound progression and suggest a novel concept that HIF-1α could be a potential therapeutic target in view of its regulation on BNIP3 or PARKIN-mediated mitophagy upstream.

### Abstract Topic: Others

#### S60 Severe burn injury alters intestinal microbiota composition and impairs intestinal barrier in mice

##### Yanhai Feng^1^, Yalan Huang^2^, Yu Wang^3^, Pei Wang^1^, Fengjun Wang^1^

###### ^1^State Key Laboratory of Trauma, Burns, and Combined Injury, Institute of Burn Research, Southwest Hospital, Third Military Medical University (Army Medical University) , Chongqing, China; ^2^Department of Military Nursing, School of Nursing, Third Military Medical University (Army Medical University), Chongqing, China; ^3^Department of Gastroenterology, Southwest Hospital, Third Military Medical University (Army Medical University), Chongqing, China

####### **Correspondence:** Yanhai Feng

**Background**

This study was aimed to investigate the changes of intestinal microbiota and barrier function in burned mice to further comprehend the mechanisms of burn-induced intestinal barrier dysfunction.

**Materials and Methods**

Samples were from mice inflicted with 30% TBSA full-thickness burns. The intestinal permeability, tight junction proteins’ expressions, ZO-1 localization, inflammatory cytokines’ expressions, and short-chain fatty acids (SCFAs)’ contents were determined. The microbial community was assessed via 16S rDNA Illumina sequencing.

**Results**

The intestinal permeability was increased after severe burn injury, peaking at 6 hour post-burn, with approximately 20 folds of the control (*p*<0.001). The expression of tight junction proteins (ZO-1, occludin, claudin-1 and claudin-2) was significantly altered (*p*<0.05). The ZO-1 morphology was dramatically changed following burn injury. The fecal SCFAs’ (acetate, propionate, butyrate, isobutyrate and isovalerate) contents were noticeably declined after burn injury (*p*<0.05). The expressions of pro-inflammatory cytokines (IL-1β and IL-6) in ileal mucosa were increased, whereas the expressions of anti-inflammatory cytokines (IL-4 and IL-13) were decreased following burn injury (*p*<0.05). In addition, burned mice showed an alteration of intestinal microbial community, such as decreased diversity, reduced *Bacteroidetes* abundance and increased *Firmicutes* abundance.

**Conclusions**

The severe burn-induced intestinal barrier dysfunction is along with the alterations of microbial community. Certain bacteria such as *Ruminococcaceae* may contribute to maintain intestinal homeostasis after severe burn injury.

### Abstract Topic: Others

#### S61 Burn Injuries Management in a Recently Established Burn Unit: 1 Year Experience and Outcome. Is conventional method still acceptable?

##### Chairani Fitri Saphira

###### Department of Neurosurgery, Plastic Surgery, Cardiothoracic Surgery and Urology, Dr. Mohamad Soewandhie General Hospital, Surabaya, Indonesia

**Background**

While the overall number of hospital visits due to burn injuries is noteworthy in Indonesia, resources for managing these patients are still relatively limited. This article will describe our experience and outcome in managing patients with burn injuries in a recently established burn unit of a primary referral hospital in a developing country. It will emphasize on the optimization of the limited available resources in the management process.

**Materials and Methods**

During 2018, all new of patients with burn injuries in Dr. Mohamad Soewandhie were recorded. The patients were categorized and analyzed with consideration of their management and outcome. All acute burns patients were resuscitated using modified Parkland formula as indicated. Burns wounds were debrided and dressed using paraffin gauze and silver sulfadiazine cream. No early excision performed. Patients with wounds that failed to heal secondarily in the expected period underwent definitive surgical wound coverage of skin grafts or flaps.

**Results**

A total of 104 new burn patients were recorded, with 39 of them with major burn injuries. Of the patients TBSA of burn, 67 had <10%, 20 had 10-20%, and 17 had >20%. Rate of surgery among patients who underwent surgery was 1.6 per patients. One patient with inhalational trauma and 1 pediatric patient died during hospitalization. Burn scar was the most reported complication during the follow up period.

**Conclusions**

This manuscript proves that burns management using conventional method and dressing is still applicable where resources are limited. The mortality rate is comparable to those of other developing countries and developed countries. But in terms of length of hospitalization and number of surgeries underwent, conventional method is inferior compared to current recommendation of practice guidelines for burn care. Conventional method should be slowly replaced with a more modern and up to date method along with the development of accessible resources.

### Abstract Topic: Others

#### S62 Investigation and analysis of burn pain of inpatients in southwest China

##### Huang Min, Yan Hong

###### Department of Plastic and Burn Surgery, the Affiliated Hospital of Southwest Medical University, Luzhou, Sichuan, 646000, P.R.China

####### **Correspondence:** Huang Min

**Background**

To understand the status of burn patients' perception and attitude towards burn pain in southwestern China, and to provide reference for improving the pain management level of medical staff in burn department

**Materials and Methods**

Questionnaire survey of 219 burned hospitalized adult patients in 8 tertiary hospitals with independent burn specialists in Southwest China

**Results**

95.83% of the patients thought that the pain response after burn was normal and should be tolerated as much as possible; 66.67% of patients were worried about side effects of painkillers; 35.33% patients did not receive any analgesia during hospitalization; Preoperative analgesics were used in 33.33% of patients before dressing change; When the patients reported to the medical staff that the pain was unbearable, the rate of receiving analgesic treatment within 10min was 73.52%; 46.58% of patients think that medical staff are concerned about their pain

**Conclusions**

Inpatients with burns in southwest China are lack of knowledge of pain, so pain knowledge education should be strengthened.At the same time, the pain management during hospitalization is not standardized, and individual differences are large. Therefore, the pain management of burn patients should be strengthened, so as to improve the quality of pain management of burn patients and alleviate their pain

### Abstract Topic: Others

#### S63 Characteristic of blast injury patients from Idi Rayeuk illegal oil mining explosion in East Aceh, Indonesia

##### Syamsul Rizal^1^, Muhammad Jailani^1^, Mirnasari Amirsyah^1^, Anastasia Dessy Harsono^2^, Meilya Silvalila^3^, Emil Muzammil^4^

###### ^1^Plastic, Reconstruction, and Aesthetic Surgery Department, Medical Faculty Syiah Kuala University/ Zainoel Abidin General Hospital, Aceh, Indonesia; ^2^Plastic, Reconstruction, and Aesthetic Surgery Department, Medical Faculty Syiah Kuala University/Gatot Soebroto Army Hospital, Jakarta, Indonesia; ^3^Emergency Medicine Department, Medical Faculty Syiah Kuala University/ Zainoel Abidin General Hospital, Aceh, Indonesia; ^4^Medical Research Unit, Medical Faculty Syiah Kuala University, Aceh, Indonesia

####### **Correspondence:** Emil Muzammil

**Background**

Blast injury is a complex and challenging burn case because could affect many person in one incident. The aim of this study was to analyze the characteristic of all hospitalized patients from a gas explosion incident occurred in April 25th 2018.

**Materials and Methods**

This is a retrospective descriptive study. All data was collected from medical record in Zainoel Abidin General Hospital, data that was taken including gender, total body surface area (TBSA), severity of burn, laboratory examination, and cause of death.

**Results**

From total 39 burn injury patients from the incident, 9 patients were referred to Zainoel Abidin General Hospital, but one patient died in transfer process. There were 7 male patients and 2 female patients. From all patients, 7 patients have TBSA >50%. All patients were afflicted with second degree burn injury. Mortality rate was 77.77%, death mostly caused by severe infection.

**Conclusions**

Good triage, early adequate resuscitation, and primary emergency care before patient transfer have important role to achieve optimal of therapy for burn injury cases from explosion incident that affect many person at once.

### Abstract Topic: Others

#### S64 Age and gender difference on the epidemiology and outcomes of burn patients in southwest of China: a 15-year retrospective study

##### LiNing^1^, WangZhonghua^2^

###### ^1^The First Affiliated Hospital of Army Medical University, Chongqing, China; ^2^School of Nursing, Army Medical University, Chongqing, China

####### **Correspondence:** LiNing

**Background**

The purpose of this study was to investigate the epidemiology, outcome, cost and risk factors among burn patients in one of the Chinese earliest specialized burn centers in the southwest China.

**Materials and Methods**

This 15-year retrospective study was performed at the Institute of Burn Research of the. Information regarding demographic, burn characteristics, the burn severity of ABSI and were collected, calculated and compared. Descriptive statistics, t-test,and x2 test were conducted according to the data types.

**Results**

A total of 16,614 burn patients were included. The mean age was 26.18±21.74 years old, ranging from 257 days to 95 years old. The pediatric patients under the age of 18 years old accounted for 40.3%, while the adult patients under the age of 60 years old accounted for 53.9%. Scalding and flame were the two most common cause to burn injuries, comprising of 90.96% in total. head/face/neck, limbs and trunk were the most frequently occurred burn sites, with the number and the percent of 6972 (41.96%), 6105 (36.75%), and 6031 (36.30%), respectively. The average total body surface area (TBSA) was 13.58±16.81% (median 8%) with a range of 0.1~100%. A total of 802 (4.8%) patients had TBSA >50%. The presence of a burn with an inhalation injury was confirmed in 502 patients (3.02%). The average LOS was 32.53±68.11 days (median: 17days).

**Conclusions**

This 15-year retrospective study examined the epidemiology and outcomes of burn patients in southwest of China. The annual number of burn injuries has kept decreasing, which was partially attributed to the increased awareness and education of burn prevention and the improved burn-preventative circumstances. However, the burn severity and the economic burden were still in a high level. And the gender difference and age difference should be considered when making individualized interventions and rehabilitative treatments.

### Abstract Topic: Others

#### S65 The epidemiology of burns in Japan: A national burn registry-based study

##### Junichi Sasaki^1,2^, Hiroto Ikeda^1,3^, Yoshiaki Inoue^1,4^, Jiro Katahira^1,5^, Miyuki Kishibe^1,6^, Chu Kimura^1,7^, Yukio Sato^1,2^, Kiyotsugu Takuma^1,8^, Katsumi Tanaka^1,9^, Minoru Hayashi^1,10^, Asako Matsushima^1,11^, Hajime Matsumura^1,12^, Hiroshi Yasuda^1,13^, Yuya Yoshimura^1,14^, Daizoh Saitoh^1,14^

###### ^1^Academic Committee of the Japanese Society for Burn Injuries, Tokyo, Japan; ^2^Keio University Hospital, Tokyo, Japan; ^3^Teikyo University Hospital, Tokyo, Japan; ^4^University of Tsukuba Hospital, Ibaraki, Japan; ^5^Tokyo Women's Medical University Hospital, Tokyo, Japan; ^6^Kanazawa Medical University Hospital, Kanazawa, Japan; ^7^Hakodate Central General Hospital, Hokkaido, Japan; ^8^Kawasaki Municipal Hospital, Kanagawa, Japan; ^9^Nagasaki University Hospital, Nagasaki, Japan; ^10^Maebashi Red Cross Hospital, Gunma, Japan; ^11^Nagoya City University Hospital, Aichi, Japan; ^12^Tokyo Medical University Hospital, Tokyo, Japan; ^13^Hospital of the University of Occupational and Environmental Health, Fukuoka, Japan; ^14^National Defense Medical College Hospital, Saitama, Japan

####### **Correspondence:** Junichi Sasaki

**Background**

In Japan, national burn registry (NBR) has been operated by the Academic Committee of the Japanese Society for Burn Injuries (JSBI) since 2010. JSBI NBR is designed to improve the quality, outcomes, patient care, effectiveness by collecting and exchanging information.

**Materials and Methods**

Outline of JSBI NBR: Collect data of patients hospitalized with burns from the 111 burn centers certified by JSBI, 12542 cases between April 2010 and March 2018 (8 years).

**Results**

Acute phase admission occupies approximately 90.6% (n=11359). Etiology of injuries: fire/flame (38.3%), scald (36.5%), contact (8.7%), explosion (4.8%), smoke/inhalation (4.1%), chemical (3.1%), electric (1.6%) others/unknown (2.8%). Inhalation injury is complicated with 24.0% of all cases. The death including CPA is 1071 cases (9.4%). Etiology of death: shock/ organ failure (46.1%), infection (28.4%), CO poisoning (7.1%). Shock/organ failure is a cause of the top within one week. It is revealed that a cause of the top changes in infection afterwards. The mortality among cases with inhalation injuries was approximately 4 times of the non- complicated case.

**Conclusions**

The current state of Japan burn treatment was clarified by JSBI NBR. We will consider collaboration with international databases, e.g. WHO Global Burn Registry Form at designing the database.

Limitations: Our data set is simple and include only basic items. In particular, the outcome of JSBI NBR is shown in survival, cause of death and length of stay.

### Abstract Topic: Others

#### S66 B-Flow Combined color Doppler ultrasound technical indwelling in extensive burn patients with has application effect of invasive arterial pressure

##### Wu Weiwei, Cai Duo, Zhang dandan, Cheng Dan, Zhang wuming , Ma yan

###### Department of Burn Surgery, The First Hospital of Jilin University, Changchun city, Jilin , China

####### **Correspondence:** Wu Weiwei

**Background**

To investigate the application effect of two-dimensional gray-scale flow imaging (B-Flow) in detaining invasive arterial pressure (IAP) in patients with extensive burns

**Materials and Methods**

Randomly divided the 67 patients with invasive arterial pressure from January 2017 to June 2018 into experimental group and control group .There were 32 cases in the experimental group and 35 cases in the control group, the control group used the color Doppler ultrasound technical and the experimental group used B-Flow combined with color Doppler ultrasound technology. To select the standard artery blood detaining invasive arterial pressure, to compare the success rate of catheterization, catheter time and incidence of arterial thrombosis after intubation between the 2 groups;

**Results**

there were 30 cases of successful catheterization in the experimental group and 27 cases in the control group, which had statistically significant (P<0.05)of the success rate of catheterization between the two groups;The operation time of the experimental group was (18.23 + 1.08) min and the other was (11.45 + 1.13) min,which had a statistically significant(P<0.05) of the difference between the two groups (P<0.05);There were 0 cases of thrombosis in the experimental group and 6 cases in the control group,the incidence of thrombosis after catheterization in the two groups was(χ^2^=6.025, P<0.05),which the difference had statistically significant

**Conclusions**

B-Flow combined with color Doppler ultrasound technique can improve the success rate of invasive arterial catheterization in patients with extensive burn, shorten the catheterization time, and effectively detect the occurrence of arterial thrombosis.

### Abstract Topic: Others

#### S67 The Impact of Fires Related to Personal Mobility Device and Power-Assisted Bicycle in Singapore

##### A. LIM^1^, J. KWEK^2^, L. ANG^3^, S. ARULANANDAM^4^, S. SHALI^5^

###### ^1^Operations Branch, 3^rd^ SCDF Division, Singapore Civil Defence Force, Singapore; ^2^Ang Mo Kio Fire Station, Singapore Civil Defence Force, Singapore; ^3^Fire Research Unit, Fire Safety and Shelter Department, Singapore Civil Defence Force, Singapore; ^4^Medical Department, Singapore Civil Defence Force, Singapore; ^5^Fire Investigation Unit, Operations Department, Singapore Civil Defence Force, Singapore

####### **Correspondence:** J. KWEK

**Background**

There has been an increased use of personal mobility devices (PMD) and power-assisted bicycles (PAB) in Singapore as alternative transportation modes. However, such devices carry fire risks. This study aims to investigate the impact of PMD/PAB-related fires to the health of the users, and to describe some notable cases attended by the Singapore Civil Defence Force (SCDF) Emergency Medical Services (EMS) over the 2 year-period in 2017 and 2018.

**Materials and Methods**

PMD/PAB-related fires attended by SCDF were identified through a retrospective review of fire investigation reports by SCDF Fire Research Unit (FRU). Where the fires resulted in injuries, the EMS patient records were studied. Variables studied include injury type, severity, and injured body region. Three notable cases resulting with partial thickness burns and classified as Patient Acuity Category 1 (PAC 1) are described in this study.

**Results**

Out of 123 cases of PMD/PAB-related fire incidents, 17 cases resulted in 44 injured persons, with 7 cases involved multiple casualties. 34 casualties were conveyed to the hospital, of which 7 (20.6%) were classified as PAC 1. 47.1% of the patients sustained only smoke inhalation injuries, and the remaining sustained burn injuries only or both. Most commonly, the upper limbs were injured. One notable case involved an adult patient who sustained smoke inhalation injury and 50% partial thickness burn over the head, face, torso and all limbs. Two paediatric patients sustained 40% and 7% partial thickness burn injuries respectively, affecting mostly the torso and limbs. In all cases, the patients were in the same room where the devices were being charged when they caught fire.

**Conclusions**

Although PMDs and PABs offer convenience and increased mobility, the fire risk, especially during the electrical charging process, carries significant impact on public health. Increased public education may be needed to manage the fire hazards related to such devices.

### Abstract Topic: Others

#### S68 The Use of ImageJ to Calculate Total Body Surface Area in Burn Patients: A Case Series

##### Aditya Wardhana, Rizta A. Widyana, Gammaditya A. Winarno, Sanjaya Faisal T, Raihana Daisy A

###### Division of Plastic, Reconstructive, and Aesthetic Surgery, Department of Surgery, Cipto Mangunkusumo Hospital Jakarta, Indonesia

####### **Correspondence:** Rizta A. Widyana

**Background**

Assessing the total body surface area (TBSA) of burn has been a matter of clinical judgment even though some modalities now serve as adjunct tools to increase accuracy. We compared the result between clinical judgment from an experienced burn surgeon (>10 years in the field) and manual calculations using ImageJ.

**Materials and Methods**

We conducted the study in the burn unit of Cipto Mangunkusomo Hospital, Jakarta. Using transparent dressing, we traced the border of each wound and the area is measured using “ROI Manager” in ImageJ. The overall BSA of each patient was calculated using the Dubois Formula as the denominator to determine the percentage of burn. The result from ImageJ calculation is then compared to the Rule of Nine.

**Results**

Data was collected in the first day post injury of 3 patients, all with partial to full-thickness burn. Patient A was a normoweight female with 36% burn clinically and 34.7% from ImageJ. Patient B was an obese female with 25% burn clinically and 32% from ImageJ. Patient C was an overweight female with 10% burn and 8.5% from ImageJ. In 2 patients, measurements are quite identical with <2% difference. The exception in 1 patient with 7% difference may be caused by obese proportion.

ImageJ provides detailed decimal number with a setback in circular body parts such as arms. Dressing was unable to attach precisely for accurate tracing. ImageJ is useful for non-circumferential wound or when an exact area of wound is needed for research.

**Conclusions**

ImageJ is an aid to calculate wound area but may not be reliable for burns with unpredictable and circumferential wound. An attempt to measure BSA using ImageJ, though not futile, is not more beneficial than using the common rule of nine.

### Abstract Topic: Others

#### S69 Reviving the Burn Scoring System: An Aid for Appropriate Patient’s Care in Cipto Mangunkusumo General Hospital, Jakarta, Indonesia

##### Aditya Wardhana, Sanjaya F Tanjunga, Rizta A Widyana, Gammaditya A Winarno

###### Division of Plastic, Reconstructive, and Aesthetic Surgery, Department of Surgery, CIpto Mangunkusumo Hospital Jakarta, Indonesia

####### **Correspondence:** Sanjaya F Tanjunga

**Background**

Predictive scoring systems for burns are available to predict patient’s prognosis. Yet the complexity of injury, demographic of patients, and hospital standards may result to the outcome not always linear to the supposed survival possibility.

**Materials and Methods**

In this retrospective cross-sectional study, we collected data from health record, a total of 513 patients between 2013-2017 and ran them into four scoring systems: Modified Baux, ABSI, BOBI, and Ryan Score. The final results are probability of mortality or survival of each patients and we compare them into the real results during inpatient treatment. Patients are classified as healed according to the discharge criteria in our hospital before being moved to outpatient treatment. The exclusion criteria for this study are children (<18 years old), patients admitted >7 days post injury, chronic burns, electrical and chemical burns.

**Results**

The cut-off value for ABSI is 8, Ryan is 1, BOBI is 4, and Modified Baux is 93. For the ABSI score, the AUC is 0.772 (CI 95%, 0.725-0.819) with p-value of 0.000. For the Ryan Score, the AUC is 0.593 (CI 95%, 0.537-0.649) with p-value of 0.001. For the BOBI score, the AUC is 0.582 (CI 95%, 0.527-0.638) with p-value of 0.003. For Modified Baux Score, the AUC is 0.755(CI 95%, 0.705-0.805) with p-value of 0.000.

**Conclusions**

All the scoring systems can be used in our unit based on the p-value but the best one is the ABSI score. Knowing this may be helpful to determine the standard of care for each patient to maximize the available resource.

### Abstract Topic: Others

#### S70 Thermal Imaging Device as an Aid to Determine Burn Wound Depth: a Case Series

##### Dhita Kurniasari, Akhmad Noviandi Syarif, R Aditya Wardhana

###### Plastic Reconstructive and Aesthetic Surgery Division, Department of Surgery, Cipto Mangunkusumo Hospital, Medical Faculty, Universitas Indonesia

####### **Correspondence:** Dhita Kurniasari

**Background**

Burn injury is considered as one of major problem because of high mortality and morbidity, not only due to improper treatment of burn resuscitation during the first 24 hours, but also due to the extent and depth of burns itself. The right depth of wound determines the next action whether the burn will be excised or not. Determining the depth of burns clinically is a learning curve and less accurate because it depends on the experience of the surgeon. A method is needed to make clinical assessment more objective in determining burn wound depth.

**Materials and Methods**

We did clinical and thermography assessment using Flir One® thermal imager to determine the conversion of burn depth of patient before and after the surgery. Subsequently, temperature differences between the burn wound and healthy skin (ΔT) were measure and compared before and after the excision.

**Results**

We performed clinical and thermography assessment in 3 patients, 2 male and 1 female patient, with age range 25-48 years old, and with burn size 7.5% - 41% TBSA. Two patients showed low temperature (temperature of burn area: 34.2°C, ΔT: -2.2°C) on the area of the burn wound so we decided to excised it. After the surgery it shows that the ΔT narrower (temperature of burn area: 32.7°C, ΔT: -0.5°C), that indicated the improvement of the circulation. One patient showed an area of burns with an average temperature (temperature of burn area: 36.9°C, ΔT: 2.2°C). We decided not to excise and appear epithelialization on the 7th day post burn.

**Conclusions**

Thermography assessment such as Flir One® thermal imager can be utilized to determine the depth of burns in addition to clinical assessment. It benefits not only for the functional purpose, but also affordable price and practically used. In the future it may be useful to help both experienced surgeons and novices.

### Abstract Topic: Others

#### S71 Establishment and effects of a method for wound repair in patients with extensive deep burns using fresh skin allografts and autologous micrograft

##### Chuanan Shen

###### Department of Burns and Plastic Surgery, The Fourth Medical Center of Chinese People’s Liberation Army General Hospital, Beijing, China

**Background**

Skin allografting is an effective way to cover the wound in extensive burns. However, cadaveric skin has been scarce in recent years in China. This study tried to establish a method for wound repair in patients with extensive deep burns using fresh allogeneic scalp and autologous micrograft.

**Materials and Methods**

Two patients with burn injuries involving 90% (3rd degree, 70%) and 97% (3rd degree, 85%) total body surface area (TBSA) respectively were treated with fresh scalp allografts donated by 32 males aged (31.5 ± 8.2) years or autologous micrografting. The bilateral extremities with third-degree burns were selected as treatment and control groups. Wounds in the treatment group were treated with fresh allogeneic scalp and autologous micrograft, while wounds in the control group received MEEK grafting. Preoperatively, the surgical area on the extremities was calculated to estimate the necessary amount of allogeneic scalp and MEEK grafts. Fresh scalps (0.30 - 0.35 mm) were harvested from each donor to prepare a larger piece of skin allograft. Autologous micrografts were transported onto the epidermis of the skin allograft. The treatment and control group received grafting according to our protocol. The donors received follow-up visits after 3 months to see if there is alopecia and scar hypertrophy. The wound coverage rate was observed in both treatment and control groups on postoperative weeks 2, 3, 4 and 5.

**Results**

The donor sites in all allogeneic skin donors healed within 10 days postoperatively. The scalp recovered well without any alopecia or scar hypertrophy during the follow-up visits. The wound coverage rate of the treatment group was approximate to or higher than that of the control group.

**Conclusions**

Since allogeneic skin is scarce and expensive and the patient’s relatives are willing to help save the patient’s life by donating the scalp, this method may be a feasible option in clinical practice.

### Abstract Topic: Paediatric/Electrical & Chemical Burns

#### S72 Acticoat Dressings In The Management Of Partial Thickness Paediatric Burns : Our Experience

##### Vaibhav Bhisikar, Seema Rekha Devi

###### Plastic surgery department, Gauhati Medical College & Hospital, Guwahati, Assam, India

####### **Correspondence:** Vaibhav Bhisikar

**Background**

Burns in the paediatric patients are accidental household burns due to spillage of hot liquids & not child abuse.Small houses and open kitchen also contribute.20 % of all burn patients treated in our burn unit were children .These are usually partial thickness burns .In the present study efficacy of Acticoat dressing in paediatric burns was analysed.

**Materials and Methods**

30 children (6months - 16 years) with clean , upto 20 % total body surface area (TBSA) partial thickness burns who met the inclusion criteria were included in a prospective study from september 2018 to february 2019. The children received Acticoat derssing. Patients were analysed in terms of age and sex of patient,percentage of burns, duration of stay and complications.Measures of burn re-epithelialization,pain were recorded until full re-epithelialization occurred.

**Results**

Mean age of presentation was 8 years ,14 males 16 females.Average hospital stay was 11 days , reduced dressing change frequency, reduced need for pain medication during dressing change , reduced infection rate and reduced average healing days.

**Conclusions**

Acticoat dressing is well tolerated because of ease of dressing application and removal. It is effective in terms of wound epithelialization. It reduces the pain during dressing change and hospital stay for clean <20 TBSA partial thickness burns. Reduces need for surgical intervention.

### Abstract Topic: Paediatric/Electrical & Chemical Burns

#### S73 The management of burn wounds in pediatric patients

##### Yan liu, Yan Shi,Jian Zhang, Wenkui Wang

###### Department of Burns and Plastic Surgery, Ruijin Hospital Affiliated to Shanghai JiaoTong University School of Medicine, Shanghai, China

####### **Correspondence:** Yan liu

**Background**

More than 6000 pediatric patients were treated in our burn center every year. The management of their burns wounds remains a challenge for us

**Materials and Methods**

We collected data from pediatric inpatients under the age of 14 years in 2018 and analyzed the demographics data and wound management methods.

**Results**

In 2018, more than 6000 pediatric patients were treated in our burn center. Among total of 314 inpatients, 178 received scar reduction surgery and 136 were acute burn patients. The most common mechanism of injury was scalds (120/136), followed by flame (13/136) and electrical injury(2/136). The average burn area of inpatients was 12.32±9.66%TBSA, and the average III degree burn area was7.96 ±8.95%TBSA . Among all pediatric acute burn patients, 97 of them received surgery; each patient had 1.5 surgeries in average. Among the pediatric patients with acute burn wounds but did not receive surgery, a large amount of their wounds were covered with various of wound dressing, including alginate dressing, nanometer silver or silver ions dressing, hydrocolloid dressings, alone or combination of two dressings. The treatments have achieved satisfying outcomes, in regard of healing time and the quality of healed wound.

**Conclusions**

Non-surgical treatment remains the principal treatment for burn pediatric patients. Based on the characteristics of wounds, proper wound dressing may accelerate wound healing and, to some extent, avoid surgical risks

### Abstract Topic: Paediatric/Electrical & Chemical Burns

#### S74 Analysis of 6 Cases of Young Children with Facial-Neck Scald and Upper Airway Injury

##### YI JI

###### Plastic and Burn Surgery Department, The Children’s Hospital Of Nanjing Medical University, Nanjing, Jiangsu, China

**Background**

The purpose of this review is to emphasize timely treatment for the scald of facial-neck regions with young children, it is recommended to use laryngoscopy to detect upper respiratory injury which can bring breathing difficulties, to avoid suffocation, respiratory cardiac arrest.

**Materials and Methods**

Reviewed six children (from 9 months to 18 months) of different degree scalds with facial-neck at our hospital in 2017, five patients between 12 and 24 hours after admission had difficulty in breathing, oxygen desaturation. So with general anesthesia (intravenous ketamine 2 mg/kg), endotracheal intubation with trachea cannula size from 3.5 to 4.5 mm. Keeping the tube time was from 5 ~ 8 d, 6.5 d on average. One child had respiratory and cardiac arrest on the way to hospital, and he was given mechanical ventilation after cardiopulmonary resuscitation after admission. Indications of tube withdrawal: regression of face and neck edema, no severe lung infection, significantly reduction of airway secretions and children were able to cough spontaneously.

**Results**

All the 5 cases were cured without serious lung infection, peptic ulcer and bleeding, and the wound was healed well. The child with cardiac and respiratory arrest before admission was requested by his parents to transferred to another hospital for treatment after 21 days of mechanical ventilation.

**Conclusions**

If it is sure that the upper airway has damaged by hot liquid, endotracheal intubation must be performed immediately. So, for children with scald close to the mouth and neck, the laryngoscope can be used as a routine examination.

### Abstract Topic: Paediatric/Electrical & Chemical Burns

#### S75 Epidemiology of pediatric burns injuries in Singapore: A pilot study

##### Azman N, Goh JM, Cheng SHJJ, Hsieh MKH

###### Department of Plastic, Reconstructive & Aesthetic Surgery, KK Women’s & Children’s Hospital, Singapore

####### **Correspondence:** Azman N

**Background**

KK Women’s and Children’s Hospital is the referring pediatric hospital in Singapore for a wide variation of pediatric burns and to date, no epidemiological review was done for pediatric burn injury in Singapore. In 2017, our burns service at KK Women’s and Children’s Hospital provide inpatient and outpatient treatment for 373 pediatric patients.

**Materials and Methods**

Our database was accessed to collate a retrospective data of all new cases managed by our Burns service from January 2018 to December 2018 at KK Women’s and Children’s Hospital .

**Results**

89% of the patients were treated as outpatient, while 11% were inpatient. The most common mechanism of injury is scalding. Most of the pediatric patient had a TBSA of less than 5%.

**Conclusions**

The large number of burns injury in children prompted our department to collect comprehensive information of burn data. These data can be used to establish new treatments, initiatives and programs.

### Abstract Topic: Paediatric/Electrical & Chemical Burns

#### S76 Pediatric Burn injuries related to electric bike or electric scooter

##### Azman N, Goh JM, Cheng SHJJ, Hsieh MKH

###### Department of Plastic, Reconstructive & Aesthetic Surgery, KK Women’s & Children’s Hospital, Singapore

####### **Correspondence:** Azman N

**Background**

In 2018, Singapore Civil Defense Force (SCDF) reported 74 fire cases, involving 50 electric scooters and 22 electric bikes. The number of burn injuries related to electric bike or electric scooter is an emerging problem in Singapore and the number of burn injuries involving these mobility devices is on a rise.

**Materials and Methods**

Our database was accessed to retrieve a retrospective data of all burn injuries related to electric scooter or electric bike.

**Results**

9 burn injuries related to electric scooters or electric bikes were retrieved from our database. 7 of these burn injuries were primarily caused by the explosion of the batteries.

**Conclusions**

Electric scooter or electric bike related burn injuries are an emerging mechanism of burn injury in Singapore. Severity of burns ranges from 0.5% to 40% TBSA without inhalational component. The primary source of fire is related to the battery installed in these mobility devices. This novel form of thermal ignition burns needs to be highlighted for the prevention of future burns given the landscape and uprising of personal mobility devices in Singapore

### Abstract Topic: Paediatrics Wound Management

#### S77 The efficacy of prophylactical probiotics administration in children with severe burns: a retrospective study

##### Zhiyuan Shi, Xianbo Ye, Minhui Zhu, Ming Zhang

###### Burn & Plastic Surgery, The Sixth Medical Center of PLA General Hospital, Beijing, China

####### **Correspondence:** Zhiyuan Shi

**Background**

Intestinal disorder is not rare in pediatric burn patients. This retrospective study was intended to evaluate the effect of prophylactical probiotics administration in these patients.

**Materials and Methods**

Patients with acute burns affecting >30% of total body surface area, deep areas >10% of TBSA, were enrolled. Between May 2012 and September 2017, the datum of 31 pediatric patients, aged between 2- 8 years, were screened out. Probiotics were administrated in 9 patients before intestinal disorder occurred (Group A), and 22 patients after diarrhea (Group B). Clinical datum (infection, antibiotic, operative times, intestinal disorder, and mortality) were compared between two groups. Length of stay was modified with burn size.

**Results**

There were no differences in age (5.3±1.8; 6.2±2.1), burn size (45.0±10.3; 40±9.8), operative times (2.5±0.7; 2.8±0.9) and intestinal disorders incidence (3/9, 9/22, p>0,05). The diarrhea periods were 2.7±2.3 days in group A, which shorter than those in group B 5.3±3.7 days (p<0.05). The ratio inversion of cocci and bacilli were detected in 4 and 12 patients in each group. The metronidazole was administrated in 5 patients in group B. Flatulence was observed in 6 and 20 patients in each group. The time required to complete wound healing was not significantly different between group.

**Conclusions**

Prophylactical probiotics administration could shorten the periods of diarrhea without reducing the incidence of intestinal disorders.

### Abstract Topic: Paediatrics Wound Management

#### S78 Surgical treatment of hand treadmill injuries

##### Yanni Wang, Hongyan Qi

###### Department of Burn and Plastic Surgery, Beijing Children's Hospital, Capital Medical University, Beijing, China

####### **Correspondence:** Yanni Wang

**Background**

To summarize the clinical effect of repairing hand injury tissue defect caused by running machine.

**Materials and Methods**

From July 2012 to January 2019, 28 cases of hand soft tissue defect caused by treadmill were treated. According to the condition of hand soft tissue defect, groin full thickness skin graft (10 cases), VSD + split-thickness skin graft (3 cases), artificial dermis + autologous skin (3 cases), abdominal pedicled skin flap (8 cases), finger palmar advancement skin flap (1 case), V- Y skin flap were used to repair the defect.

**Results**

28 cases of skin wounds survived. The follow-up period was at least 6 months and the longest was more than 1 year. Of them, 25 cases had satisfactory shape and function recovery, 3 cases had slight swelling of skin flaps and 3 cases had second-stage operation.

**Conclusions**

Appropriate methods for repairing soft tissue defects after hand trauma caused by running machine according to different soft tissue defects have the advantages of low infection rate, short hospitalization time and better recovery of hand shape.

Cheng Guoliang, the role of foot flap in thumb and finger reconstruction and repair. Chinese Journal of Microsurgery, 2005, 28:291-294

Wang Shuhuan, Hand Surgery, 2nd Edition, Beijing: People's Health Publishing House, 1999.181-194

### Abstract Topic: Paediatrics Wound Management

#### S79 Clinical observation of micropower negative pressure wound therapy in pediatric deep II degree burn wound

##### Xiaopeng Zheng^1,2^, Xiaoyan Hu^1^, Tiansheng Chen^1^, Yaonan Jiang^1^, Xiangyang Xu^2^, Lie Zhu^3^, Shichu Xiao^1^, Zhaofan Xia^1^

###### ^1^Burn and trauma center, Changhai Hospital, Second Military Medical University, Shanghai, China; ^2^Department of burn and plastic surgery, The 413th Hospital of PLA, Zhoushan, Zhejiang Province, China; ^3^Department of plastic surgery, Changzheng Hospital, Second Military Medical University, Shanghai, China

####### **Correspondence:** Zhaofan Xia

**Background**

To observe the clinical curative effect of the application of micropower negative pressure wound therapy (mNPWT) in pediatric deep II degree burn wound.

**Materials and Methods**

48 cases of pediatric burn patients were selected who were admitted in Changhai Hospital of Shanghai from January 2014 to January 2015. They were randomly divided into treatment group (mNPWT) and control group (routine dressing change). The wound healing rate, the positive bacterial culture rate and the surgical rate were compared between the two groups. And all patients were followed up for 1 year to evaluate the formation of scar.

**Results**

2 and 3 weeks after injury, the wound healing rate in the treatment group ((49.69 ± 2.59)%, (95.85 ± 2.26)%) was significantly higher than that in the control group ((39.82 ± 2.56)%, (75.23 ± 2.02)%) (P < 0.01). While the positive bacterial culture rate in the treatment group (38.5%, 23.1%) was significantly lower than that in the control group (75.0%, 68.2%) (P < 0.01). After 3 weeks, the surgical ratio in the treatment group (7.7%) was significantly lower than that in the control group (36.4%) (P < 0.05). in the 3rd, 6th and 12th month after wound healing, the VSS (Vancouver Scar Scale) scores in the treatment group (3.85 ± 1.69, 3.08 ± 1.44, 2.85 ± 1.12) were significantly lower than that in the control group (4.86 ± 1.25, 4.14 ± 1.67, 3.73 ± 1.45) (P < 0.05).

**Conclusions**

The method of mNPWT in repairing pediatric deep II degree burn wound, compared with routine dressing change, can significantly improve the wound healing rate, decrease the wound infection and improve the quality of wound healing. Thus it is a new kind of simple and effective method.

### Abstract Topic: Paediatrics Wound Management

#### S80 Transplantation of fresh scalp allograft in repairing extensive deep burn wounds in children

##### Chuanan Shen

###### Department of Burns and Plastic Surgery, The Fourth Medical Center of Chinese People’s Liberation Army General Hospital, Beijing, China

**Background**

It is difficult to treat pediatric extensive burns, which contribute to high mortality rates, partly because of the lack of large allogeneic skin to close wound in China. Therefore, we innovatively used fresh scalp as thin split thickness skin allografts to cover the burn wounds of pediatric patients.

**Materials and Methods**

Fresh scalp allografts were harvested from voluntary donors who were patients’ relatives. The median total burn area in the major burns was 40% TBSA, ranging from deep-second to third-degree injuries. The fresh scalp allografts were transplanted on the wounds post tangential excision or escharectomy in the way of mere fresh scalp allografts coverage or mixed coverage with autografts and fresh scalp allografts.

**Results**

All the patients survived without serious complications during the treatment period. The median healing time was 47 days; and the average healing time of the donors’ scalps was (7.6±1.08) days with no scar formation, alopecia areata or folliculitis post operation.

**Conclusions**

The use of fresh scalp allografts in the treatment of pediatric major burns is an effective and feasible method in protecting wounds and promoting wound healing as well as in reducing scar formation in the donor sites of burned children. The high ratio of fresh scalp areas to pediatric burn wound areas ensures high efficiency of wound coverage; and healthy relative skin donors have more initiatives and favorable healing results.

### Abstract Topic: Critical Care/Resuscitation and Pharmacology

#### S81 Alteration of pharmacokinetics of imipenem in severe burn patients

##### Anh Quang Luong, Lam Nhu Nguyen

###### Vietnam National Burns Hospital, Military Medical University, Hanoi, Vietnam

####### **Correspondence:** Anh Quang Luong

**Background**

Burn is complex injury with high risk of hospital resistant organism infection. The use of broad spectrum antibiotics such as imipenemis common. Nevertheless, substantial change in physiopathology including augmented renal clearance (ARC) observed in severe burn patients results in high pharmacokineticvariability. Toxicity or sub-therapeutics may occur. This study aimed to estimate pharmacokinetic (PK) parameters of imipenem and those potential covariates.

**Materials and Methods**

Burn patients with body surface area injured > 20% and imipenem indication were recruited. Two set of plasma samples (30 min post-dose and 1-2 hours before next dose) were obtained at imipenem initiation and before the end of imipenem use. ARC was defined if 8h-urinary creatinine clearance (8hClcr) was above 130 mL.min-1173 m2. PK samples were quantified by validated HPLC method. Population pharmacokinetic analysis were performed using Monolix2016R1.

**Results**

A total of47 sets with 94 plasma samples were collected from 24 patients. Of which 18 sets were obtainedat ARC time. One compartmental model with proportional error fitted the data best. The inclusion of inter-individual (IIV) and inter-occasion variation (IOV) improved the goodness of fit of the model. Population volume of distribution was 33.5 L with IIV and IOV of 18.2 % and 15.6%, respectively. Population clearance and the respective IIV and IOV were 18.8L.h-1, 27.0 % and 28.1 %. Age and ARC showed to besignificant covariates (p<0.001). Targeted PK/PD attainment appeared to be affected as a consequent.

**Conclusions**

Imipenem pharmacokinetics had significant IIV and IOV on burn patient and the ARC may influence the targeted PK/PD attainment.

### Abstract Topic: Pharmacology/ Renal Replacement

#### S82 Effects of simvastatin on iNOS and caspase-3 levels and oxidative stress after smoke inhalation injury

##### Zheng Jun Cui^1^, Rong Qiang Yang^2^

###### ^1^Department of burn and repair reconstruction surgery The First Affiliated Hospital of Zhengzhou University Henan, People's Republic of China; ^2^Department of Plastic surgery Zhengzhou Central Hospital Affiliated to Zhengzhou University Henan, People's Republic of China.

####### **Correspondence:** Zheng Jun Cui

**Background**

The overexpression of iNOS induces cell apoptosis through various signal transduction pathways and aggravates lung injury. Caspase-3 is an important protein in the apoptotic pathway, and its activation can aggravate apoptosis. Simvastatin, a hydroxymethyl glutaryl-A (HMG-COA) reductase inhibitor, protects against smoke inhalation injury by inhibiting the generation and release of inflammatory factors and reduce the apoptosis of cells.

**Materials and Methods**

After Generation of an animal model of smoke inhalation injury, lung tissue and serum were taken at different time points, and the protein and mRNA expression of iNOS and caspase-3 in lung tissue, content of MDA and activity of SOD in lung tissue and serum were detected by different experimental methods. The results were statistically analyzed.

**Results**

Compared with the rats in the control group, pathological scores of lung tissue samples obtained from other four groups had significantly higher mRNA and protein expression levels of iNOS and caspase-3 (P<0.05), significantly lower SOD activity and a higher MDA content (P<0.05). Compared with the saline group, the low-, middle- and high-dose groups, had significantly lower pathological scores (P<0.05), significantly lower mRNA and protein expression levels of iNOS and caspase-3 and MDA contents in lung tissues (P<0.05), and significantly higher SOD activity in both lung tissues and serum and the same result was occurred when compared the low-dose group with the middle- and high-dose groups (P<0.05). With the exception of SOD activity in lung tissues at 24 and 72 h and MDA content in serum at 48 h, showed no significant differences between the middle- and high-dose groups.

**Conclusions**

In rats with smoke inhalation injury, simvastatin inhibits iNOS and caspase-3 expression in lung tissues and oxidative stress, and thereby exerts a protective effect, and the effect and dose are positively correlated within a definitive range.

### Abstract Topic: Pharmacology/ Renal Replacement

#### S83 A systematic review of nebulized heparin doses in adults with inhalation injury

##### Chuan Poh Lim, Xiu Yun Ang

###### Department of Pharmacy, Singapore General Hospital, Singapore

####### **Correspondence:** Chuan Poh Lim

**Background**

The aim of this review was to compare the efficacy and safety of different nebulized heparin doses used in adults with inhalation injury.

**Materials and Methods**

All potential studies were identified from four databases (PubMed, Embase, Scopus, Web of Science). All potential studies were screened using title and abstract by two independent reviewers. Full-text screening was done for identified studies that fulfilled the initial screening criteria. Studies that fulfilled the full-text screening were then selected for full-text review. Criteria for full-text review were studies that involved adults with inhalation injury and heparin via nebulization route. Main outcomes assessed were duration of mechanical ventilation, incidence of unplanned re-intubation, pneumonia and bleeding risk.

**Results**

Seven out of 176 potential studies were included in this review. Nebulized heparin doses used in these studies were 5000 (Hep5) or 10000 (Hep10) units given every 4 hours. There were four Hep5 studies, two Hep10 studies and one study comparing Hep5 with Hep10 (Hep5/Hep10). Two out of 6 studies reported significant reduction in the duration of mechanical ventilation. In the Hep5/Hep10 study, Hep10 group showed significantly shorter mechanical ventilation compared to Hep5 group. In the two Hep5 studies that reported incidence of unplanned intubation, there was no significance compared to control. Two out of 6 studies that looked into pneumonia incidence reported significance compared to control. There were 5 studies that reported bleeding complications, of which three Hep5, one Hep10 and one Hep5/Hep10 studies. No increased in bleeding complications were noted.

Overall quality of the studies was poor due to no control of confounders, no blinding, unclear treatment duration, poor documentation of missing data/loss to follow-up.

**Conclusions**

Our findings suggest both nebulized heparin doses may be beneficial and safe. Due to the poor study quality, a large well-designed prospective study will be needed to validate its use in adults with inhalation injury.

### Abstract Topic: Psycho-Social Care

#### S84 Using the ecological perspective and strengths perspective to develop psychosocial interventions in burns – The case of a female major burn client

##### Yu-Chieh Wu, Yuh-Jen Kuang

###### Taipei Rehabilitation Center, Sunshine Social Welfare Foundation, Taipei, Taiwan

####### **Correspondence:** Yu-Chieh Wu

**Background**

Physical problems following severe burns are often compounded by other intrinsic and extrinsic factors, which require comprehensive psychosocial interventions.

**Materials and Methods**

We present the case of a 30 year old female with 97% 2^nd^-3^rd^ degree burns, severe left hand contracture and right hand finger amputation. Absence of family support and income led to major self-care problems. Other issues included poor body image, emotional distress and adjustment difficulties affecting interpersonal relations and social participation. The ecological and strengths perspectives served as a theoretical framework to identify needs and develop psychosocial interventions.

**Results**

The client had major self-care problems and anxiety caused by the absence of family support and income, as well as a feeling of isolation due to her different appearance. Using the ecological perspective, which helps understand how the individual and the social systems mutually influence each other, interventions adopted include financial aid and short-term housing with daily care to ensure that rehabilitation continued uninterrupted, leveraging peer support in the environment of the rehabilitation center and short-term housing facility to address isolation, and designing social participation activities to help the client progressively learn how to meet people and accept her new self. Emotional distress of the client included powerlessness and hopelessness. Using the strengths perspective, which emphasizes identifying and using the person’s inner resources to address problems, the client was actively involved in the elaboration of her rehabilitation plan and within defined parameters, decided goals and interventions, thus regaining a sense of agency and seeing herself as someone with capabilities, which further strengthened her rehabilitation motivation.

**Conclusions**

The ecological and strengths perspectives are useful frameworks to examine issues affecting burn survivors from a macro/social perspective (as opposed to micro/individual), as well as identifying outside and inner resources which can be leveraged to support recovery.

Informed consent to publish has been obtained from this patient.

### Abstract Topic: Psycho-Social Care

#### S85 Avoidant coping strategy positively correlates with post-traumatic disorder and negatively correlates with post-traumatic growth and quality of life in patients with burns: a longitudinal study

##### Ting-Chia Wang^1,2^

###### ^1^Burn Rehabilitation and Post-Acute Care Center, New Taipei City Hospital, New Taipei City, Taiwan; ^2^Department of Psychiatry, New Taipei City Hospital, New Taipei City, Taiwan

**Background**

The aim of the study was to examine the relations among avoidant coping strategy, post-traumatic stress disorder (PTSD), post-traumatic growth (PTG) and quality of life (QoL) in burn survivors from a mass burn casualty event occurred in Taiwan.

**Materials and Methods**

77 burn survivors from the incident were enrolled. Questionnaires including Brief COPE scale and Impact Event Scale (IES) were administered at 6 months and 2 years after the incident. Post Traumatic Growth Inventory (PTGI), Burn Specific Health Scale (BSHS) were administered at 2 years after the incident. Pearson’s correlations were calculated for 3 dimensional indexes of Brief COPE, total scores and 2 dimensional indexes of IES, total scores and 5 dimensional indexes of PTGI and total scores of BSHS and other 9 indexes.

**Results**

The avoidant emotional coping index has significantly positive correlation with total score and other 2 indexes of IES at 6 months (*r*= 0.50, 0.44, and 0.52, *p*<0.01) and 2 years (*r*= 0.53, 0.51, and 0.65, *p*<0.01). On the other hand, avoidant emotional coping index has significantly negative correlation with total score and other 5 indexes of PTGI (*r*= -0.35, -0.32, -0.41, -0.31, -0.34 and -0.31, *p*<0.05) and significantly negative correlate with total score and other 6 indexes of BSHS (*r*= -0.40, -0.33, -0.32, -0.49, -0.42, -0.40 and -0.55, *p*<0.05).

**Conclusions**

Burn survivors who used avoidant emotional coping strategies tended to develop more severe PTSD symptoms since 6 months after accident and it might last for 2 years. And these survivors tended to have lower level of PTG and lower satisfaction of QoL. Therefore, Early identifications of using avoidant coping strategies might be important to prevent PTSD and improve PTG and QoL.

### Abstract Topic: Psycho-Social Care

#### S86 Quality of life and performance of return to work in recovered burn survivors: association with anxiety and depression

##### Ting-Chia Wang^1,2^, Yun-Yi Lin^3^, Te-Yu Gu^1^, Zhi-Hao Su^1^, Pei-Shan Liu^1^

###### ^1^Burn Rehabilitation and Post-Acute Care Center, New Taipei City Hospital, New Taipei City, Taiwan; ^2^Department of Psychiatry, New Taipei City Hospital, New Taipei City, Taiwan; ^3^Department of Physical Medicine and Rehabilitation, National Taiwan University Hospital, Taipei, Taiwan

####### **Correspondence:** Ting-Chia Wang

**Background**

The aim of the study was to examine the relations among anxiety, depression, quality of life (QoL) and performance of Return to Work (RTW) in burn survivors from a mass burn casualty event occurred in Taiwan.

**Materials and Methods**

77 burn survivors from the incident were enrolled. Questionnaires including Hospital Anxiety and Depression Scale (HADS) and Burn Specific Health Scale (BSHS) were administered at about 2 years after the incident. Pearson’s correlations were calculated for 3 indexes of HADS (total score, severity of anxiety (SA), severity of depression (SD)) and 2 indexes of BSHS (total score and work satisfaction).

**Results**

The total score of BSHS has significant negative correlations with total score, SA and SD index of HADS (*r*= -0.46, -0.44 and -0.42, *p*<0.01). The work satisfaction index of BSHS has significant negative correlations with total scores, SA and SD index of HADS (*r*= -0.44, -0.38 and -0.45, *p*<0.01).

**Conclusions**

Among the burn survivors from this mass burn casualty event, those who has more severe anxiety and depressive symptoms tend to have lower satisfaction of QoL and RTW. Early psychological interventions in reducing severity of anxiety and depression might improve QoL and RTW of burn survivors.

### Abstract Topic: Reconstruction

#### S87 The optimal functional and aesthetic results of Burn Hand reconstruction after Formosa Dust explosion - Total Hand reconstruction with one skin piece in one stage reconstruction

##### Shiow Shuh Chuang^1^, Yen Chang Hsiao^1^, Shu Yin Chang^1^, Hung Chang Chen^1^, Shih Yi Yang^1^, Katie Pei-Hsuan Wu^2^

###### ^1^Burn Center, Department of Plastic Surgery and Reconstruction, Linkou Chang Gung Memorial Hospital, Taoyuan, Taiwan; ^2^Department of Physical Medicine and Rehabilitation, Linkou Chang Gung Memorial Hospital, Taoyuan, Taiwan

####### **Correspondence:** Shiow Shuh Chuang

**Background**

Disaster of Formosa dust explosive, the average injury of TBSA is 44%. The reconstruction of the burn hands is a key challenge task for plastic surgeon. The goal of our work is to reconstruction the extremities in less procedure, to obtain the maximum of function and aesthetics outcome [1]. Like to the face exposure to outside of clothing, in these areas, psychological problems produced by scar deformities are not less than the functional defect [2].

**Materials and Methods**

75 hands of 40 patients were undertook the reconstruction in CGMH in the past 3 years.

The standard protocol of the hand reconstruction:

1. Visual inspection and assess the hand scar contracture, and X-Ray referent to judge the condition of joints.

2. Skin resurface with FTSG and close in primary.

3. Excide the scar at hand dorsum, to excide the contractive scar over the joint, proximal side is always prior to the distal side.[3] [4]

4. The web space were created as well in meanwhile.

**Results**

74 in 75 hands were performed with FTSG, 70 hands performed in One stage operation, 69 in 75 performed with One piece skin reconstruction at dorsal side. 39 in 74 disfiguration finger were undertaken hemisection tendon lengthening.

**Conclusions**

The FTSG grafting could provide wide range of reconstruction and results with optimal functional and aesthetic satisfaction.

### Abstract Topic: Reconstruction

#### S88 Patient–physician shared decision making in reconstructive burn surgery

##### Yaw-Sheng Lin^1^, Lei-Peng Ng^2^

###### ^1^Department of Psychology, National Taiwan University, Taipei, Taiwan; ^2^R&D Department, Sunshine Social Welfare Foundation, Taipei, Taiwan

####### **Correspondence:** Lei-Peng Ng

**Background**

Shared decision making is a key component of patient-centered health care. This study investigated shared decision making in reconstructive burn surgery to describe the process and identify the perspectives of physicians and patients.

**Materials and Methods**

In-depth interviews were conducted from July to September 2018 with 4 burn physicians and 4 burn survivors with TBSA ranging from 60% to 90%. Thematic analysis was used to analyze the data.

**Results**

The need for reconstructive surgery is normally raised by physicians, allied health professionals or patients themselves in response to problems that limit progress of rehabilitation. In the shared decision making process, (1) problems are identified and treatment options are proposed; (2) options are prioritized and selected; (3) final decision is made by the patient. Physicians tend to look at problems from a “disease” perspective, with clear treatment options for abnormalities of structures and functions that are backed by scientific evidence and clinical reasoning, while patients tend to look at problems from an “illness” perspective, with the subjective response of being unwell and its impact on daily life guiding their preferences as opposed to medical knowledge. Despite these different perspectives, physicians and patients share the values of emphasizing the functional purpose of surgery over the cosmetic purpose, as well as setting functional independence and return to society and work as the ultimate goal of surgery. Also, despite a knowledge deficit, patients will trust physician expertise on how best to address their preferences.

**Conclusions**

Based on these findings, we propose that tools be developed (ex: list of questions to clarify needs) and that allied health professionals play a role in preparing patients (ex: providing knowledge and patient education) to further bridge the gap between physicians’ “disease” perspective and patients’ “illness” perspective.

### Abstract Topic: Reconstruction

#### S89 Functional outcomes of hand after full thickness burn of upper limb.

##### Ayesha Hanna, Mohd Zakir Hossain, Md Shoeb Ur Rashid

###### Department of Plastic surgery, Sir Salimullah Medical College & Mitford Hospital, Dhaka, Bangladesh.

####### **Correspondence:** Ayesha Hanna

**Background**

The hand, forearm and arm are a physiological kinetic unit jointed together functionally, biologically and mechanically from the fingertips to the shoulder. Full thickness burn injuries at any area of upper limb may affect hand functions. Fourth degree burn injuries involving tendons, joint capsules or bone are considered uncommon. Loss of hand functions negatively impact on occupation, activities of living & social interaction.

**Materials and Methods**

Objective: This study was conducted to measure hand functional performance after salvageable upper limb following full thickness burn.

Methods: Prospective observational study was conducted in the department of plastic surgery, SSMC & MH, Dhaka. Volunteer sample of burn survivors with salvageable upper limb following full thickness burn from June 2016 to June 2018 with one year follow up were included in the study with some inclusion & exclusion criteria. Hand functions were assessed by seven objective test criteria described BY Jebson.

**Results**

Total number of 13 burned upper limb in 10 patients were included in the study with a mean age 23.9 years, mechanism of injury was flame, electric and machinery burn. All patients required surgical intervention. 7 needed different types of flap coverage.6 needed split thickness skin graft. Physiotherapy & splintage were provided to the patient (for 6 months to 12 months) A long term physiotherapy was provided to all patients, then they achieved the specific goals with limitation despite residual deformities.

**Conclusions**

Basic trauma principles are followed. If escharotomies, fasciotomies were completed without delay if indicated. Elevation of the affected limb was maintained. The study was limited to patients presenting to our burn center include 3^rd^ degree & 4^th^ degree burn , did not include gangrene of finger, hands & different parts of upper limb & residual deformities.

### Abstract Topic: Reconstruction

#### S90 Release of web contracture of hand: V-M plus side graft VS webgraft

##### D. K. Seo

###### Department of Plastic and Reconstructive Surgery, Hangang Sacred Heart Hospital, Hallym University, Seoul, Korea

**Background**

Burn scar contracture of fingers always combine the webbing. To correct this problem, surgeons do the scar release and the full-thickness skin graft. Someone do the V-M plasty at the web and graft to the finger sides, and someone do whole graft at the web. Graft to the web is a complicated work, so surgeons often exhausted, also hematoma develop frequently than fingers. So V-M plus side graft seems attractive, but in most cases,, the whole web graft is more effective to release the contracture. The purpose of this study is to compare clinical outcomes of contracture treatment between the V-M plus side graft and the whole webgraft.

**Materials and Methods**

We analyzed the retrospective clinical and photographic records of 20 patients with burn scar contracture with webbing. We performed 10 cases of V-M plasty and full-thickness skin graft at the finger sides and 10 cases of whole web and finger graft.

**Results**

The overall take rate was about 95%, and focal graft loss was developed at the web graft cases. Mean follow up was 7 years. And the V-M plus side graft group show more early recontracture than web graft group especially in hard scar

**Conclusions**

The V-M c side graft looks good immediate postoperative period. And in the soft scar, it is true and the long term results was good. But in the hard scar, we think the whole web graft is better in view of recontracture and long term results.

### Abstract Topic: Reconstruction

#### S91 Reconstructive experience of multiple joint scar contracture deformity of limb in the simultaneous operation

##### Pihong Zhang, Licheng Ren, Jie Zhou, Pengfei Liang

###### Department of Burn Reconstructive Surgery, Xiangya Hospital, Central South University, Changsha, Hunan, China

####### **Correspondence:** Pihong Zhang

**Background**

To investigate reconstructive means of multiple joint scar contracture deformity of limbs in the simultaneous operation.

**Materials and Methods**

From January 2010 to June 2018, the team performed simultaneous surgical repair on 24 patients of scar contracture deformity at multiple joints of the same limb, including 15 cases of upper limbs and 9 cases of lower limbs. After the release of various joint scar contracture, autologous medium-thickness or full-thickness skin graft were used in 11 cases, skin graft combined with local skin flap in 9 cases, allogeneic acellular dermal matrix and autologous thin skin graft in 4 cases. Comprehensive rehabilitation treatment was taken after the operation.

**Results**

All flaps and skin grafts survived after operation in 22 patients. Two cases of partial skin necrosis occurred, of which one healed after a secondary skin graft, and the other healed after dressing changes. The deformity of scar contracture at each repaired joint was completely or basically corrected. 24 cases received 6-72 months’ follow-up after treatment, in addition, the range of motion of joints is basically normal. The upper limbs function of 15 cases was excellent in 12 cases and good in 3 cases. In the 9 cases of lower limbs, except one case of recurrence of scar contracture on the medial side of the knee, all joints functions were basically restored to normal with satisfactory results.

**Conclusions**

For continuous scar contracture deformity of multiple joints of the same limb, simultaneous surgical release and skin graft can reduce operation frequency and obtain better efficacy of surgical operation.

### Abstract Topic: Reconstruction

#### S92 Comparison of the effect of superficial iliac circumflex skin flap and medial plantar skin flap in the repair of thumb annular electric burn

##### Haiping Di, Chengde Xia, Peipeng Xing, Jidong Xue, Haina Guo, Lei Liu, Dayong Cao

###### Department of burns surgery, The first people’s hospital of Zhengzhou City, Zhengzhou, China

####### **Correspondence:** Chengde Xia

**Background**

The fingers play an important role in the body, especially the thumb which accounts for 50% of the function of the hand. The aim of this study was to compare the clinical effects of superficial circumflex iliac skin flap and medial plantar skin flap in the repair of thumb annular electric burn.

**Materials and Methods**

The clinical data of 30 patients with thumb ring electric burn admitted to our hospital from June 2015 to September 2017 were retrospectively analyzed. Among them, 15 patients with superficial circumflex iliac skin flaps were included in Group A and 15 patients with medial plantar skin flaps were included in Group B. The survival rate, wound healing rate, skin appearance, thumb function and sensation were compared between the two groups.

**Results**

The survival rate of superficial circumflex iliac skin flaps in both groups was 100.00%. The primary healing rate of wounds in group A and group B was 96.65%and 100%, respectively. There was no significant difference (P>0.05). In group A and group B, the excellent rate of skin flap appearance and finger function were 93.33% and 100.00%, 80.00% and 93.33% respectively 6 months after operation. There was no significant difference in the excellent rate of skin flap appearance and finger function between the two groups (P>0.05). The sensory function of skin flaps in group B was significantly better than that in group A at 6 months after operation (P>0.05).

**Conclusions**

The survival rate of superficial circumflex iliac skin flap and medial plantar skin flap for repair of thumb annular electric burn skin flap, the excellent appearance rate of skin flap and the excellent function rate of finger are similar, and the sensory function of the latter is better than the former.

### Abstract Topic: Others

#### S93 The effect of extracorporeal shock wave therapy on the grip strength of adult hand skin grafting for burns

##### Wu Weiwei, XU Xiaochuan

###### Department of Burn Surgery, The First Hospital of Jilin University, Changchun city ,Jilin provinces, China

####### **Correspondence:** Wu Weiwei

**Background**

To observe the effect of extracorporeal shock wave therapy on the improvement of grip strength for the adult hand skin grafting for burns.

**Materials and Methods**

There are 60 cases with the third degree burn of hand are selected who are treated after the skin grafting in the rehabilitation center treatment from May 2017 to November 2018. And there are equally divided into the control group and the observation group by the random digital table method. When the hand skin graft is fully alive, the control group is given conventional joint stretching and exercise therapy, and the observation group is additionally increased extracorporeal shock wave therapy. Then, the Jamar hydraulic meter is used to measure the grip strength after three weeks.

**Results**

The grip strength of the observation group is significantly higher than that of the control group (P<0.05).

**Conclusions**

Extracorporeal shock wave therapy is beneficial to improve the grip strength after the hand skin grafting for burns and is worth promoting.

### Abstract Topic: Reconstruction

#### S94 One-step approach for full-thickness skin defect reconstruction in rats using minced split-thickness skin grafts with Pelnac overlay

##### Tong Liu^1^, Chao Qiu^2^, Chi Ben^1^, Haihang Li^1^, Shihui Zhu^1^

###### ^1^Department of Burn Surgery, Institute of Burns, The First Affiliated Hospital, Naval Medical University, Shanghai 200433, China; ^2^Emergency Department, The First Affiliated Hospital, Naval Medical University, Shanghai 200433, China

####### **Correspondence:** Shihui Zhu

**Background**

Split-thickness skin grafting is the current gold standard for the treatment of traumatic skin loss. However, for patients with extensive burns, split-thickness skin grafting is limited by donor skin availability. Grafting split-thickness skin minced into micrografts increases the expansion ratio but may reduce wound repair quality. Dermal substitutes such as Pelnac can enhance the healing of full-thickness skin wounds, but their application currently requires two surgeries. The present study investigated whether it is possible to repair full-thickness skin defects and improve wound healing quality in a single surgery using Pelnac as an overlay of minced split-thickness skin grafts in a rat model.

**Materials and Methods**

A full-thickness skin defect model was established using Male Sprague Dawley rats (10 weeks old). The animals were randomly divided into control and experimental groups in which Vaseline gauze and Pelnac, respectively, were overlaid on minced split-thickness skin grafts to repair the defects. Wound healing rate and quality were compared between the two groups.

**Results**

We found that using Pelnac as an overlay for minced split-thickness skin grafts accelerated wound closure and stimulated cell proliferation and tissue angiogenesis. In addition, this approach enhanced collagen synthesis and increased the formation of basement membrane and dermis as well as the expression of growth factors related to wound healing while reducing scar formation.

**Conclusions**

Using minced split-thickness skin grafts overlaid with Pelnac enables the reconstruction of full-thickness skin defects in a single step and can increase the rate while improving the quality of wound healing.

### Abstract Topic: Reconstruction

#### S95 Anterior Neck Contracture Reconstruction Using Supercharged Skin Pedicled Flaps

##### Eimi Kodama, Yoshihiro Noda, Mio Tsuchiya, Rei Ogawa

###### Department of Plastic, Reconstructive and Aesthetic Surgery, Nippon Medical School Hospital, Tokyo, Japan

####### **Correspondence:** Eimi Kodama

**Background**

Reconstruction of the anterior neck requires attention to both aesthetic and functional outcomes. In general, skin grafts are not suitable for anterior neck reconstruction in Asian patients, even when artificial dermis is used prior to skin grafting. Employing various types of thin flaps, we found that skin pedicle must be sufficiently wide to achieve full release of neck contractures leading to neck extension. Here we describe two cases who presented with deteriorating neck contracture after extensive burns, treated by wide, thin, and long flaps harvested from the anterior chest wall.

**Materials and Methods**

The first patient was a 71-year-old man who sustained extensive burn injuries from a gas explosion 2 years before visiting our clinic. The second patient was a 19-year-old man who sustained extensive flamed burn injuries from a suicide attempt. Both cases underwent initial treatment including a split thickness skin graft (STSG) for their anterior neck. Before surgery, we confirmed the existence of internal mammary artery perforators (IMAPs) using Multi-Detector Computed Tomography (MDCT). The neck contractures were reconstructed by IMAP supercharged skin pedicled transposition flaps harvested from their anterior chest walls. IMAPs were anastomosed with facial arteries and veins. The contractures were released almost completely. Six months after surgery, the aesthetic results were also good.

**Conclusions**

Many methods for reconstructing neck contractures have been chosen: free flaps, local flaps, and free skin grafts. Along with obtaining good aesthetic outcomes, reconstruction of anterior neck scar contractions should also aim for full neck extension . In the present cases, we ensured almost complete release of the scar contracture and normalization of neck movement. Another advantage of relatively wide IMAP-based perforator-supercharged transposition flaps from the anterior chest is their high extendability which further alleviates tension on the flap edges. This reduces the risk of contracture recurrence and development of hypertrophic scars.

Informed consent to publish has been obtained from this patient.

### Abstract Topic: Scars Management and Pain Control

#### S96 The changes of immunoreactivity of mechanosensitive molecules upon pressure therapy

##### Yuting Zhang^1^, Cecilia Li^2^

###### ^1^Department of Rehabilitation Medicine, West China Hospital, Sichuan University, Chengdu, Sichuan, China; ^2^Department of Rehabilitation Sciences, Hong Kong Polytechnic University, Kowloon, Hong Kong SAR, China

####### **Correspondence:** Yuting Zhang

**Background**

Pressure therapy has long been the frontline conservative treatment of massive hypertrophic scars. Mechanotransduction was found to play a critical role in scar formation, while the effect of pressure therapy on mechanotransduction remains unknown. Therefore, a preliminary study was conducted to investigate the changes of immunoreactivity of mechanosensitive molecules upon pressure therapy.

**Materials and Methods**

A pretest-posttest was conducted to examine the clinical and histological manifestation of hypertrophic scars before and after 3-month standardized pressure therapy. Vancouver Scar Scale and ultrasound system were used to assess general scar condition and scar thickness. 3-mm scar biopsy was obtained from subjects, with analyzing the immunoreactivity of FAK, integrin- β1 and ERK in epidermis and dermis. Wilcoxon signed rank test and paired sample t test was used to compare VSS scores and scar thickness before and after pressure therapy. One-way repeated measure MANOVA was used to examined the influence of time on the change of the immunoreactivity of mechanosensitive molecules. Multiple regression analysis was used to contribution of post injury days and scar thickness on the change of immunoreactivity of mechanosensitive molecules.

**Results**

Thirty-one subjects with forty-three hypertrophic scars were enrolled. Scar thickness before (Mean=2.90, SD=1.28) and after (Mean=2.89, SD=1.36) pressure therapy did not show significance difference (t=0.05, p=0.96). Time had significant effect on the mean immunoreactivity of dermal integrin-β1 (F(1,39)=15.54, p<0.001), FAK (F(1,39)=7.51, p= .009), and ERK (F(1,39)= .636, p= .016). Post injury days negatively contributed to the variation in the change of immunoreactivity in dermal integrin-β1 and FAK.

**Conclusions**

Dermal immunoreactivity of mechanosensitive molecules was found to downregulated after 3-month pressure therapy, and time after injury is critical for treatment implementation.

### Abstract Topic: Scars Management and Pain Control

#### S97 Management of Burn Conversion : A Literature Review

##### Aditya Wardhana^1^, Adi Basuki^2^, Lady Aurora^3^, Jessica Marsigit^4^

###### ^1^Department of Plastic, Reconstructive, and Aesthetic Surgery, Department of Surgery, Faculty of Medicine Universitas Indonesia, Cipto Mangunkusumo National Hospital, Indonesia; ^2^General Practitioner, Biak General Hospital, Biak, Papua, Indonesia; ^3^Internship Doctor, Hermina Sukabumi Hospital, Sukabumi, West Java, Indonesia; ^4^Internship Doctor, Koja General Hospital, North Jakarta, Jakarta, Indonesia

####### **Correspondence:** Lady Aurora

**Background**

Burn conversion is the conversion of stasis zone into both greater burn area and burn depth. It may hamper the patients’ condition since morbidity and mortality are expected to be higher with the increase of burn size. To gain a comprehensive perception about the burn conversion, this study aims to collect the latest update about its therapy, diagnosis, etiology, and prognosis.

**Materials and Methods**

Literature searching was done on online databases, which were PubMed, SCOPUS, and PROQUEST. The keywords “Burns AND (Conversion OR Progression OR Expansion)” was formulated. The inclusion and exclusion criteria were applied.

**Results**

Twenty-six articles were found, which were divided into diagnosis of burn conversion (11%), aetiology of burn conversion (8%), prognosis of burn conversion (0%), and therapy of burn conversion (81%). All of the research was performed on animals. One of the best tools to diagnose burn conversion was the forward-looking infrared imaging (FLIR), having sensitivity up to 96% and specificity up to 100% to predict scar depth *≥*3 mm. The main aetiology was ischemia, reactive oxygen species and inflammation. Most of the research regarding the therapy showed benefit on preventing burn conversion. However, there were no side effect investigated and not all of the research was statistically significant.

**Conclusions**

More prognosis research and the treatment’s side effect about burn conversion should be conducted. Further research, which found effective, statistically significant, and safe, should be tested in human trial, since the animal trial and human trial may differ.

### Abstract Topic: Scars Management and Pain Control

#### S98 Signal Transduction Mechanisms of Renin-Angiotensin System Promoting Formation of Keloid

##### Junjie Chen, Ying Cen

###### Department of Burns and Plastic Surgery, West China Hospital, Sichuan University, Chengdu, Sichuan, China

####### **Correspondence:** Ying Cen

**Background**

As one kind of pathological healing result from skin trauma, keloid becomes a difficult task to dermatology, plastic surgery and even all of the medicine. Its etiology remains unclear. And none of treatment to keloid is consistently effective. Recent studies have found that the local renin-angiotensin system (RAS) could play an important role in many fibrosis diseases. Based on the above studies and our previous investigative results, this project attempted to explore the mechanism of RAS influencing the signal conduction pathway of ERK and JNK in keloid fibroblast cells, and elucidate the mechanism that AngII may promote synthesizing ECM and secreting fibrosis factors of keloid fibroblast cells.

**Materials and Methods**

1. The immunohistochemistry and Western blotting were used to detect the protein expression of JNK、pJNK、ERK、pERK and collagen;

2. Fibroblasts were freshly isolated from normal skin and keloid and were cultured.

3. The PD98059 and SP600125 were adopted respectively to block ERK and JNK pathway.

**Results**

The results of the experiment demonstrated: ①The expression of JNK、pJNK、 ERKand pERK were high in tissues and cells of keloid. ②By AngII interfering keloid fibroblast cells, their expression were obviously increasing, and the increasing expression could be effectively suppressed by selective angiotensin receptor antagonist. ③The results of further experiment of specifically blocking ERK or JNK signal conduction pathway demonstrated that the effect of AngII promoting collagen synthesis by ERK or JNK could be suppressed at certain degree.

**Conclusions**

The above results could show that the ERK and JNK signal conduction pathways were important in the RAS promoting keloid formation. The results of our investigation could help us institute the new therapeutic regimen of keloid aiming to AngII and serial signal conduction pathways.

### Abstract Topic: Scars Management and Pain Control

#### S99 The aesthetic effect of the repair of face and neck scar contracture with expanded frontotemporal flap

##### Chengde Xia, Jidong Xue, Haiping Di, Dayong Cao

###### Department of Burns,The First People's Hospital of Zhengzhou, Henan province, China

####### **Correspondence:** Chengde Xia

**Background**

To explore aesthetic effect of the repair of face-neck scar contracture deformity with expanded frontotemporal posterior axis flap

**Materials and Methods**

From January 2013 to June 2018, twelve male patients with severe face-neck scar proliferation and contracture deformity, which were distributed on the front ear, cheek, chin and neck were repaired by expanded frontotemporal flap with bilateral superficial temporal arteriovenous pedicle, which was formed by the method of frontotemporal scalp simultaneous expansion. The operation consisted of 3 stages:In stageI,one 400 - 600ml cylindrical dilator was implanted in frontal parietal, and two 100ml cylindrical dilator were respectively implanted in Bilateral temporal region. In stageII, the expanded flap with bilateral superficial temporal vessels was dissected to repair the wound after scar resection and contracture release. The neck curve was reshaped and the donor site was closed with saline. In stage III, flap pedicles were divided and pruned

**Results**

Seven patients underwent the operation of expanded frontotemporal flap transplantation and cervical skin dilatation. The duration of dilation was 4-6 months. Flap sizes ranged from 38 cm×9 cm - 45 cm×15cm. All flaps survived with healing of wounds. 12 patients were followed up for 4 -20 months, and the shape of the flaps were satisfactory with good appearance, no bloated appearance, and the color and texture were similar to facial skin, and the activity of the neck were obviously improved

**Conclusions**

The expanded frontotemporal skin can provide relatively large area of thin flap, with reliable blood supply and good appearance after operation, and it is a better method to repair the scar contracture of the face and neck

### Abstract Topic: Scars Management and Pain Control

#### S100 Observation on the effect of medium frequency electrotherapy on burn scar

##### Yang Ruqian, Wang Xue, Yang Qin, Geng Kang, Yan Hong

###### Department of plastic and burn surgery, the Affiliated Hospital of Southwest Medical University, Luzhou, Sichuan, 646000, P.R.China

####### **Correspondence:** Yan Hong

**Background**

To observe the prevention and treatment effect of medium frequency electrotherapy on hypertrophic scar.

**Materials and Methods**

Subjects are selected form deep II and III degrees burn patients in our department between January 2016 and December 2018, and are randomly divided into control and observation groups .Subjects in the control group were treated with conventional antiscar therapy instead of medium frequency electrotherapy,meanwhile subjects in the observation group began to receive conventional antiscar therapy and medium frequency electrotherapy after the burn wound healing.The vancouver scar scale was used to assess the scar condition of patients in each group, and the softness of scar was measured with the shaw hardness scale. The scar volume was measured with 3D camera, and the thickness and gray scale of the scar were measured by skin ultrasound. the arrangement of scar collagen was observed through H&E staining. And itching and pain of scar were observed by Visual Analogue Scale (VAS). All the above tests were performed after wound healing, 3 months, 6 months and 9 months.

**Results**

The volume, thickness, softness,the score of VAS and itch and pain of hypertrophic scar in the observation group were significantly improved, which were lower than these in the control group. The results of paraffin section through light microscope showed that the structure of scar tissue in the observation group was rearranged and the collagen was changed.

**Conclusions**

Medium frequency electrotherapy can effectively inhibit scar hyperplasia and soften hypertrophic scar, promote the rearrangement of scar tissue structure, and reduce itching and pain caused by scar.

### Abstract Topic: Scars Management and Pain Control

#### S101 Numerical Simulation and Experimental Verification of Temperature Field on Frozen Skin Tissue Stabbed by Cryoprobe

##### Shiyuan Chen^1^, Xun Zhuge^1^, Li Wang^1^, Mengling Chang^2^, Zhinan Zhang^1^, Feng Guo^3^, Jingning Huan^2^

###### ^1^School of Mechanical Engineering, Shanghai Jiao Tong University, Shanghai, China; ^2^Department of Burn and Plastic Surgery, Rui Jin Hospital, School of Medicine, Shanghai Jiao Tong University, Shanghai, China; ^3^Department of Plastic Surgery, The Sixth People's Hospital, Shanghai Jiaotong University, Shanghai, China

####### **Correspondence:** Zhinan Zhang; Feng Guo

**Background**

Liquid nitrogen freezing treatment applies low temperature on the lesion to make necrosis and shedding for treatment, causing less bleeding and pain. Therefore, liquid nitrogen cryotherapy is widely used in cryosurgery. This study is to study the temperature field characteristics of frozen skin tissue stabbed by cryosurgery probe.

**Materials and Methods**

A three-dimensional model of skin is built based on the Pennes Bio-heat Equation. The model takes blood perfusion rate, metabolic heat generation values, the phase change of skin tissue etc. into account. The simulation of temperature field was performed with the finite element analysis software ANSYS, and the feasibility of the simulation method was verified by animal experiments.

**Results**

An effective cure scope and temperature distribution of the cryoprobe frozen by liquid nitrogen were gained was given. The maximum effective scope was 2.11mm from the surface of cryoprobe on skin surface, and it is 1.12mm underneath the cryoprobe inside the skin tissue, which corresponds well with verified experiment.

**Conclusions**

When using the cryoprobe in clinical application, the effective cure scope concluded in this paper should be considered to ensure a precision treatment.

### Abstract Topic: Scars Management and Pain Control

#### S102 Activated platelet-rich plasma therapy for second and third degree burns: a case series

##### Karina^1^, Ayu P.B. Sarena^2^, Tommy Toar^2^

###### ^1^Hayandra Clinic, Central Jakarta, Jakarta, Indonesia; ^2^Faculty of Medicine, Universitas Indonesia, Jakarta, Indonesia

####### **Correspondence:** Karina

**Background**

Burn injuries tend to be really painful and leaves a bad scar result, even after being treated by the current standard treatment.[1] Activated platelet-rich plasma (PRP) contains various growth factors in high concentration. PRP treatment in burns might accelerate wound healing, reduce pain, and leave a better scar result compared to the skin graft.[2]

**Materials and Methods**

This study reports 2 burn patients with second and third degree burns that were given platelet-rich plasma treatment. One patient had 18% TBSA with deep partial thickness and full thickness burns, while the other had deep partial thickness burns with 8% TBSA. The activated PRP was injected intravenously and intralesional to the patient after debridement procedure was done. The wounds was cleansed conventionally and no skin graft procedures were applied to all of the patients. We follow up these patients every week in 4 consecutive week after treatment.

**Results**

The patients reported they felt a great amount of pain reduction after receiving PRP treatment. All of the patients had 100% epithelization in 23 and 19 days post-treatment. After 1 months, no hypertrophic scar, pliability, or itchiness were reported.

**Conclusions**

Treatment by injection of intravenous and intralesional PRP, for second and third degree burn wounds, was found to be able to alleviate pain and give a better scar appearance. This therapy would be a great consideration to replace skin graft as the standard therapy.

### Abstract Topic: Surmounting Challenges in Rehabilitation

#### S103 Functional Transition after Axillary Burn Injuries

##### Yasutaka Shii¹, Yasuharu Matushita¹, Takaaki Aoki², Haruhiko Akiyama²

###### ¹Department of Rehabilitation Medicine, Gifu University School of Medicine, Gifu city, Gifu ken, Japan; ²Department of Orthopaedic Surgery, Gifu University, Gifu city, Gifu ken, Japan

####### **Correspondence:** Yasutaka Shii

**Background**

Axillary burns have a major effect on upper limb function. After the axillary burns, there are reports on life-saving rate, daily life level, upper limb function score. However, there are few reports that clearly describe the treatment outcome of upper limb function over time. The purpose of this study was to examine the recovery process of upper limb function after axillary burns.

**Materials and Methods**

The research period is from October 2013 to October 2015. We evaluated 7 patients (7 males) who underwent axillary burns and skin graft surgery at our hospital. Constant score and the Disabilities of the Arm, Shoulder and Hand scale (DASH) were assessed at 3 months, 6 months, 1 year and 2 years after injury. Statistical analysis was performed using Tukey test. The level of significance was set at p < 0.05.

**Results**

The average value of each of the Constant score was calculated and expressed in (3 months, 6 months, 1 year, and 2 years). Pain (10.3, 12, 14, 15), Activities of daily living (12.7, 18.6, 19, 20), Range of movement (28.2, 37.2, 38.8, 40), Power (5.7, 6.4, 10.2, 10). Constant score improved until one year after injury. The transition of DASH is also expressed in the same way. Disability (45, 43, 31.1, 20.4), Sports (75, 75, 31.2, 25), Work (100, 66.6, 43.7, 12.5). DASH improved until two years later.

**Conclusions**

Upper limb function after axillary burns was evaluated using Constant score and DASH. Constant score improved over time from 3 months to 1 year after injury. DASH improved until two years later.

### Abstract Topic: Surmounting Challenges in Rehabilitation

#### S104 Empowering caregivers: A positioning poster for burn patients and their family

##### Florence Chiang

###### Occupational Therapy Department, Singapore General Hospital, Singapore

**Background**

In 2017, a multi-disciplinary team from Singapore General Hospital commenced a long-term project (funded by SingHealth and Temasek Foundation International) to enhance the Dhaka (Bangladesh) healthcare team’s capacity in burns management. Prevention of contractures is one of the core tenets in burns rehabilitation, as burn contractures can lead to substantial deformities with devastating dysfunction. However, during the project period, it was observed that burn patients were not maintained in the optimal anti-contracture positioning. The objective of this project is to reduce the risk of contractures with the limited resources available.

**Materials and Methods**

A poster that illustrates various anti-contracture positioning was conceptualised. It used strong visuals and clear, succinct text in Bengali to convey the message, taking into consideration the wide variance in literacy level of the target audience. The posters were placed across burn wards in the Dhaka Medical College Hospital. Data was collected on the number of joints correctly positioned in nine patients before and after the posters were displayed. Seven caregivers and nine healthcare staff were interviewed to assess the level of their awareness of the poster and the usefulness of its content.

**Results**

Out of 34 joint positions observed, 3 were noted to have improved. Out of seven caregivers interviewed, four reported that they understood the poster content and attempted to follow but two verbalised difficulty following through as the patient was in pain. Among the nine healthcare staff interviewed, all agreed it was useful.

**Conclusions**

The poster has kick started initial changes to prevent contractures in burn patients. In the next phase of the project, we will focus on training the healthcare staff to use the posters to explain to caregivers. Strategies will also be taught to caregivers on how to position a patient with pain. Data will be collected to evaluate its impact.

### Abstract Topic: Surmounting Challenges in Rehabilitation

#### S105 Defining Burn Contractures: can we and should we?

##### RuthAnn Fanstone, Tom Potokar, Patricia Price

###### Centre for Global Burn Injury Policy and Research, Swansea University, Swansea, Wales, UK

####### **Correspondence:** RuthAnn Fanstone

**Background**

Literature suggests that burn contractures are preventable yet, at the same time, studies report a prevalence of 38% - 54%. Is this conflicting evidence as a result of a lack of consensus about the definition of a ‘contracture’ or how prevalence is calculated? Can we report with any certainty that contractures are, indeed, preventable if we haven’t yet fully understood risk factors associated with their development? This study aimed to investigate the current understanding of a burn contracture and how experts in the area use the term.

**Materials and Methods**

A comprehensive literature review and expert interviews (n=15) were conducted: experts were required to have more than 10 years clinical experience in the field.

**Results**

The majority of the literature is based on research and commentary from high-income countries. Whilst technical definitions of a burn contracture exist, there is limited evidence of a consensus or a standardized, operational definition of a contraction, how it should be measured, why a contracture should be measured or, indeed, when. Furthermore, classification of contracture severity varies. The experts stated that their working definitions of ‘contractures’ was based primarily on their own clinical experience, rather than the literature. Given the majority of literature in this area consists of clinical reports and opinion, this is understandable. Neither sources focused on detailed, operational definitions, which makes it difficult to build an evidence base for prevention or treatment based on standardized measurement protocols. Despite the lack of this operational definition, clinicians were confident of their ability to identify and measure a contraction.

**Conclusions**

Without a valid and reliable definition and measure of contracture, it is challenging to measure prevalence of burn contracture and explore risk and protective factors in burn contracture formation. Can and should the burn care community settle the question of what a contracture is and how to measure one?

### Abstract Topic: Tissue Banking

#### 106 A review of skin banking guidelines and standards worldwide: Towards the harmonisation of guidelines for skin banking in therapeutic applications for the regions under the Asia-Pacific Burns Association (APBA)

##### Wee Ling Heng ^1^, Alvin Wen Choong Chua^1,2^, and Si Jack Chong^3^

###### ^1^Transplant Tissue Centre, Singapore Health Services, Singapore; ^2^Department of Plastic, Reconstructive and Aesthetic Surgery, Singapore General Hospital, Singapore; ^3^Asia Pacific Burn Association, Bali, Indonesia

####### **Correspondence:** Alvin Wen Choong Chua

**Background**

Currently, there is no harmonised standard to govern skin banking in the Asia-Pacific region. Therefore, skin banks are either unregulated, or rely on their nations’ legislation or international accreditation to uphold their banks’ quality standards.

**Materials and Methods**

A new set of prescriptive skin banking guidelines was developed through a comprehensive review and collation of best practices for Asia-Pacific Burns Association members, from donor screening and testing, to skin recovery, processing, storage and distribution, and quality assurance. National regulatory requirements reviewed include the European Directives, India’s Transplantation of Human Organs Act, Therapeutic Goods Administration (TGA), Japanese Society of Tissue Transplantation and Singapore’s guidelines for tissue banking. These legislations are modified from international standards, which offer further technical and quality recommendations. These include the American Association of Tissue Banks (AATB), United States Food and Drug Administration Current Good Tissue Practice (CGTP), the European Good Tissue Practices (EuroGTP) Guide, and Asia-Pacific Association of Surgical Tissue Banking (APASTB).

**Results**

Adapted mainly from the AATB standards, these guidelines offer a comprehensive manual, addressing: governance and contracts; staff responsibilities; quality management; facilities, equipment and supplies management; donor consent and testing; recommendations of good practices pertaining to skin recovery, processing, storage and distribution. Besides complementing current generic regulations, they provide specifications of major aspects unaddressed in most legislations: (1)diseases and infections contraindicated for donation; (2)accreditation of testing laboratories; (3)archiving of donors’ serums; (4)corrective-and-preventive-actions (CAPA) investigations of not only post-operative adverse outcomes, but also errors and accidents; (5)recall procedure; (6)environmental monitoring of skin-processing facilities; (7)process validations prior to implementation of critical processes and revalidations.

**Conclusions**

A harmonised standard provides similar quality allografts among member states. It also eliminates the need to maintain exorbitant international accreditation and mandatory compliance to guidelines incompatible to our Asian context. This can also simplify the importation process when an exchange of allografts between transnational jurisdictions is required.

### Abstract Topic: Tissue Banking

#### S107 Knowledge and views of nurses on tissue donation

##### Qi Wei Bibi Wang^1^, Wen Ning Ho^1^, Yik Cheong Khoo^1^, Alvin Wen Choong Chua^2^

###### ^1^Transplant Tissue Centre, SingHealth, Singapore; ^2^Skin Bank Unit, Singapore General Hospital

####### **Correspondence:** Qi Wei Bibi Wang

**Background**

Of the Singapore population, only 3% pledged for donation, resulting in a shortage of tissues, such as heart valves, skin and vessels, available for transplantation.

As tissue donations are executed in hospitals and hospices, Transplant Coordinators work closely with nurses to facilitate the process. Nurses are often the first line of care for patients receiving treatment prior to their demise. Therefore, they can play an important role in donation by identifying potential donors or advocating for donation.

This study aims to gather a baseline regarding nurses’ attitudes and knowledge towards tissue donation, and how these deficits can be addressed to empower nurses to take a proactive role in the tissue donation process.

**Materials and Methods**

A survey was conducted from 2014 to 2017 with 1293 nurses working in two tertiary hospitals in Singapore before they attended an one-hour tissue donation awareness talk. Data collected included demographics and information on knowledge and attitudes towards tissue donation.

**Results**

Of the 1293 nurses surveyed, 59.7% of them were in favor of tissue donation although only 32.4% of the nurses had heard of the tissue donation legislation in Singapore. 874 nurses stated that they are uncomfortable approaching the deceased’s family for tissue donation; 76% of them felt that doing so would add distress to the family, p < 0.05, while only 5.6% (n = 49) felt that tissue donation is not part of their nursing responsibility, p < 0.05.

**Conclusions**

The analysis reflects that more than half of the nurses surveyed were supportive of tissue donation but education seems to be severely lacking. Therefore, healthcare professionals including nurses need to be enriched on donation education catered to their needs (e.g. changing their perception and family approach). This might empower them to play a proactive role in tissue donation in hopes of increasing the tissue donation rates in Singapore.

### Abstract Topic: Tissue Banking

#### S108 The Refinement of Decontamination Regimen of Skin Allografts in Singapore General Hospital

##### Zubaidah Binte Dadlani^1^, Si Xuan Tan ^2^, Tze Peng Lim ^2^, Jialin Yick ^1^, Wing Yue Chan^1^, Alvin Wen Choong Chua^1^

###### ^1^ Skin Bank Unit, Singapore General Hospital, Singapore; ^2^ Department of Pharmacy, Singapore General Hospital, Singapore

####### **Correspondence:** Zubaidah Binte Dadlani

**Background**

The Skin Bank Unit (SBU) in SGH decontaminates donated skin by incubation in Dulbecco’s Modified Eagle Medium (DMEM) containing Vancomycin and Amikacin (V+A). While effective against bacteria, this solution lacks an antifungal; thus, any fungal contaminated skin is unsuitable for transplant. In efforts to reduce tissue discard, SBU has tested Amphotericin B (AMB) – a cytotoxic but cost-effective antifungal with low Minimum Inhibitory Concentrations (MICs) and high susceptibility rates across a wide range of fungi – against keratinocytes and fibroblasts, finding that the highest tolerable concentration of the drug against skin is 1.0μg/ml. This study investigates the potency and fungicidal efficacy of 1.0μg/ml AMB to determine its suitability as an addition to SBU’s decontamination regimen.

**Materials and Methods**

DMEM containing AMB (1.0μg/ml), V+A (50μg/ml, 100μg/ml) was prepared and stored in dark conditions at 4°C. Solutions were aliquoted from this cocktail and frozen at various time-points. These solutions were tested by well-diffusion assay using *C. albicans* (C.A.), and their percentage potency was calculated by comparison to the 0-hour sample. In a separate test, the fungicidal activity of the cocktail was determined using a 96-hour in-vitro Time Kill Assay (TKA) model against C.A..

**Results**

AMB in the cocktail maintained an average of 46.8% potency at 48-hours (letting 0-hour concentration=100%); potencies at 72 and 96-hour time-points were below 37% (n=3). TKAs carried out against initial inocula of 3.48~3.86, 4.60~5.03 and 5.88~6.06 log Colony Forming Units/ml C.A. showed mean reductions of 99.0% (n=7), 99.7% (n=10) and 99.3% (n=4) respectively at 96 hours.

**Conclusions**

1.0μg/ml AMB can be incorporated into SBU’s decontamination regimen to potentially reduce the discard of tissues contaminated with C.A. and fungi with similar MICs. For optimal decontamination, skin should be incubated for 96 hours with media change at 48 hours. If implemented, microbiological trends in skin recovered must be monitored to validate AMB’s efficiency against clinical fungal strains.

### Abstract Topic: Tissue Engineering

#### S109 Studying the effects of Platelet Rich Plasma (PRP) and Adipose - Derived Mesenchymal Stem Cells (ADSCs) on chronic wound treatment

##### Tuan Ngoc Nguyen, Dzung Tien Nguyen1

###### Vietnam National Burns Hospital, Military Medical University, Hanoi, Vietnam

####### **Correspondence:** Tuan Ngoc Nguyen

**Background**

Chronic wound are breaks in the skin that do not heal, or require a long time to heal, and frequently recur. Autologous platelet-rich plasma (PRP) and Adipose – Derived Mesenchymal Stem Cells (ADSCs) are potential wound healing treatments in vitro and in vivo. In this study, we evaluated synergic effects of PRP and ADSCs on chronic wound treatment.

**Materials and Methods**

Thirty patients with chronic wounds treated at Wound Healing Center, National Institute of Burns from March 2016 to July 2017 were recruited for study. These patients were injected the combination of PRP and ADSCs at wound bed around. After one and two weeks of PRP and ADSCs injection, these patients were re-injected PRP. We estimated some clinical signals of wound bed and evaluated Periodic acid – Schiff (PAS) response before and after therapy

**Results**

PRP and ADSCs helped to improve the wound healing process: reduced exudate, promoted the epithelialization, granulation tissue; increased sample number of PAS response at epiderma and basement membrane, reduced MMP-12 after therapy

**Conclusions**

The combination of PRP and ADSCs stimulated wound healing process by improvement of wound edge and wound bed

### Abstract Topic: Tissue Engineering

#### S110 Analysis on efficacy of rhGM - CSF in deep partial thickness burn wound healing and its underlying mechanism

##### Yuhui Dongye^1,2^, Dongyu Li^3^, Kaipan Qu^4^, Chenghao Hu^1,2^, Xing Yang ^1,2^, Haibo Zhang^2,5^, Jiani Wu^2^, Weifeng Li^2^, Huibin Li^2^

###### ^1^School of Clinical Medicine, Weifang Medical University, Weifang, Shandong 261000; ^2^Department of Burns and Plastic Surgery, People's Hospital of Linyi, Linyi, Shandong 276000, P.R. China; ^3^Department of Genetics, College of Agricultural and Life Science, University of Wisconsin‑Madison, Madison, WI 53706, USA; ^4^Department of Burns and Plastic Surgery, Shandong Provincial Third Hospital, Shandong, Jinan,250000; ^5^School of Clinical Medicine, Binzhou Medical University, Yantai, Shandong, 256600, China.

####### **Correspondence:** Huibin Li

**Background**

Management of deep II°burn is a challenge for physicians. rhGM-CSF has been proved to be effective in wound healing. However, the underlying mechanisms haven’t been fully comprehended. It is meaningful to investigate the mechanisms that promote wound healing.

**Materials and Methods**

From 2015 to 2016, 96 cases of deep II° burn wound wounds in Linyi People’s hospital were randomly divided into experimental group and controlled group. The experimental group was treated by rhGM-CSF hydrogel, the control group received traditional treatment of Vaseline gauze bandage. The whole experimental period was 28 days. Wound healing time and wound healing rate, total effective rate at the 7th,14th and 21th day after treatment were observed and recorded. The tissue samples(n=6) harvested from burn wound at different time point were obtained for detecting the expression of TGF-β1 and TβRII through immunohistochemical method.

**Results**

The average wound healing time in the experimental groups was significantly shorter than that in the controlled group(p<0.05).The wound healing rate and efficacy in the experimental groups was significantly higher than that in the controlled group (p<0.05). The total effective rates in the experimental groups respectively at 7^th^,14^th^ and 21^st^ after remedy was also significantly higher than the control group (p<0.05). The expression of TGF-β1 and TβRII in the experiment group at 7^th^, 14^th^, 21^st^ was significantly much more than that in control group, respectively(p<0.05).

**Conclusions**

Topical application of rhGM-CSF hydrogel can significantly promote the expression of TGF-β1 and TβRII in wound tissue, accelerating and enhancing the healing process of deep II°burn wound.

### Abstract Topic: Tissue Engineering

#### S111 An in vitro and in vivo study of antimicrobial effect of a gallium-loaded artificial dermal scaffold

##### Zhaorong Xu^1^, Zhaofan Xia^2^

###### ^1^Fujian Burn Institute, Fujian Medical University Union Hospital, Fuzhou, Fujian, China; ^2^Department of Burn Surgery, Changhai Hospital, Second Military Medical University, Shanghai, China

####### **Correspondence:** Zhaofan Xia

**Background**

Currently, artificial dermal scaffolds are widely used for the treatment of full-thickness skin defects, but the problem that biomaterial-associated infections frequently occur with their use, as it greatly affects the survival of the scaffolds, must be addressed. The gallium (Ga), which has broad-spectrum antimicrobial effect, is a promising candidate to prevent implant-associated infections.

**Materials and Methods**

The gallium-loaded antimicrobial artificial dermal scaffold was prepared by gallium ions and a collagen solution, followed by material characterization. The antibacterial rate of the scaffold was evaluated by a direct contact assay using standardized cultures of *S. aureus* and *P. aeruginosa*, and cytological evaluation was conducted using L929 cells. Then the full-thickness wounds on both sides of the back of SD rats, which were added with a certain amount of *P. aeruginosa* suspension, were used for the evaluation of antimicrobial activity on wounds *in vivo*.

**Results**

The characterization results showed a porous structure and a appropriate enzymatic degradation rate, as well as a sustaining releasing of gallium ions. *In vitro* antimicrobial testing revealed that the 1 h antibacterial rate against *S. aureus* and *P. aeruginosa* was close to 90%, which indicated its great antimicrobial activity. The results of the cytological evaluation showed no cytotoxicity. Furthermore, the *in vivo* antimicrobial evaluation showed the successful prevention of wound infections in SD rats, and the HE staining also demonstrated its great biocompatibility.

**Conclusions**

This gallium-loaded antimicrobial artificial dermal scaffold exerted excellent antimicrobial activity and no cytotoxicity *in vitro*. Furthermore, it could effectively prevent the infection of wounds and implants, which warrants further research for future clinical applications.

### Abstract Topic: Tissue Engineering

#### S112 The application of dermal regeneration templates in full-thickness skin defects reconstruction

##### Zhaohong Chen, Zhaorong Xu, Shun Chen, Haihui Li, Wei Liu, Jianchang Lin, Zi’en Wang, Shaohua Wang, Changdan Jin, Yichao Xu, Jianjun Zheng, Linwen Zheng

###### Fujian Burn Institute, Fujian Medical University Union Hospital, Fuzhou, Fujian, China

####### **Correspondence:** Zhaohong Chen

**Background**

Dermal regeneration templates (DRTs), such as Integra® introduced as a new alternative to our surgical arsenal and applied in various types of wound cases, have gained great importance. They have been widely used in reconstructive surgery around the world. Here, we decribe the approving results of full-thickness skin defects reconstruction with DRTs.

**Materials and Methods**

The data of patients with full-thickness skin defects who were treated with DRTs, was collected from Fujian Burn Institute in China. Wound bed preparation (debridement or negtive pressure wound therapy) were received previous to DRTs, and second-stage split thickness skin graft (STSG) were performed two weeks after DRTs transplatation. Wound healing rate, clinical and photographic evaluations, and complications related to their use were analyzed.

**Results**

Patients with different cases were treated with DRTs, some with acute burn wound, while others with chronic wound, soft tissue defect with exposed tendon or wound due to scar excison. No significant infection was observed after surgery, and the donor site morbidity was minimal. All patients healed with STSG take rate over 90%, and flexible skin coverage were obtained. Most cases had achieved smooth surface and satisfied articular movement, as they were all followed up for three months to more than one year.

**Conclusions**

DRTs offer a convenient and efficient operative procedure with minimal morbidity, demonstrating excellent aesthetic and functional healing effect. Thus, DRTs may offer an alternative option with low risk for full-thickness skin defects reconstruction.

### Abstract Topic: Tissue Engineering

#### S113 The growth of diced cartilage and the role of fibrin glue scaffold and platelet rich plasma in subcutaneous implantation and tissue engineering using vascularized perforated tube

##### Nyoman P Riasa^1^, I Putu Astawa^2^, I Nyoman Mantik-Astawa^3^, I Nyoman Agus-Bagiada^4^

###### ^1^Department of Plastic Reconstructive and Aesthetic Surgery, Faculty of Medicine Udayana University-Sanglah Hospital, Denpasar Bali, Indonesia; ^2^Department of Orthopaedic and Traumatology, Faculty of Medicine Udayana University-Sanglah Hospital, Denpasar Bali, Indonesia; ^3^Department of Pathology, Faculty of Veterinary Udayana University, Denpasar Bali, Indonesia; ^4^Department of Biochemistry, Faculty of Medicine Udayana University, Denpasar Bali, Indonesia

####### **Correspondence:** Nyoman P Riasa^1^

**Background**

In plastic surgery, cartilage could be used as diced cartilage (DC) graft. Fibrin glue scaffold (FGS) and platelet rich plasma (PRP) had been added in grafting process but its role need to be elucidated. This study aimed to explore DC growth and the role of FGS and PRP in subcutaneous DC implantation and to use DC as cell source to developed larger construct using vascularized perforated tube (VPT).

**Materials and Methods**

In each of eight rabbits, two dorsal subcutaneous pockets and two VPT on superficial epigastric vessels were created. VPT was made of perforated silicone tube containing superficial epigastric pedicle. Mixture of FGS_PRP were made by retrieving PRP from centrifugation of 2 ml autologous blood in citrate natrium and activated with calcium chloride and thrombin during mixing process with DC. DC as well as DC + FGS_PRP were implanted on VPT and subcutaneous pocket. Subcutaneous pockets DC implantation were use as control group.

**Results**

Eight weeks after implantation all specimens shows glossy cartilage. VPT groups show larger construct with higher tissue weight (p < 0.05) and vascularized tissue flap containing cartilage could be created. DC with new chondrocytes island formations was noted, and connective tissues containing blood vessels fill the space in between. ANOVA test show no difference for viable DC and positive GFAP staining in all groups (p > 0.05), but chondrocytes viability were higher compare to the control group (p < 0.05). Formation of new chondrocytes islands was higher in subcutaneous DC + PRP_FGS group and in VPT DC only group but with lower vascularity compare to the control group (p < 0.05).

**Conclusions**

It can be concluded that PRP_FGS has positive effect on subcutaneous DC implantation and to use DC only in creating larger construct of vascularized tissue flap containing cartilage.

### Abstract Topic: Tissue Engineering

#### S114 Construction and in vitro evaluation of VEGF gene activated PLGA knitted mesh-reinforced collagen/chitosan dermal scaffold

##### Tingting Weng, Pan Wu, Wei Zhang, Xingang Wang

###### Department of Burns & Wound Care Center, Second Affiliated Hospital of Medical College, Zhejiang University, Hangzhou 310009, China

####### **Correspondence:** Wei Zhang

**Background**

Dermal substitutes play an important role in dermis reconstruction /regeneration. However, there is still the problem of insufficient vascularization. The process of angiogenesis is precisely regulated by various cytokines/growth factors. The introduction of pro-angiogenic factors into dermal scaffolds in gene form to positively regulate the vascularization process is a hot spot in vascularization research. VEGF is one of the main regulators of neovascularization. VEGF can specifically combine with VEGFR-2 receptor on the surface of endothelial cells to promote the migration, proliferation and primary lumen formation of endothelial cells.

**Materials and Methods**

A cationic gene delivery vector, N,N,N-trimethyl chitosan chloride (TMC), was used to form complexes with the plasmid DNA encoding VEGF. Then, the TMC/pDNA–VEGF was incorporated into the dermal scaffold to construct VEGF gene activated dermal scaffold. Next, the scaffold was physically characterized. Subsequently, TMC/pDNA–EGFP, naked pDNA–VEGF and blank PLGAm/CCS were loaded respectively as controls. The effect of VEGF gene-activated PLGAm/CCS dermal scaffold on cell proliferation was investigated by vaccinating HUVEC in vitro.

**Results**

We have successfully prepared VEGF gene-activated PLGAm/CCS dermal scaffold. The scaffolds have a good pore structure and a certain sustained release capacity. The sustained release of TMC/pDNA–VEGF has an ideal cell transfection efficiency. HUVECs transfected with TMC/pDNA-VEGF can maintain good proliferative activity and secrete VEGF protein.

**Conclusions**

The results show that the VEGF gene activated PLGAm/CCS dermal scaffold is expected to become a new functional tissue engineering skin for promoting vascularization. Its vascularization properties in vivo can be further confirmed through in vivo research.

### Abstract Topic: Tissue Engineering

#### S115 Collagen-based dermal substitute with stable fibroblast growth factor 2 (FGF2): *ex ovo* and *in vivo* impact on neovascularization

##### Bretislav Lipovy^1,2^, Lucy Vojtova^3^, Jakub Holoubek^1,2^, Jan Zidek^3^, Martin Knoz^1,2,4^, Johana Babrnakova^3^, Veronika Pavlinakova^3^, Lucie Vistejnova^5^, Veronika Stepankova ^6^, Eva Filova ^7^, Zdenek Pavlovsky^8^, Martin Faldyna^9^, Eduard Göpfert^9^, Jiri Damborsky^10^, Vanessa Hearnden^11^

###### ^1^Department of Burns and Plastic Surgery, University Hospital Brno, Czech Republic; ^2^Faculty of Medicine, Masaryk University Brno, Czech Republic; ^3^CEITEC – Central European Institute of Technology, Brno University of Technology, Brno, Czech Republic; ^4^Department of Plastic and Aesthetic Surgery, St. Ann‘s University Hospital, Brno, Czech Republic; ^5^Biomedical Center, Faculty of Medicine in Pilsen, Charles University, Czech Republic; ^6^Enantis s.r.o, Brno, Czech Republic; ^7^Institute of Experimental Medicine, Academy of Science of the Czech Republic, Prague; ^8^Department of Pathology, University Hospital Brno, Czech Republic; ^9^Veterinary Research Institute, Brno, Czech Republic; ^10^Institute of Experimental Biology and Recetox, Masaryk University, Brno, Czech Republic; ^11^Department of Materials Science and Engineering, Department, University of Sheffield, UK

####### **Correspondence:** Bretislav Lipovy

**Background**

Dermal substitutes play important roles in the treatment of deep dermal and full thickness wound in both phases (acute and long-term). Neovascularization is very important within whole wound healing procedure (improve metabolic and oxygen ratio in the tissue, facilitate of transmigration other growth factors, etc.)

**Materials and Methods**

Unique bi-layer dermal substitute consists of porous biopolymer foam coated with polymeric nanofibers and highly stable fibroblast growth factor (FGF2). For measurement of impact on neovascularization two different methods were used. First *ex ovo* methodology was provided in terms of Chick Chorioallantoic Membrane (CAM) assay, second method was provided via mathematical establishingcapillary density in histological samples stained with α-SMA (α-smooth muscle actin) and anti-FVIII (neovascularization) markers from large white pigs.

**Results**

Firstly, in both experiments, we have proven that our tested material has demonstrated full biological compatibility. As confirmed by CAM assay and animal model testing, stable FGF2 enhanced bi-layer neovascularization efficiency. Positive influence of FGF2 was proved as well by neovascularization and fibroproliferation with autologous collagen production in the entire animal model defect.

**Conclusions**

Stable FGF2 demonstrated positive biological activity in relation to neovascularization in both the CAM assay and the animal model.

**Acknowledgement**

This research was supported by the Ministry of Education, Youth and Sports of the Czech Republic under the projects CEITEC 2020 (LQ1601), LO1503 and LO1218 as well as by Ministry of Health of the Czech Republic, grant Nr. 17-29874A. All rights reserve

### Abstract Topic: Tissue Engineering

#### S116 The properties of collagen-chitosan nanosilver antibacterial dressing and its effect on the healing of full-thickness burn wounds

##### Xiaodong Chen, Rongfu Li, Zhaorong Xu, Qiong Jiang, Zhaohong Chen

###### Fujian Burn Institute, Fujian Medical University Union Hospital, Fuzhou, Fujian, China

####### **Correspondence:** Xiaodong Chen

**Background**

For dressings, how to prevent wound infection and promote wound healing is a key issue to be solved. Chitosan and collagen have been widely studied and applied as biological dressings, while nano-silver has a good broad-spectrum antibacterial effect, and has fixation, stability and antibacterial properties on the surface of the dressing.

**Materials and Methods**

In this study, HUVECs were cultured in vitro to evaluate the effects of the main components of collagen chitosan nano-silver dressing on the proliferation of cells and the secretion of growth factors and inflammatory factors. A dressing was prepared and tested for its properties and antibacterial properties. By further establishing a full-thickness skin burn model in rats, the effects of dressing on wound healing in rats and the effects of tissue growth factors and inflammatory factors on wounds were observed.

**Results**

The composition of the dressings with different molecular weight collagen and chitosan solution and their mixed solution can promote the proliferation of HUVECs, and promote the secretion of cell growth factors in vitro. The chitosan solution and collagen chitosan mixture can inhibit the secretion of inflammatory cytokines from HUVECs. When the concentration of nano-silver was 2.5 mg/L or less, it did not affect the proliferation of HUVECs and the secretion of cell growth factors and inflammatory factors. Nano-silver can be stably combined with collagen chitosan which releases lower, and it has good water absorption. It has certain antibacterial activity against Pseudomonas aeruginosa, Escherichia coli and Staphylococcus aureus, and the inhibition rate is over 99%. Dressing can significantly promote growth factors, and regulate inflammatory factors in wound tissue in rat and can significantly promote wound healing.

**Conclusions**

Collagen chitosan nano-silver dressing has certain antibacterial properties, can promote wound healing, and regulate wound tissue growth factor production and inflammatory response.

### Abstract Topic: Nursing Care in Burn Centre

#### S117 Clinical Experience of Calcium Alginate Dressing in Burn Wounds

##### Hsueh-Yin Chang

###### Burn Center, Tri-Service General, Taipei, Taiwan, R.O.C

**Background**

Calcium alginate dressings are a strong, versatile, and natural wound care dressing. The application of calcium alginate dressing on wounds has been widely recognized. It maintain a physiologically moist microenvironment that promotes wound healing and the formation of granulation tissue. Calcium alginate is readily removed with saline irrigation, making dressing changes virtually painless.

**Materials and Methods**

Use normal saline solution to clean the wound area, then pat the area around the wound dry. Place the alginate dressing over the wound, and Secure a secondary dressing over the alginate dressing to hold it in place. Change the dressing every one to three days, according to the progress of the wound change the dressing or when fluid starts to seep out from the edges of the dressings. Before removing the alginate during a dressing change, use saline to dampen it in order to lower the risk of damaging the surrounding skin.

**Results**

Calcium alginate has a staggering effect and can promote tissue proliferation. It is an effective haemostat, it well tolerated by body tissues. Good epidermal healing was seen on all wounds although cellular reactions could be provoked in full thickness wounds without occlusion.

**Conclusions**

Calcium alginate can be used on acute burn wounds, traumatic wounds, partial- or full-thickness wounds, any wound with some drainage or exudate. Calcium alginate turns into a gel when exposed to the exudate to provide an ideal healing environment, making the removal of the dressing virtually painless. Calcium alginate is an important part of the wound care dressings.

### Abstract Topic: Wound Care Chronic Dressing

#### S118 Evaluation of biological activity of new temporary biopolymer hydrogel for selected growth factors in partial skin defects on the animal model

##### Bartošková^1^, Holoubek J.^1,2^, Lipový B.^1,2^ , Nedomova E.^3^, Ruzicka F.^4^, Vacek L.^4^ , Knoz M.^1,2,5^, Stepankova V.^6^, Pavlovsky Z.^7^, Faldyna M.^8^, Göpfert E.^8^, Damborsky J.^9^, Vojtova L.^3^

###### ^1^Department of Burns and Plastic Surgery, University Hospital Brno, Czech Republic; ^2^Faculty of Medicine, Masaryk University Brno, Czech Republic; ^3^CEITEC – Central European Institute of Technology, Brno University of Technology, Brno, Czech Republic; ^4^Department of Clinical Microbiology, St. Ann’s University Hospital, Brno, Czech Republic; ^5^Department of Plastic and Aesthetic Surgery, St. Ann‘s University Hospital, Brno, Czech Republic; ^6^Enantis, Brno, Czech Republic; ^7^Department of Pathology, University Hospital Brno, Czech Republic; ^8^Veterinary Research Institute, Brno, Czech Republic; ^9^Institute of Experimental Biology and RECETOX, Masaryk University, Brno, Czech Republic

####### **Correspondence:** Bartošková; Holoubek J.

**Background**

Nowadays, the demand for utilization of new technologies in clinical medicine is on rise. The same situation is in field of wound healing and there is a great opportunity for applying advanced biopolymer materials in clinical practice.. The aim of the treatment of acute and chronic defects caused by high energy, such as burn trauma or mechanical trauma, is not only survival but also the achievement of the best quality of life.

**Materials and Methods**

We present a unique antibacterial hydrogel for wound healing based on natural Gum karaya polysaccharide (GK) containing upgraded stable forms of growth factor for fibroblasts (FGF2). In preliminary in vitro testing, we verified the viability of colonized mouse monoclonal B lymphocytes (containing receptor for FGF2) on the developed material. The antimicrobial potential of the carrier was then verified using a disk diffusion assay followed by a measurement of the growth curves at OD 600 nm. Based on laboratory tests, in vivo experiments on the animal model were then performed.

**Results**

The results of the in vitro tests have demonstrated the biological activity of both mouse B lymphocytes and fibroblasts under the synergic effect of all key components on the tested material. The results of the antimicrobial tests showed a very promising antibacterial activity against wide spectrum of pathogen, especially against G+ spectrum.

In vivo test on animals brought a positive and successful result; all subjects survive and fully healed.

**Conclusions**

In vitro on eukaryotic cells and prokaryotic bacteria have shown a very promising result of GK based hydrogels. The histological and clinical outcome obtained showed a great potentials and support further development and testing.

### Abstract Topic: Wound Care Chronic Dressing

#### S119 Characteristics of Diabetic foot ulcer in Northern Vietnam: Analysis of Database from Wound Healing Center, Vietnam National Burn Hospital from 2013 to 2018

##### Dzung Tien Nguyen, Han Van Dinh, Dzung Thi Bui

###### Wound Healing Center, Vietnam National Burns Hospital, Military Medical University, Hanoi, Vietnam

####### **Correspondence:** Dzung Tien Nguyen

**Background**

Incidence of *Diabetic foot ulcer* has been arising unexpectedly in recent years in Vietnam. We will review data of patients with *Diabetic foot ulcer in Northern Vietnam in this retrospective cohort study.*

**Materials and Methods**

In this retrospective cohort study at Wound Healing Center, National Institute of Burns in Hanoi, Vietnam, we analyzed the electrical data of 469 patients with *Diabetic foot ulcer*, who hospitalized at Wound Healing Center from 2013 to 2018.

**Results**

Among 469 patients recruited for this study,76.75% were males and 23.24% were females. 68% of patients came from rural areas. Diabetic foot ulcers were most prevalent in age group from 40 to 60 years old (52.7%) and least prevalent in the age group from 20-30 years old (3.2%). Most patients had type 2 diabetes mellitus (92.3%) and required multidrug therapy or insulin (84.4%). 84% of patients didn't know how to recognize a problem with their feet before it becomes a major issue. 65% of them didn't find out they had diabetes until the ulcer appeared. Neuropathy was present in 70 % patients. Absent or diminished peripheral pulses was observed in 83% cases. Highest percentages of cases (54.58%) had grade 3 diabetic foot (according to Wagner's classification). 51.6% of cases had Osteomyelitis. 96% of patients had been closed wound by surgical intervention (skin graft (55.11%), flap (23.11%) or amputation (21.78%)).

**Conclusions**

In Northern Vietnam, all most patients with Diabetic foot ulcer haven't any understands about Diabetic foot ulcer risks and prevention methods. Many of them has peripheral neuropathy, peripheral arterial disease, osteomyelitis and require surgical intervention.

### Abstract Topic: Wound Care Chronic Dressing

#### S120 To observe the clinical effect of platelet rich fibrin in chronic sinus wound

##### Ding Xiaobin, Liu Ligang, Jiang Bo, Tang Rui1, Wu Xunlian, Zheng Danyu, Yan Hong

###### Department of Plastic and Burn Surgery, the Affiliated Hospital of Southwest Medical University, Luzhou, Sichuan, 646000, P.R.China

####### **Correspondence:** Ding Xiaobin

**Background**

There is an increasing number of bone exposure caused by chronic wound, as well as the proportion of patients calling for amputation or demanding skin flap graft. Amputation surgery is difficult to carry on. Meanwhile, the financial burden and low quality of life after amputation cannot be afforded for many patients. Due to the reasons above, we investigate the feasibility and prospect of using CO_2_ laser debridement combined with complex PRF wound cover after bone ablation

**Materials and Methods**

We use CO_2_ laser to produce multiple ablation points of different apertures and even lead to bleeding. To debride the surrounding tissue around bone exposure with CO_2_ laser, and use PRF to cover the exposed part timely in the meantime, to observe the recovery outcomes of wounds

**Results**

Following this treatment, the ablated bone tissue grew out major granulations and formed new granulation combined with PRF in a large number of patients. Since the treatment could be done outpatient, it could not only avoid the risk of amputation caused by osteonecrosis or skin flap transplantation, but also save medical resources as well as easing patients’ burden

**Conclusions**

This treatment could protect bone tissue and repair wound fairly well, which is worthy of being widely applied in clinic

### Abstract Topic: Wound Care Chronic Dressing

#### S121 Effects of Electrical Stimulation on Wound Healing and Angiogenesis in Diabetic Rats

##### Geng Kang, Wang Xue, Yang Yuting, Liu Lan, Yan Hong

###### Department of Plastic and Burn Surgery, the Affiliated Hospital of Southwest Medical University, Luzhou, Sichuan, 646000, P.R.China

####### **Correspondence:** Geng Kang

**Background**

To investigate the effects of electrical stimulation therapy on wound healing and angiogenesis in diabetic rats and its potential mechanism

**Materials and Methods**

Diabetic model rats were induced by a single tail vein injection of STZ (50 mg/kg) and a full-thickness skin defect of 1 cm2 was formed on the back. The model rats were randomly divided into two groups: the model group and the electrical stimulation treatment group, and the normal rats were used to cause the back wounds as a blank control group. The treatment group was treated with electrical stimulation (Bidirectional asymmetric pulse current, frequency: 100Hz, rise time: 3s, fall time: 3s, pulse width: 150us, duration: 3s, rest time: 9s)for 21 days. The control group and the model group were fed normally. The wound healing was evaluated on the 3rd, 7th, 14th and 21st day after treatment. The pathological morphology of rat wounds was observed by HE staining. The contents of serum eNOS, Ang-1 and VEGF were detected by enzyme-linked immunosorbent assay. The expression levels of Tie-2 and VEGFR2 protein were detected by immunohistochemistry and Western-blotting

**Results**

After electrical stimulation, the wound healing rate of diabetic rats was close to 90% on the 14th day, while the model group was less than 60%. The serum eNOS, Ang-1 and VEGF levels were significantly higher than the model group (*P*<0.05 or *P*<0.01). Meanwhile, the treatment group had more neovascularization, larger vascular lumen, high expression of Tie-2 and VEGFR2 around the blood vessels, and the expression of Tie-2 and VEGFR2 protein in the wound tissue was also significantly increased, which was statistically different from model group (*P*<0.05 or *P*<0.01) .

**Conclusions**

Electrostimulation therapy can significantly promote wound healing and neovascularization in diabetic rats, its mechanism is related to the increase of eNOS, VEGF, Ang-1 content and up-regulation of VEGFR2 and Tie-2 levels

### Abstract Topic: Wound Care Chronic Dressing

#### S122 Application of Ulcer Dressing in Superficial Second Degree Burn Wounds

##### Hua-Xi Niu, Li-Ying Wang, GuanLong Yang

###### First People’s hospital of Zhengzhou, Zhengzhou, Henan, China

####### **Correspondence:** Hua-Xi Niu

**Background**

Observe the clinical efficacy of Ulcer Dressing in superficial second degree burns

**Materials and Methods**

From January 2016 to December 2018, 102 patients were treated in our department, including 76 males and 26 females; aged 3-56 years, with an average of 21.3 years. The burn area was between 0.5% and 13%, with an average of 4.6%. The hospitalized patients were evaluated to determine that the burn was a shallow second degree wound. After cleaning and disinfection, the skin was cut off and the Ulcer Dressing was protected. The yarn is fixed and replaced according to the exudation condition. It is usually replaced within 3 days to 5 days

**Results**

The wounds in 102 cases were all within 7-10 days. The number of dressings was 2-3 times, and the pain was relieved obviously

**Conclusions**

Ulcer Dressings can be used as skin protection burn wounds, shorten wound healing time, reduce the number of dressings, reduce pain, and have satisfactory treatment results.

### Abstract Topic: Wound Care Chronic Dressing

#### S123 Case Report : Treatment of 3^rd^ degree burn of the hand using Kaloderm® (cultured allogenic keratinocyte)

##### Jangyoun Choi, Jin Tae Cho, Sung-No Jung

###### Department of Plastic and Reconstructive Surgery, Uijeongbu St. Mary’s Hospital, College of Medicine, The Catholic University of Korea, Uijeongbu, Republic of Korea

####### **Correspondence:** Sung-No Jung

**Background**

Allogenic keratinocyte application is known as an effective method for treating 1^st^ to 2^nd^ degree burns. However, there is only little literature reporting the application of allogenic keratinocyte on 3^rd^ degree burns. For some 3^rd^ degree burns where definitive surgical reconstruction is precluded due to medical comorbidity of the patient, we investigated the possibility of allogenic keratinocyte as a treatment modality to deep burns that might allow us to avoid high-risk anesthesia and surgery.

**Materials and Methods**

A 85 year old female patient visited our burn center due to a contact burn on her left hand. A 10 x 15 cm sized deep 2^nd^ to 3^rd^ degree burn wound was noted on the dorsoradial aspect of the left-hand dorsum. Considering the surface area and wound depth, surgery was indicated but her medical condition and age exhibited a very high risk for a lengthy operation. Therefore, a chemical escharectomy, serial bedside debridement, and allogenic keratinocyte (Kaloderm®, Tegoscience, Seoul, Korea) was applied.

**Results**

Chemical escharolysis was undertaken with silver sulfadiazine. Mechanical eschar debridement was performed serially as well. After complete wound bed preparation, Kaloderm® was applied to the wound bed. Kaloderm was changed every three days, considering the engraftment period of keratinocytes to the wound bed. The wound was completely epithelialized after four rounds of Kaloderm® application.

**Conclusions**

In most cases, treatment of burn wound using allogenic keratinocyte is limited to deep 2^nd^ degree burns. Surgical intervention is widely accepted as the mainstay of treatment for 3^rd^ degree burns. However, for circumstances where sharp debridement and definitive reconstruction is impossible due to patient’s medical condition, serial debridement and escharolysis followed by allogenic keratinocyte therapy can be a safe and reliable option for 3^rd^ degree burns.

Informed consent to publish has been obtained from this patient.

### Abstract Topic: Others

#### S124 Prevention Oriented Burns Epidemiology: The First Hospital Based Study from Bangladesh

##### Tanveer Ahmed^1^, Md. Abul Kalam^2^, Md., Rayhana Awwal^3^, Hossain Imam^4^

###### ^1^Assistant Professor of Plastic Surgery, Dhaka Medical College and Hospital, Dhaka, Bangladesh; ^2^Professor of Plastic Surgery, Dhaka Medical College and Hospital, Dhaka, Bangladesh; ^3^Professor and Head of Plastic Surgery, Dhaka Medical College and Hospital, Dhaka, Bangladesh; ^4^Assistant Professor of Plastic Surgery, Dhaka Medical College and Hospital, Dhaka, Bangladesh

####### **Correspondence:** Tanveer Ahmed

**Background**

Bangladesh is a developing country with a 180 million people where the burn incidence is increasing very sharp due to rapid urbanization. The aim of the study was to determine the epidemiology of burns leading to hospitalization in a resource-poor country like Bangladesh with a focus on the pre-event phase of injury.

**Materials and Methods**

In 2017, total 6913 victims were hospitalized from 52100 who appeared in the emergency and OPD of the only Burn & Plastic Surgery Institute and all of them were enrolled in a descriptive study. A simple data sheet was created to supply with every admitted patient focusing on to obtain burn injury related information necessary for prevention purposes.

**Results**

Males constituted 56% of victims, mean age was 22 years. The most severe burns occurred between the ages of 18 and 32 years mainly flame related (47%). However, scalds representing 45% of burns cases. Irrespective of types, women suffered more severe burns & mortality than men and the severe cases in children were under the age of 5 years. More than 80% of burns occurred at home where the kitchen was the main place of injury in 47% of cases, followed by living rooms in 28%. The main container was the rice cooking pot in 37%, followed by water kettles in 32% cases. The difference between a flame and non-flame burns in the distribution of burns in extremities was not statistically significant, but head and neck burns were 3.7 times more likely to be caused by flame.

**Conclusions**

So, the devastation of burn can be reduced by adopting a good prevention program.

### Abstract Topic: Others

#### S125 A first regional study on the efficacy of a protocol : radiotherapy treatment after excision of keloid scars

##### Teh Hui Yin^1^, Janna Joethy^2^, Chong Si Jack^3^

###### ^1^Ministry of Health Holdings, Singapore; ^2^Department of Plastic, Reconstructive and Aesthetics Surgery, Singapore General Hospital, Singapore; ^3^Asia Pacific Burn Association, Bali, Indonesia

####### **Correspondence:** Teh Hui Yin

**Background**

The combination of surgery and post-operative irradiation is the best treatment for keloid scars. Ogawa describes that the post-operative radiation response rate in terms of significantly reducing the rate of keloid recurrence ranges between 67-98%.

**Materials and Methods**

We present the first regional ( south east Asia) cohort study on in terms of improvement in scar appearance following a protocol in a national centre where radiation therapy treatment is delivered after keloidectomy

**Results**

11 consecutive patients in a single centre had excision of keloid scars followed by radiotherapy as part of a new protocol in our institution at various body sites were recruited in this IRB approved study. 7 out of 11 patients received radiation therapy of varying doses and fractions post-operatively while the rest opted out after surgery.

The post-operative irradiated scars were given scores based on the Vancouver Scar Scale by a single investigator at least 1 year after completion of radiation therapy. The Vancouver Scar Scale score ranges from 0 to 13 of which a score of 0 represents normal skin. Patients whom received post-operative radiotherapy had a score of </= 4 with a mean score of 2 as compared to patients who only had surgical excision with a score of >/= 5 with a mean score of 8.

The highest score of 12 belonged to a patient who had only surgery and the lowest score of 0 was rated in a patient who received post-operative radiation therapy. All patients who received radiotherapy post-keloidectomy have lower VSS score as compared to those who only have had surgical excision.

**Conclusion**

This first regional study concludes that all patients with keloid scars who received post-operative radiotherapy treatment resulted in a better scar appearance which more closely resembles normal skin as compared to surgical excision only.

### Abstract Topic: Others

#### S126 Classification of Electrical Burns Injuries of the Upper Limb: The Dhaka Experience

##### Tanveer Ahmed^1^, Barbara Jemec^2^, Rob Staruch^3^, Mahbub Hasan^1^

###### ^1^Plastic Surgery, Dhaka Medical College and Hospital, Dhaka, Bangladesh; ^2^The Royal Free Hospital (London), UK**;**^3^Defense Deanery, Burns and Plastics, DPhil Candidate University of Oxford

####### **Correspondence:** Tanveer Ahmed

**Background**

Electrical burns of the upper limb are devastating injuries. To date there is no specific classifications of electrical burns in the upper limb.

Due to the high number of electrical burn injuries treated in our institute we offer a classification and treatment algorithm for the immediate reanimation with tendon transfers after soft tissue cover in electrical burns of the upper limb.

**Materials and Methods**

Patient data was collected over one year and analysed with regards to sex distribution, high and low voltage burns, the areas of the upper limb affected, nerves, tendons and bones involved, amputation levels and soft tissue reconstructions including tendon transfers.

**Results**

Over 6000 burns are admitted to our institute each year. 27% are due to electrical injuries and 29% include the upper limb. Electrical burns cause 42% of the deaths in our burns patient population. The vast majority of electrical burns are due to High Voltage electricity in males. 60% are admitted in the first 2 days post injury, but admissions continue up to 4 weeks post burn.

The most common place of injury was the workplace, followed by the home, road side, educational institutes and the playground. The average Total Body Surface Area involved was 15% (range 1-50%): mostly 2nd and 4th degrees. Nearly half necessitate amputations with nearly a third below elbow amputations. 20% include nerve injuries and include loss of tendons. Surgical debridement was usually performed piecemeal and defects covered successfully with local flap. Tendons were buddied, transferred depending on availability and need.

**Conclusion**

Based on our data we offer an upper limb electrical burns classification, which facilitates surgical treatment and reconstruction.

## Poster

### Abstract Topic: Surmounting Challenges in Rehabilitation

#### P1 Effectiveness of Rehabilitation Aide Memoire in Nursing Population

##### Pei Fen Seah^1^ ,Florence Chiang^2^, Wei Xiang Er^1^, Masroor ur Rahman^3^, Si Jack Chong^4^

###### ^1^Department of Physiotherapy, Singapore General Hospital, Singapore; ^2^Department of Occupational Therapy, Singapore General Hospital, Singapore; ^3^Burn & Plastic Surgery Unit, Dhaka Medical College Hospital & Sheikh Hasina Burn Institute, Bangladesh; ^4^The Association for Burn Injuries, Singapore

####### **Correspondence:** Pei Fen Seah

**Background**

Due to insufficient rehabilitation manpower in Bangladesh, the nursing community in Dhaka Medical College Hospital (DMCH) Burn Unit and Sheikh Hasina National Institute of Burn and Plastic Surgery (SHNIBPS) tend to provide only limited basic rehabilitation for their patients. Within this context of an environment lacking professional rehabilitation therapists, an aide memoire would be helpful to guide nurses to provide appropriate and effective rehabilitation for post-burn patients.

**Materials and Methods**

A rehabilitation aide memoire was developed by the SingHealth rehabilitation team and implemented as a standard text for rehabilitation in the DMCH Burn Unit and SHNIBPS in Bangladesh in 2019. To review the usefulness of the aide memoire in improving the knowledge and confidence level of nurses involved in the rehabilitative care of burns patients, nurses who attended the SingHealth Temasek Foundation International Capacity Enhancement for Burn Specialists Training Programme in April 2019 were surveyed before and after reading the rehabilitation aide memoire.

**Results**

A total of 45 nurses were surveyed. 20% of the nurses had ≤1 year of experience in managing burns patients, 64% had >1 to 3 years of experience, 5% had >3 to 5 years of experience and 11% had >5 years of experience. There were statistically significant (p<0.0001) improvements in both knowledge and confidence scores after training, with mean improvements of 1.3 and 1.4 points respectively (range 0-10).

**Conclusions**

The results highlighted that all participants who had read the rehabilitation aide memoire gained better theoretical knowledge of and confidence in performing rehabilitative interventions for patients with burn injuries. We recommend implementing such aide memoires in centres with no or insufficient professional rehabilitation therapists.

### Abstract Topic: _Others_

#### P2 Launching The First Burn Prevention and Awareness Campaign in Bangladesh

##### Andrew Cheah^1^, Tzuemn Ling Low^1^, Chong Xin Ying^2^, Tanveer Ahmed^3^, Chong Si Jack^2^

###### ^1^Dept. of Plastic & Reconstructive Surgery, Singapore General Hospital, Singapore; ^2^The Association for Burn Injuries, Singapore; ^3^Burn & Plastic Surgery Unit\ Sheikh Hasina Burn Institute, Bangladesh

####### **Correspondence:** Andrew Cheah

**Background:** The burden of Burn injury in Bangladesh is significant, every year approximately 800000 people in Bangladesh sustain Burn injuries ^1^ including 173,000 children^2^. Since 2016, Sing-Health and the Bangladesh authorities have been conducting an education program to train and educate specialists and nurses in management of burn injuries. Throughout the program we have conducted studies from these health-care professionals with the aim of launching the first burns nationwide prevention and awareness campaign.

**Materials & Methods:** In an effort to raise awareness effectively, we conducted 2 studies from 2017- 2019 to identify risks for Burn injury specific to the country and their feedback on the material to be used for the campaign. The surveys were conducted in English on Burns health-care professionals.

**Results:** 160 health-care professionals were surveyed, 50% were nurses, 34% were doctors, and 16% were allied-health. 45% of the health-care professionals surveyed have had 1 to 2 years of experience managing Burns patients, 25% had 3 to 5 years of experience and 30% had more than 5 years of experience. 90% of health-care professionals agreed that there was no Burns awareness programme in Bangladesh. 5 dangers for Burn injury were identified, from highest risk to lowest risk: hot water, oil lamps, overloaded power-sockets, exposed overhead electrical cables and kitchen fires.

**Conclusion:** Burn awareness and prevention is key to reducing the incidence and severity of program. Having solicited the feedback and necessary improvements, the team is confident in assisting the Bangladesh authorities in launching a relevant and successful campaign in late 2019.

### Abstract Topic: Others

#### P3 Perception and challenges to establishing the first skin bank in Bangladesh

##### Andrew Cheah^1^, Alvin Chua^1^, Chong Xin Ying^2^, Mahbub Hasan^3^, Chong Si Jack^2^

###### ^1^Dept. of Plastic & Reconstructive Surgery, Singapore General Hospital, Singapore; ^2^The Association for Burn Injuries, Singapore; ^3^Burn & Plastic Surgery Unit, Dhaka Medical College Hospital & Sheikh Hasina Burn Institute, Bangladesh

####### **Correspondence:** Andrew Cheah

**Background:** Skin banks are a vital element of a Burn units’ armamentarium. Clinical utility of allograft skin for wound coverage was described in 1981^1^ and it has been shown to help in the management of severe burns by facilitating early excision and allografting^2^. In Bangladesh, burn injuries are significant with more than 800000 injuries per year^3^ and burn mortality rates are on the rise^4^. Bangladesh has a population of over 160 million but has only one tertiary burn care centre. The set-up of a skin bank would be a useful tool, but issues related to legislation, methods, equipment, storage and distribution need to be addressed. We aim to identify these issues to streamline the successful establishment of the first skin bank in Bangladesh.

**Materials & Methods:** Surveys were conducted in English, on health-care professionals and hospital managers. 5 key areas for the set-up of a skin bank were identified: screening, harvesting or recovery of skin, processing of skin, storage and finally the distribution of skin.

**Results:** The surveys had 40 respondents. 80% of respondents were Doctors. 95% of respondents felt that there was a role for a skin bank in Bangladesh. 80% of those surveyed cited lack of equipment as a main issue of skin banking in Bangladesh. 25% felt there were legal issues that needed to be addressed first. Religious or cultural issues were identified as issues by 50% and 25% of respondents respectively. When asked about the 5 key areas individually, lack of equipment was again cited as a main issue when it came to the screening, processing, storage and distribution of skin. Religious or cultural issues were cited as issues in the harvesting or recovery of skin.

**Conclusion:** The successful establishment of a skin bank raises several pertinent issues related to legislation, religion, culture and the need for specialized training and equipment.

### Abstract Topic: Others

#### P4 Knowledge on Burn Prevention and First Aid for burn injuries amongst Airport Firefighters

##### Chong Xin Ying^2^, Andrew Cheah^1^, Kok Yee Onn^1^, Janna Joethy^1^, Chong Si Jack^2^

###### ^1^Department of Plastic & Reconstructive Surgery, Singapore General Hospital, Singapore; ^2^The Association for Burn Injuries, Singapore

####### **Correspondence:** Andrew Cheah

**Background**

The Changi Airport firefighters are a group of skilled professionals , whose core responsibilities lies with swift and responsive action to mitigate fires in the airport, extricate and provide first aid for victims in large scale incidents. The authors conducted professional training in burns injuries management for this 250 strong gorup. They conducted a cohort study to understand the firefighters’ knowledge of burns prevention and exmained if a simple acronym BURNS can help the firefighters remember the key steps for burn first aid

**Methodology**

A total of 250 firefighters took part in this study. This voluntary study was conducted electronically via an online survery system and the results were analysed.

**Results**

There was an improvement in burns prevention awareness following exposure to the simple burns prevention awareness poster. The simple acronym of BURNS (Back Away, Uncover, Rinse under running water, eNclose the wound with a clean dressing and Seek medical help) helped (statistically significantly) the firefighters remember the crucial 5 steps in burn first aid

**Conclusion**

An electronic online survery system is a simple , environmentally-friendly system which facilitated the conduct of the study. A simple burn prevention awareness programme refreshed the firefighters’ memory of the common causes of burns injuries. A uncomplicated acronym (BURNS) helped them significantly to remember the crucial steps for first aid. This can be part of all uniformed groups training programmes.

### Abstract Topic: Others

#### P5 Burn Aid: Improving Burn first aid knowledge and practice in Bangladesh

##### Andrew Cheah^1^, Luther Chung^2^, Nurunnahar Lata^3^, Chong Xin Ying^4^, Chong Si Jack^4^

###### ^1^Department of Plastic & Reconstructive Surgery, Singapore General Hospital, Singapore; ^2^School of Medicine, Monash University, Australia; ^3^Burn & Plastic Surgery Unit, Dhaka Medical College Hospital & Sheikh Hasina Burn Institute, Bangladesh; ^4^The Association for Burn Injuries, Singapore

####### **Correspondence:** Andrew Cheah

**Background**

Burns first aid is a crucial step in the management of Burn injuries. It limits thermal or chemical damage, reduces subsequent inflammation and delays burn progression or conversion1. The burden of Burn injury in Bangladesh is significant, with approximately 800000 burn injuries per year2. Successful treatment starts with effective first aid. We aim to assess healthcare professionals’ knowledge of first aid and improve practice in Bangladesh by implementing simple mnemonic posters to help guide initial management.

**Methods**

In conjunction with our regional Burn prevention and management training program, we conducted surveys in English, on burns healthcare professionals. The survey was performed pre and post implementation of a Burns First-Aid Mnemonic poster.

**Results**

A total of 125 healthcare professionals were surveyed over a 2-year time frame. 40% of respondents were Doctors, 48% were Nurses and 12% were Allied-Health. Majority had 1 to 2 years of experience managing burns patients. 92% were not aware of any first aid guidelines, 8% quoted the rudimentary “stop, drop and roll” as a first aid guideline. Common problems with Burns first aid that were highlighted included delay in seeking treatment, poor accessibility to healthcare and enclosing wounds with traditional medicines. Pre-implementation of our mnemonic posters, 60% of respondents were able successfully identify first aid in the correct order. Post-implementation, 97% of respondents were able to correctly identify Burns first aid in the correct order. 96% of respondents felt the poster was useful in guiding first aid management.

**Conclusion**

Burns first aid is a crucial step to the management of Burn injuries. The initial step to decreasing the morbidity and burden of burn injuries is to increase awareness of appropriate first aid. Posters and simple education steps have been shown to be helpful.

### Abstract Topic: Others

#### P6 A descriptive, cross-sectional study investigating the knowledge and perception regarding infection prevention and control among doctors and nurses in Dhaka National Institute of Burns and Plastic Surgery, Bangladesh

##### Darshini Devi Rajasegeran^1^, Lee Lay Eng^1^, Maitra Jhuma^2^, Bristi Mitra Kundu^2^, Tanveer Ahmed Himon^2^

###### ^1^Singapore General Hospital, Singapore; ^2^Dhaka National Institute of Burns and Plastic Surgery, Bangladesh

####### **Correspondence:** Darshini Devi Rajasegeran

**Background**

Infection is the foremost cause of morbidity and mortality in Dhaka National Institute of Burns and Plastic Surgery, Bangladesh. With the construction of a new institute, a key initiative is to develop their first infection control unit. As infection control requires conscious effort and practice, understanding the knowledge and perception of healthcare professionals is crucial

**Materials and Methods**

This is a descriptive, cross-sectional study investigating the knowledge and perception regarding infection prevention and control among doctors and nurses. A self-administered questionnaire was drawn from research and WHO Perception Survey for Healthcare Workers. It consisted of closed, Likert scale and open-ended questions on knowledge, perception, obstacles and possible solutions.

**Results**

While all participants (n=33) agreed that infection control reduces mortality, 28.6% of doctors (n=7) reported that hand hygiene (HH) was not the best measure for infection control. 73.1% of nurses (n=26) and only 28.6% doctors reported that HH required a lot of effort. However, 53.8% nurses reported being able to perform HH during recommended situations, while doctors (100%) reported not being able to do so. Nonetheless, nurses (73.1%) and doctors (57.1%) reported that fulfilling tasks was more important than HH when busy. Nurses (100%) reported that it was important for management and fellow healthcare professionals that they perform optimal HH. Two- fifths of doctors reported that it was not important. Out of products, product not in convenient locations were top responses for not practicing HH. Supportive management, education, clear and simple instructions, feedback or audits and hand rubs made available at all point of care were reported as effective measures.

**Conclusions**

There is a prevailing notion that fulfilling duties takes precedence over infection control. There are also differing viewpoints on how management and healthcare professionals perceive infection control. Personal ownership, management’s stance, education and adequate amenities are suggested solutions for current obstacles

