# Correction to: Abstracts from the 12th Asia Pacific Burn Congress

**DOI:** 10.1186/s41038-019-0172-1

**Published:** 2019-11-28

**Authors:** Supplement Organiser: 12th Asia Pacific Burn Congress (12th APBC)

**Affiliations:** Secretariat to 12th APBC Organising Committee, working for Association for Burn Injuries Singapore, Singapore, Singapore

**Correction to: Burns Trauma (2019) 7**

**https://doi.org/10.1186/s41038-019-0164-1**

Following publication of the supplement *Abstracts from the 12th Asia Pacific Burn Congress* [1], all affiliations have been revised for legal reasons.

The original supplement has been updated.
